# The catalytic role of glutathione transferases in heterologous anthocyanin biosynthesis

**DOI:** 10.1038/s41929-023-01018-y

**Published:** 2023-08-31

**Authors:** Michael Eichenberger, Thomas Schwander, Sean Hüppi, Jan Kreuzer, Peer R. E. Mittl, Francesca Peccati, Gonzalo Jiménez-Osés, Michael Naesby, Rebecca M. Buller

**Affiliations:** 1 Competence Center for Biocatalysis, Zurich University of Applied Sciences, Wädenswil, Switzerland; 2 Department of Biotechnology, Delft University of Technology, Delft, Netherlands; 3 Department of Biochemistry, University of Zurich, Zurich, Switzerland; 4 Center for Cooperative Research in Biosciences, Basque Research and Technology Alliance, Derio, Spain; 5 Ikerbasque, Basque Foundation for Science, Bilbao, Spain; 6 Lantana Bio, Toulouse, France

**Keywords:** Biocatalysis, Synthetic biology, X-ray crystallography, Mechanism of action

## Abstract

Anthocyanins are ubiquitous plant pigments used in a variety of technological applications. Yet, after over a century of research, the penultimate biosynthetic step to anthocyanidins attributed to the action of leucoanthocyanidin dioxygenase has never been efficiently reconstituted outside plants, preventing the construction of heterologous cell factories. Through biochemical and structural analysis, here we show that anthocyanin-related glutathione transferases, currently implicated only in anthocyanin transport, catalyse an essential dehydration of the leucoanthocyanidin dioxygenase product, flavan-3,3,4-triol, to generate cyanidin. Building on this knowledge, introduction of anthocyanin-related glutathione transferases into a heterologous biosynthetic pathway in baker’s yeast results in >35-fold increased anthocyanin production. In addition to unravelling the long-elusive anthocyanin biosynthesis, our findings pave the way for the colourants’ heterologous microbial production and could impact the breeding of industrial and ornamental plants.

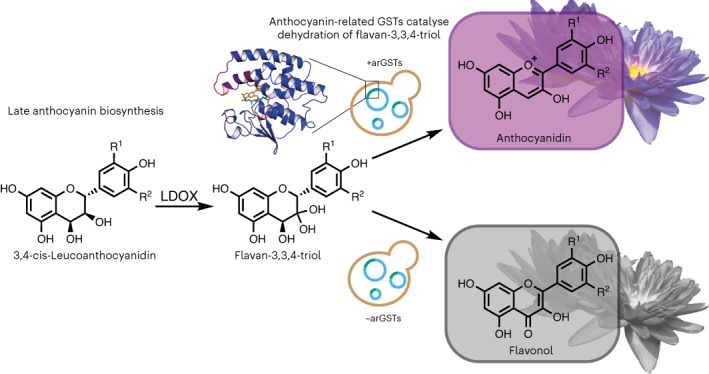

## Abstract (web summary)

Anthocyanins are used in the food and cosmetic industries. Due to the insufficient production in alternative hosts, they are still isolated from plants. Now, this study suggests an important catalytic role of glutathione transferases for the efficient biosynthesis of these natural products.

## Main

Anthocyanins (**1**) are soluble pigments almost universally distributed amongst angiosperms (flowering plants), where they are responsible for the red, purple and blue colours of most flowers, fruits and leaves^1,2^. Due to their diverse colour expression and many proposed health effects^3^, their industrial adoption as natural colourants and bioactive compounds in the food, nutraceutical and cosmetic industries is progressively growing^4,5^. Currently, large-scale production of the pigments is achieved through extraction from plant raw materials with high contents of stable anthocyanins (**1**), for example, purple sweet potato, black carrot or red cabbage^5^, despite the associated issues of sustainability and supply. Thus, the construction of microbial cell factories for anthocyanin production is a field of great industrial interest; however, commercially viable product titres have not yet been reached, partly due to issues arising from the late steps of the biosynthetic pathway^6,7^.

**Extended Data Fig. 1:**
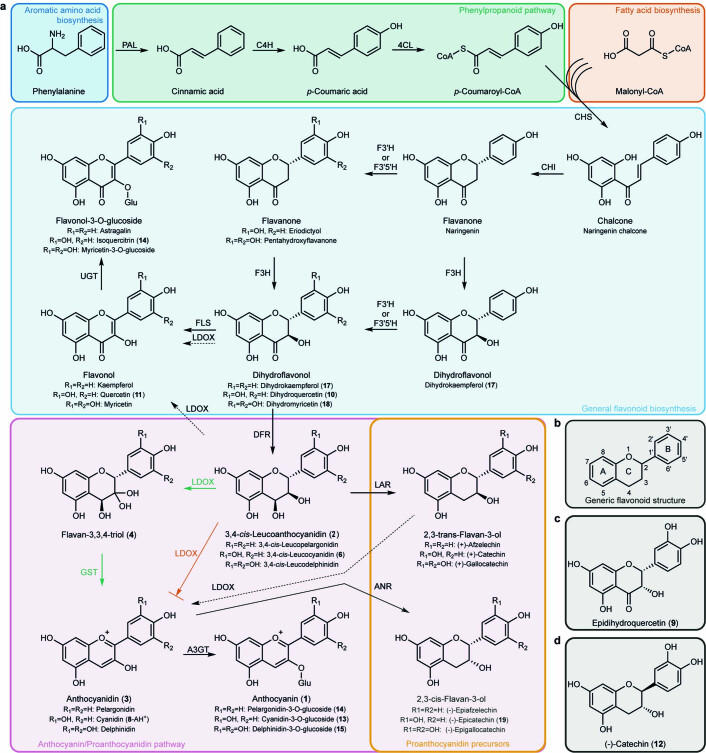
Biosynthetic pathway of anthocyanins. **a**, Biosynthetic pathway of anthocyanins (**1**) in plants from the primary metabolites phenylalanine and malonyl-CoA. The proposed enzymatic reaction sequence is highlighted with green arrows, whereas the generally proposed pathway is shown with an orange arrow. Dashed arrows represent reported side-reactivities of LDOX observed *in vitro*. **b**, Generic structure of the flavonoid skeleton, including numbering and ring nomenclature. **c**, Structure of epidihydroquercetin (**9**), a side-product of *in vitro* reactions with LDOX from 3,4-*cis*-leucocyanidin (**6**) usually not found in plants. **d**, Structure of (-)-catechin (**12**), which was used for crystallization of *Pt*GSTF8. Abbreviations: ANR: Anthocyanidin reductase; A3GT: Anthocyanidin-3-*O*-glycosyl transferase; CHI: Chalcone isomerase; CHS: Chalcone synthase; C4H: Cinnamate-4-hydroxylase; CPR: Cytochrome P450 reductase; DFR: Dihydroflavonol-4-reductase; FLS: Flavonol synthase; F3H: Flavanone-3-hydroxylase; F3΄H: Flavonoid-3΄-hydroxylase; F3΄5΄H: Flavonoid-3΄,5΄-hydroxylase; GST: Glutathione transferase; LAR: Leucoanthocyanidin reductase; LDOX: Leucoanthocyanidin dioxygenase; PAL: Phenylalanine ammonia lyase; 4CL: 4-Coumarate-CoA ligase.

The penultimate step in the anthocyanin biosynthetic pathway (Extended Data Fig. 1) from 3,4-*cis*-leucoanthocyanidins (**2**) to anthocyanidins (**3**) is thought to be catalysed by the Fe/α-ketoglutarate-dependent dioxygenase leucoanthocyanidin dioxygenase (LDOX), also called anthocyanidin synthase (ANS)^8,9^. Based on a protein crystal structure^10^, biochemical analysis^11–14^ and feeding of isotopically labelled precursors in plants^15^, LDOX is proposed to catalyse an α-face C-3 hydroxylation of leucoanthocyanidins (**2**). Notably, however, the unstable product flavan-3,3,4-triol (**4**) is converted mainly into the by-products epidihydroquercetin (**9**), dihydroquercetin (**10**) and quercetin (**11)**^13^, while the transformation into the coloured anthocyanidin has only been observed as a minor side reaction in vitro^11–13^ or in vivo by engineered microorganisms^6,16^.

Another debated aspect of the anthocyanin pathway is the molecular function of anthocyanin-related glutathione transferases (arGSTs)^17,18^. Loss-of-function mutations or downregulation of genes encoding arGSTs result in strongly decreased accumulation of anthocyanins (**1**) in a wide range of plants^19–28^, including reduced fruit colouration in cultivated *Fragaria vesca* (strawberry) through transient knock-down of reduced anthocyanins in petioles (*RAP*)^20^ or white *Prunus persica* (peach) flowers caused by an arGST encoding regulator involved in anthocyanin transport (*Riant*) gene with a 2 bp insertion leading to a premature stop codon^21^. In some cases the phenotype is linked to mislocalization of the pigments to the cytoplasm^29^ or vesicle-like structures^19,30^. While arGSTs were shown to catalyse the nucleophilic transfer of the tripeptide glutathione (GSH) onto the model substrate 1-chloro-2,4-dinitrobenzene (CDNB) (**5**) in vitro, such GSH conjugates with anthocyanins (**1**) were never observed in vitro or in planta^19,31,32^. In contrast, arGSTs bind to various flavonoids in vitro^19,26,31^ and localize on membranes in tissues with high anthocyanin (**1**) production in planta^19,30^. This evidence has inspired the ligandin model for vacuolar sequestration of anthocyanins (**1**), in which arGSTs are believed to protect the labile anthocyanidins (**3**) from degradation and/or guide them from their biosynthetic origin on the cytoplasmic surface of the endoplasmic reticulum to transporters located on the tonoplast membrane^18,19,31^.

Yet, the results of several plant studies on the involvement of arGSTs in anthocyanin and oligomeric proanthocyanidin accumulation cannot be fully explained by the ligandin model. For example, when introduced via microparticle bombardment, cDNA encoding the arGST from *Zea mays* (*ZmBZ2*) can complement mutations of the corresponding genes encoding arGST (*PhAN9*) in *Petunia* spp., and vice versa. Notably, the pigmented aleurone (*Zea mays*) or corolla (*Petunia*) spots—indicative of functional complementation—have a dark purple centre and a halo of paler but distinctly pigmented cells^28^ suggesting that the result of arGSTs *Zm*BZ2 and *Ph*AN9 activity goes beyond intracellular transport. Additionally, epicatechin (**19**), a starter unit for proanthocyanidins^33^, is produced cytosolically from cyanidin (**8-AH**^**+**^) by the enzyme anthocyanidin reductase (ANR) (Extended Data Fig. 1). Notably, in *Arabidopsis thaliana* the mutation of arGST *tt19* completely eliminates production of epicatechin^34^—an unexpected outcome in the frame of the ligandin model as the cytosolic availability of cyanidin (**8-AH**^**+**^), the precursor molecule of epicatechin (**19**), should be unaffected by mutating a transport protein.

In this Article, we show that arGSTs have a catalytic role in heterologous anthocyanin biosynthesis. Using *Pt*GSTF8 from poplar as a model enzyme, we use biochemical, structural and computational methods to demonstrate that arGSTs employ a GSH-dependent Brønsted base mechanism for conversion of the LDOX product flavan-3,3,4-triol (**4**) or flavan-3-on-4-ol (**7**) into flav-2-en-3,4-diol (**8-B4**), an equilibrium form of cyanidin. In *Saccharomyces cerevisiae* microbial cell factories constructed for anthocyanin biosynthesis, inclusion of selected arGSTs boosts the cyanidin-3-*O*-glucoside (**13**) titre by 36.5-fold. In addition, we show that heterologous production of pelargonidin-3-*O*-glucoside (**14**) and delphinidin-3-*O*-glucoside (**15**) can be similarly enhanced.

## Results

### Characterization of *At*LDOX product

**Fig. 1:**
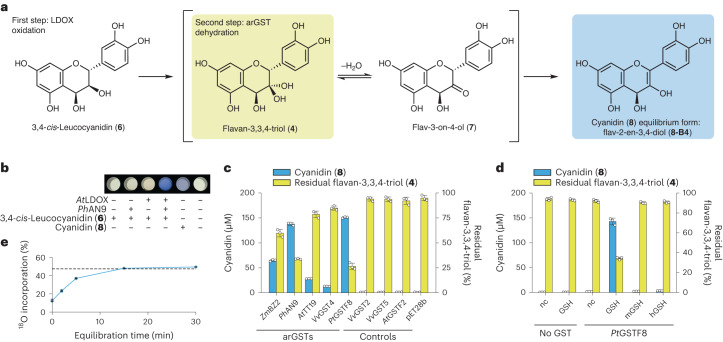
arGSTs catalyse formation of cyanidin. **a**, Proposed enzymatic reactions from 3,4-*cis*-leucocyanidin (**6**) to flav-2-en-3,4-diol (**8-B4**), the 4-hydrated form within the reaction network of cyanidin in solution (Extended Data Fig. 4). **b**, Blue colour formation in coupled enzymatic reactions from 3,4-*cis-*leucocyanidin (**6**) using *At*LDOX and *Ph*AN9. **c**, Cyanidin (**8**) formation from ultrafiltered flavan-3,3,4-triol (**4**) with clarified *E. coli* lysates expressing GSTs of varying plant origins. **d**, Effect of GSH and selected cofactor analogues (mGSH and hGSH) at 20 μM on the conversion of ultrafiltered flavan-3,3,4-triol (**4**) to cyanidin (**8**) employing purified apo-*Pt*GSTF8. nc, no cofactor; mGSH, *S*-methylglutathione; hGSH, *S*-hexylglutathione. **e**, Percentage of ^18^O incorporation into cyanidin (**8**) as a function of *Pt*GSTF8 addition at varying time points after dilution of ultrafiltered flavan-3,3,4-triol (**4**) into H_2_^18^O containing buffer. The dashed line marks the H_2_^18^O content of the reaction buffer. Data in **c**–**e** are the mean values ± s.d. of three independent replicates. Source data

**Extended Data Fig. 2:**
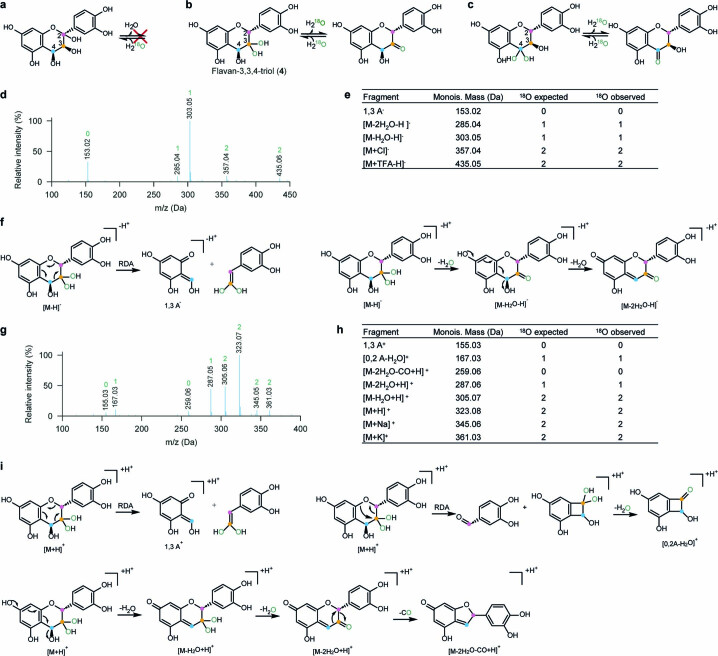
Mass spectrometric analysis of the LDOX oxidation product flavan-3,3,4-triol (4). **a,b,c** Mechanistically, the *At*LDOX catalysed hydroxylation of 3,4-*cis*-leucocyanidin (**6**) may result in hydroxylation products at C_2_ (**a**), C_3_ (**b**) or C_4_ (**c**). **a**, Hydroxylation of C_2_ would preclude the incorporation of an O^18^ label as no equilibrium between a ketone and a gem-diol form exists for this hydroxylation product. **b,c**, Hydroxylation at either C_3_ (**b**) or C_4_ (**c**) allows the incorporation of the heavy oxygen isotope at the corresponding positions. **d,g**, Negative (**d**) and positive (**g**) ion ESI-MS spectrum of the *At*LDOX oxidation product from 3,4-*cis*-leucocyanidin (**6**). The green labels show the observed ^18^O incorporations into the fragments when reactions are performed with H_2_^18^O. The observed incorporation of up to two O^18^ labels rules out formation of the C_2_ hydroxylated product (**a**). **e,h**, Observed adducts and fragments in negative (**e**) and positive (**h**) ion ESI-MS, their calculated monoisotopic masses, as well as the expected and observed number of ^18^O incorporations. **f**,**i**, Proposed fragmentation pathways of flavan-3,3,4-triol (**4**) for fragments observed in negative (**f**) and positive (**i**) ion ESI-MS. For fragment [1,3 A^-^] (**d,e,f**) and fragment [1,3 A^+^] (**g,h,i**) comprising C_4_, the incorporation of an O^18^ label is not observed, while fragment [0,2 A-H2O]^+^ (**g,h,i**), including C_3_, contains an O^18^ label supporting formation of flavan-3,3,4-triol (**4**). Source data

**Extended Data Fig. 3:**
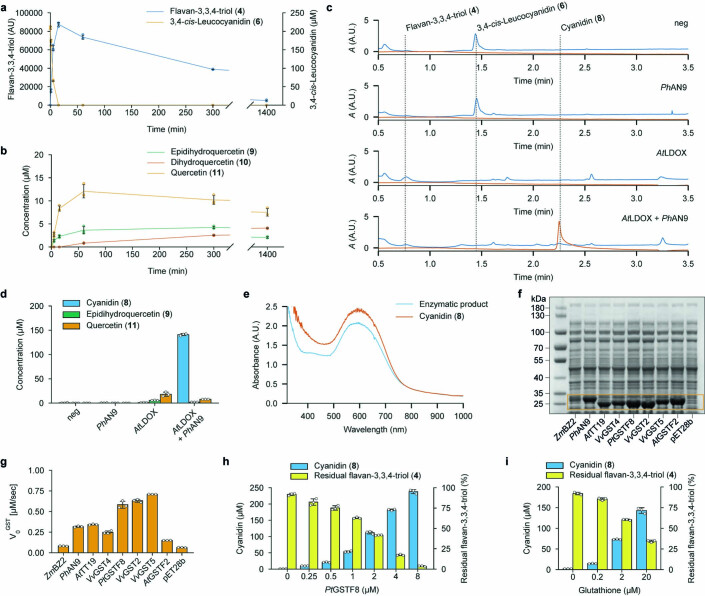
arGSTs catalyse the conversion of flavan-3,3,4-triol (4) into cyanidin (8). **a**, Time course of formation of flavan-3,3,4-triol (**4**) from 3,4-*cis*-leucocyanidin (**6**) using purified *At*LDOX. **b**, Timecourse of formation of side products from 3,4-*cis*-leucocyanidin (**6**) using purified *At*LDOX. **c**, HPLC-UV traces of coupled reactions using clarified *E.coli* cell lysates expressing *Ph*AN9 and *At*LDOX with 3,4-*cis*-leucocyanidin (**6**). Negative control reactions were performed with buffer in place of the purified enzymes (neg). Blue: 280 nm, Red: 500 nm. **d**, Quantification of reaction products of coupled reactions using clarified *E.coli* cell lysates expressing *Ph*AN9 and *At*LDOX with 200 μM 3,4 *cis*-leucocyanidin (**6**). Negative control reactions were performed with buffer in place of the purified enzymes (neg). **e**, Comparison of UV-Vis spectra of the enzymatic product and authentic cyanidin (**8**) in reaction buffer. **f**, SDS^−^Page of clarified *E.coli* lysates expressing different GSTs. **g**, GSH-transferase activity of *E.coli* lysates expressing different GSTs towards the model substrate CDNB (**5**). **h**, Conversion of ultrafiltered flavan-3,3,4-triol (**4**) into cyanidin (**8**) using purified *Pt*GSTF8 at different concentrations. **i**, Conversion of ultrafiltered flavan-3,3,4-triol (**4**) into cyanidin (**8**) using 2 μM purified *Pt*GSTF8 and different concentrations of GSH. Data of panels **a,b,d**,**g,h,i** are mean values ± standard deviation of three independent replicates. Source data

To elucidate the late steps of anthocyanidin biosynthesis, we set out to more closely investigate the LDOX oxidation product of 3,4-*cis*-leucocyanidin (**6**). This compound was only recently characterized in in vitro reactions with LDOX from *Vitis vinifera* (*Vv*LDOX) and proposed as flavan-3,3,4-triol (**4**) based on HPLC–MS analysis and the observed degradation products^13^. In our hands, reactions performed with purified LDOX from *A. thaliana* (*At*LDOX) led to an enzymatic product with an equivalent MS spectrum to the literature report (Extended Data Fig. 2d). As observed previously^13^, the putative flavan-3,3,4-triol (**4**) was not stable during purification, and confirmatory time-course experiments showed almost complete degradation of the intermediate (**4**) within 24 h (Extended Data Fig. 3a). Thus, to gather additional evidence about the identity of the LDOX oxidation product, we performed enzymatic reactions in buffer containing H_2_^18^O, exploiting the expected equilibrium between the gem-diol flavan-3,3,4-triol (**4**) and the corresponding ketone flavan-3-on-4-ol (**7**) (Fig. 1a). In aqueous media, incorporation of the isotopic label from bulk solvent into flavan-3,3,4-triol (**4**) was anticipated to lead to distinct *m*/*z* and ^18^O incorporation patterns of the observed adducts and flavonoid-typical C-ring cleavage fragments. The *At*LDOX oxidation product was analysed via ESI-MS in positive and negative modes, leading to spectra in accordance with a geminal diol at C3 (Extended Data Fig. 2), further confirming flavan-3,3,4-triol (**4**) as the oxidation product of LDOX from 3,4-*cis*-leucocyanidin (**6**). As expected, the reactions with *At*LDOX also resulted in formation of epidihydroquercetin (**9**), dihydroquercetin (**10**) and quercetin (**11**), the known by-products of the investigated reaction^12,13^ (Extended Data Figs. 1a,c and 3b).

### arGSTs catalyse formation of cyanidin

**Extended Data Fig. 4:**
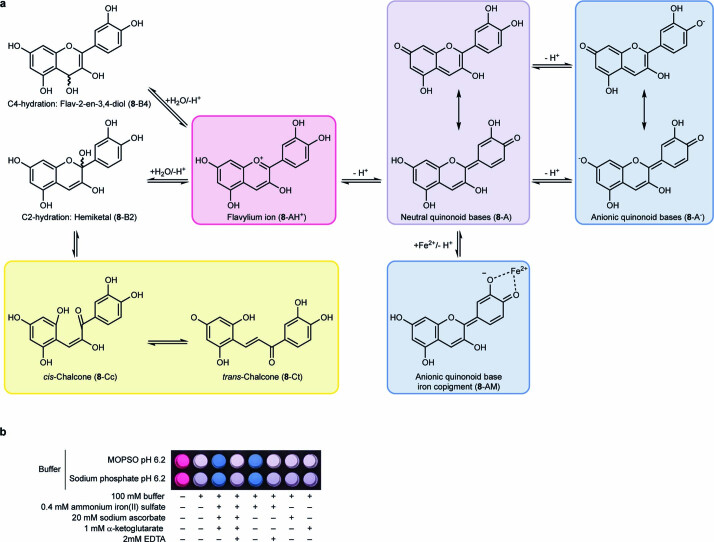
Reaction network of cyanidin (8). **a**, Equilibrium reaction network of cyanidin (**8**) in solution. The boxes show the colours of the different species. **b**, Colour expression of authentic cyanidin (**8**) in reaction buffers with different combinations of co-factors and EDTA, showing the formation of the blue iron co-pigment of the anionic quinonoid base (**8**-AM).

arGSTs are essential for the accumulation of anthocyanins (**1**) in plants^19–28^. The observed inconsistencies between available in planta, in vitro and in vivo (cell factory) data, however, led us to hypothesize that arGSTs might not (only) have the literature-proposed transport function but could play a central role in the biosynthetic step from leucoanthocyanidins (**2**) to anthocyanidins (**3**). Thus, unlike previous in vitro biochemical studies or metabolic engineering efforts, which had excluded arGSTs, we set out to confirm our hypothesis by performing coupled enzymatic reactions with 3,4-*cis*-leucocyanidin (**6**) combining clarified *Escherichia coli* cell lysates expressing *At*LDOX and the well-studied arGST *Ph*AN9 from petunia. The reaction in the presence of both enzymes resulted in the formation of a partially soluble blue pigment with the same *λ*_max_ as authentic cyanidin (**8**) in reaction buffer (Fig. 1b and Extended Data Fig. 3e). Reconstitution experiments with different buffer components suggested that the blue pigment was an iron co-pigment of the quinoidal base anion form of cyanidin (**8**-AM) (Extended Data Fig. 4a,b). HPLC–MS analysis after solubilization with acidified methanol confirmed an almost stoichiometric conversion of 3,4-*cis*-leucocyanidin (**6**) into cyanidin (**8**), while the formation of the side products epidihydroquercetin (**9**), and quercetin (**11**) was reduced (Extended Data Fig. 3c,d).

In the early steps of the anthocyanin pathway, chalcone isomerase-like proteins modulate the product specificity of chalcone synthase through protein–protein interactions^35^. To determine whether arGSTs have such a modulating activity on LDOX, we ran two-step reactions, in which we separated *At*LDOX from the flavan-3,3,4-triol (**4**) product through ultrafiltration before addition of *Ph*AN9-clarified cell lysate. Again, this experiment resulted in the conversion of flavan-3,3,4-triol (**4**) to cyanidin (**8**), suggesting a catalytic rather than a modulating role of the arGSTs in the further processing of the semi-stable LDOX product. Importantly, we established that ultrafiltered flavan-3,3,4-triol **(4**) was converted to cyanidin (**8**) by additional well-characterized arGSTs from maize (*Zm*BZ2), arabidopsis (*A*tTT19) and grapevine (*Vv*GST4), and from the structure-elucidated phi-class orthologue from poplar (*Pt*GSTF8), while application of GSTs unrelated to anthocyanin biosynthesis (*Vv*GST2, *Vv*GST5, *At*GSTF2) did not result in cyanidin (**8**) formation (Fig. 1c). To exclude false negatives, all investigated GSTs were confirmed to be solubly and functionally produced by SDS–PAGE analysis and an activity test with the generic GST substrate CDNB (**5**) (Extended Data Fig. 3f,g).

### Biochemical characterization of *Pt*GSTF8

**Extended Data Fig. 5:**
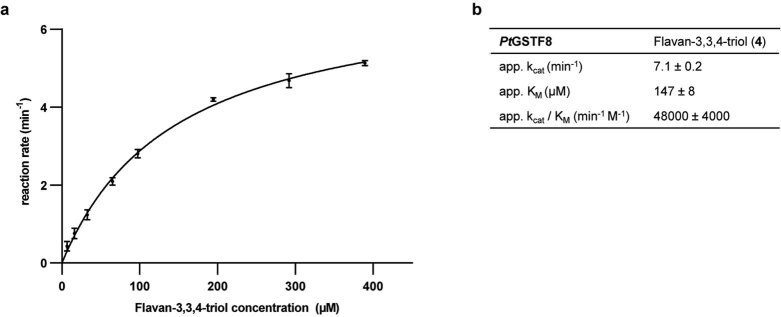
Michaelis–Menten-Kinetics of *Pt*GSTF8 with flavan-3,3,4-triol (4). **a**, Michaelis–Menten plot of measured reaction rate at different substrate concentration. **b**, Table of apparent Michaelis–Menten parameters. Data of panels **a, b** are mean values ± standard deviation of three independent replicates. Source data

To characterize the enzymatic function of arGSTs in the anthocyanin pathway in more detail, we decided to use the structure-elucidated *Pt*GSTF8 as a representative model enzyme in our biochemical assays. After expression of *Pt*GSTF8 in *E. coli*, we reduced the reported disulfide bond between the active site cysteine C13 and GSH in the clarified cell lysate using dithiothreitol (DTT) and removed the reduced GSH with an extended on-column washing step during purification. Using these enzyme preparations with a defined GSH concentration (20 μM) in the established two-step in vitro reactions, we found that the extent of conversion of the ultrafiltered flavan-3,3,4-triol (**4**) depended on the applied concentration of the purified *Pt*GSTF8, confirming that this enzyme is required and sufficient for the observed conversion to cyanidin (Extended Data Fig. 3h). Recording of Michaelis–Menten kinetics of *Pt*GSTF8 with flavan-3,3,4-triol (**4**) revealed an apparent catalytic rate constant (*k*_cat_) of 7.1 min^−1^ and a Michaelis constant (*K*_m_) of 147 ± 8 μM (Extended Data Fig. 5) in line with catalytic parameters described for enzymes active in the secondary metabolism^36^. In addition, our experiments revealed that the enzymatic reaction strictly required the addition of catalytic amounts of GSH (Extended Data Fig. 3i). In contrast, addition of *S*-substituted methyl- and hexylglutathione did not result in the formation of cyanidin (**8**) (Fig. 1d), implying an involvement of the thiol group of GSH in catalysis.

**Extended Data Fig. 6:**
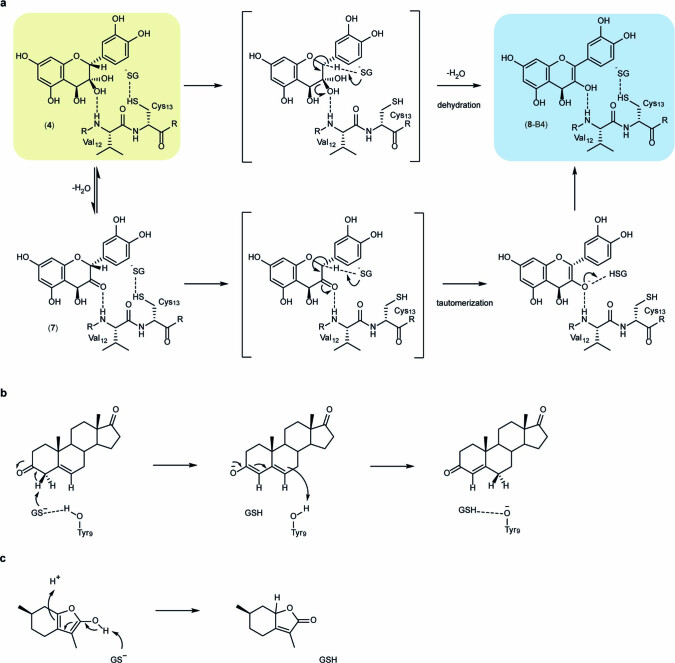
Proposed Brønsted base mechanism of GST catalysed reactions. **a**, Proposed mechanisms for the dehydration of flavan-3,3,4-triol (**4**) to flav-2-en-3,4-diol (**8**-B4) by arGSTs. **b**, Proposed mechanism for the isomerization of 5-androsten-3,17-dione by GST A3-3^43^. **c**, Proposed mechanism for tautomerization of (R)-2-hydroxymenthofuran by GST A1-1^44^.

To shed more light on the identity of the substrate of arGSTs, we performed labelling experiments with H_2_^18^O, exploiting the same principle of ^18^O incorporation as described earlier. In detail, we diluted ultrafiltered flavan-3,3,4-triol (**4**) into H_2_^18^O-labelled reaction buffer to initiate ^18^O incorporation at C-3 of the flavonoid structure. By adding purified *Pt*GSTF8 at varying time points, we expected to halt the incorporation of the isotopic label into the final product cyanidin (**8**). As hypothesized, the ^18^O content in cyanidin (**8**) increased as a function of equilibration time and the expected level (47.4%) for stochastic incorporation of a single ^18^O label was reached after 15 min of pretreatment (Fig. 1e). These results confirmed that the enzymatic reaction catalysed by *Pt*GSTF8 terminated the hydration equilibrium between flavan-3,3,4-triol (**4**) and flavan-3-on-4-ol (**7**), leading us to propose a GST-dependent dehydration of flavan-3,3,4-triol (**4**) or a tautomerization of flavan-3-on-4-ol (**7**) to form flav-2-en-3,4-diol (**8**-B4), an equilibrium form of anthocyanins (**1**) in solution (Fig. 1a and Extended Data Figs. 4a and 6a)^37,38^.

### (−)-Catechin bound structure of *Pt*GSTF8

**Fig. 2:**
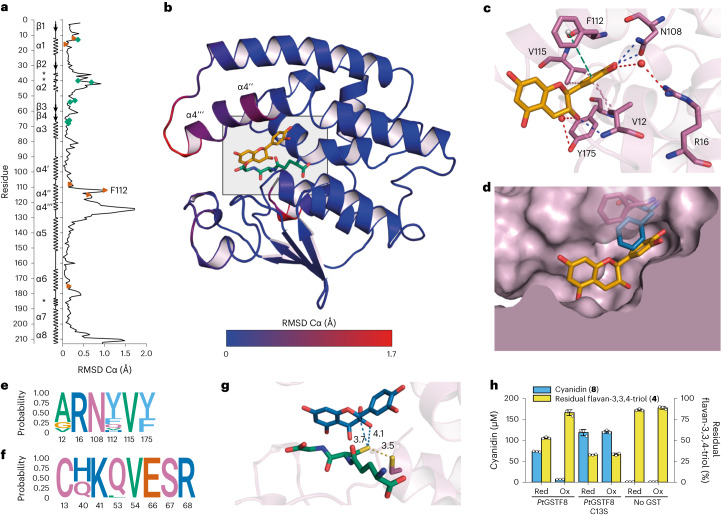
Structural basis of dehydratase activity of *Pt*GSTF8. **a**, RMSD (Cα) between *Pt*GSTF8 as a ternary complex with bound (−)-catechin (**12**) and GSH, and as a binary complex without **12** (PDB 5F07). Green diamonds, G-site; red triangles, H-site; asterisk, 3_10_ helix. **b**, Overall structure of *Pt*GSTF8 as ternary complex with GSH (green) and (−)-catechin (**12**) (orange). The ribbon is coloured according to RMSD (Cα) to the structure of *Pt*GSTF8 as binary complex. The active pocket is highlighted with a grey square. **c**, H-site interactions to (−)-catechin (**12**) (orange). Dashes are coloured according to the interaction type: blue, hydrogen bonds; red, water bridges; green, *π*–*π* interactions; magenta, hydrophobic interactions. **d**, Surface representation of H-site of ternary complex with GSH and (−)-catechin (**12**) (orange). F112 is shown in the conformations from the ternary complex with **12** (magenta) and the binary complex (blue) without **12**. The surface of F112 is shown transparently. **e**,**f**, Sequence logo highlighting the probability of amino acid occurrence within the H-site (**e**) and G-site (**f**) of 24 published arGSTFs. **g**, Docking of flavan-3,3,4-triol (**4**) (blue) into the active site of *Pt*GSTF8 and distances (Å) from the GSH (green) thiolate to C-2 and O-3 of **4** and distance of the C-13 thiolate (purple) to GSH (green). **h**, Dehydratase activity of *Pt*GSTF8 wild type and C13S variants towards ultrafiltered flavan-3,3,4-triol (**4**) before and after oxidation with GSSG. Data in **h** are mean values ± s.d. of three independent replicates. Red, reduction; Ox, oxidation. Source data

**Extended Data Fig. 7:**
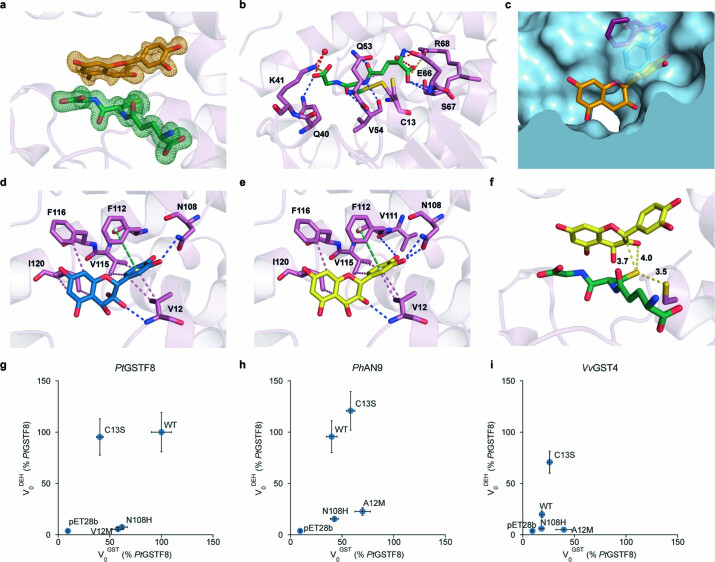
Structural basis of *Pt*GSTF8 dehydratase activity. **a**, *F*_o_ – *F*_c_ composite omit maps of glutathione (green) and (-)-catechin (**12**) (orange) in the ternary complex rendered at 3σ. **b**, G-site interactions to GSH. Blue: hydrogen bond, red: water bridge, green: p-p interaction, magenta: hydrophobic interaction. **c**, Surface representation of H-site of *Pt*GSTF8 crystallized without (-)-catechin (**12**) (PDB:5F07). F112 is shown in the conformations in crystal structures obtained without (blue) and with (magenta) (-)-catechin (**12**) and its surface is rendered transparently. (-)-catechin (**12**) is superimposed from the ternary complex. **d,e**, H-site interactions to docked flavan-3,3,4-triol (**4**) (**d**, blue) and flavan-3-on-4-ol (**7**) (**e**, yellow). Dashes are coloured according to the interaction types: Blue: hydrogen bond, red: water bridge, green: π-π interaction, magenta: hydrophobic interaction. **f**, Docking of flavan-3-on-4-ol (**7**) (yellow) into the active site of *Pt*GSTF8 and distances (in Å) from the GSH (green) thiolate to C-2 and O-3 of **7** as well as distance of the C13 thiolate (purple) to GSH (green) **7**. **g,h,i**, Dehydratase activity towards ultrafiltered flavan-3,3,4-triol (**4**) and GSH-transferase activity towards CDNB (**5**) of variants of *Pt*GSTF8 (**g**), *Ph*AN9 (**h**), *Vv*GST4 (**i**) measured in colorimetric 96-well-plate assays. Data of panels **g,h,i** are mean values ± standard deviation of three independent replicates. Source data

To better understand the catalytic machinery of arGSTs, we determined the crystal structure of *Pt*GSTF8 as a ternary complex with GSH and the substrate analogue (−)-catechin (**12**) (Extended Data Fig. 1d) to a resolution of 1.09 Å (Fig. 2b, Extended Data Fig. 7a and Extended Data Table 1). The structure comprises the typical GST fold with an N-terminal thioredoxin-like domain (β_1_α_1_β_2_α_2_β_3_β_4_α_3_) and an all-helical C-terminal domain (α_4_α_5_α_6_α_7_α_8_). The active-site cavity, which is located between the two domains, comprises a glutathione binding site (G-site), formed mainly by the N-terminal domain, and a hydrophobic substrate-binding site (H-site), which is formed by the C-terminal domain. We found the structure to be very similar to the published binary complex of *Pt*GSTF8 with GSH (PDB 5F07)^39^ at an overall root-mean-square deviation (RMSD) (Cα) of 0.16 Å (Fig. 2a,b). However, presumably to accommodate (−)-catechin (**12**), the active site is substantially enlarged by a shift of the α4′′ and α4′′′ helices by up to 1.71 Å (Fig. 2a,b) and an upwards movement of the side chain of phenylalanine F112 (Fig. 2d and Extended Data Fig. 7c). Within the H-site, the substrate analogue (**12**) interacts through hydrogen bonds to N108 and the backbone of V12, a *π*–*π* interaction with F112, water bridges to R16 and Y175, and hydrophobic interactions with V12 and V115 (Fig. 2c). Interestingly, the amino acid composition of the substrate-binding H-site and the G-site, which binds to GSH through hydrogen bonds and salt bridges (Extended Data Fig. 7b)^39^, is strikingly uniform amongst arGSTFs (Fig. 2e,f), making the existence of similar substrate–enzyme interactions in other arGSTFs plausible.

To shed light on the interactions of the enzyme with the natural substrates, we docked flavan-3,3,4-triol (**4**) and flavan-3-on-4-ol (**7**) into the active pocket of the crystal structure of *Pt*GSTF8 obtained with (−)-catechin (**12**) (Extended Data Fig. 7d,e). The best binding poses for both substrates had a highly similar conformation to (−)-catechin (**12**), with an RMSD of matched atoms of 0.51 Å for flavan-3,3,4-triol (**4**) and 0.54 Å for flavan-3-on-4-ol (**7**). The docking analyses revealed that the positioning of the GSH-thiolate in relation to C-2 and O-3 of the docked substrates could enable an acid–base mechanism of dehydration or tautomerization, in which the GSH cofactor acts as a proton-relay system (Fig. 2g and Extended Data Figs. 6a and 7f).

### Structural basis of arGST activity

**Fig. 3:**
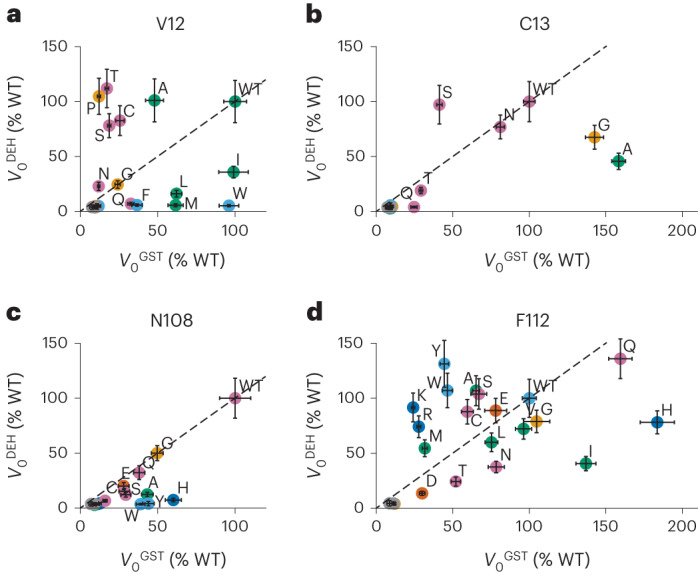
Mutational study of *Pt*GSTF8. **a**–**d**, Initial reaction velocities of the dehydratase activity (*V*_0_^DEH^) towards ultrafiltered flavan-3,3,4-triol (**4**) and GSH-transferase activity (*V*_0_^GST^) towards CDNB (**5**) of *Pt*GSTF8 variants at V12 (**a**), C13 (**b**), N108 (**c**) and F112 (**d**) measured in colorimetric 96-well-plate assays. Data points are coloured according to amino acid properties: green, hydrophobic aliphatic; light blue, hydrophobic aromatic; purple, polar; dark blue, basic; red, acidic; yellow, special. The dashed bisector line marks where an equivalent effect on both tested enzymatic activities would be expected. Data are mean values ± s.d. of three independent replicates. Individual data points can be found in Supplementary Table 7. WT, wild type. Source data

Guided by the structural information derived from the ternary complex of GSH, (−)-catechin (**12**) and *Pt*GSTF8, we selected a set of amino acids in *Pt*GSTF8 for mutagenesis studies as a probe for the positions’ importance in catalysis. Amino acids V12 (putative oxyanion hole), F112 (active site architecture), N108 (flavonoid binding) and C13 (GSH stabilization) were fully randomized by site saturation mutagenesis (NNK codons), and the created *Pt*GSTF8 variants were tested for conversion of flavan-3,3,4-triol (**4**) to cyanidin (**8**), and for their GSH-transferase activity towards CDNB (**5**) as a control of catalytic competence. For all four tested residues, we found that depending on the substitution introduced, the two enzymatic activities were impacted to different extents (Fig. 3). For positions V12 and F112, many of the tested variants exhibited wild-type-level dehydratase activity (Fig. 3a,d). Unsurprisingly, we found that the putative oxyanion hole, formed by the NH backbone of V12, could be provided by other amino acid residues. In addition, the observed side-chain flexibility of V12 and F112 indicated that these residues are mainly involved in the overall shaping of the active site and help to position the flavonoid substrate in accordance with the presented structural evidence. Interestingly, the observation that other amino acids than V12 and F112 can principally support flavonoid binding is also reflected in the more relaxed amino acid conservation in native arGSTFs at these two positions (Fig. 2e). Probing position N108, we found that incorporation of any other amino acid resulted in a substantially reduced formation of cyanidin (**8**) suggesting that the hydrogen-bond interaction of the asparagine to the flavonoid substrate is critical for dehydratase activity, while GSH-transferase activity was comparatively less affected for N108 variants comprising an alanine, histidine, tyrosine or tryptophan (Fig. 3c). C13 is optimally placed to form a hydrogen bond with the deprotonated cofactor GSH (Fig. 2g and Extended Data Fig. 7f). In line with this hypothesis, the activity-conferring substitution pattern of C13, which is highly conserved in arGSTs (Fig. 2f), was found to be much less permissive than for V12 and F112 (Fig. 3b). While a substitution with the polar serine at position 13 retained wild-type dehydratase activity, presumably due to the alcohol’s ability to assume cysteine’s function in stabilizing deprotonated GS^−^, the only other *Pt*GSTF8 variants to keep an—albeit reduced—flavan-3,3,4-triol (**4**) dehydration activity contained a glycine, alanine or asparagine mutation (Fig. 3b). These variants equally preserved their GSH-transferase activity, suggesting the importance of additional factors in the modulation of the negative log of GSH’s acid dissociation constant (p*K*_a_), including the ability to optimally arrange the well-described electron-sharing network which generates a positively charged electrostatic field in the G-site of GSTs^40,41^.

To assess the general relevance of the investigated positions in the context of alternative arGSTs, we introduced three selected substitutions (A/V12M, C13S and N108H) into the two well-studied arGSTs *Ph*AN9 and *Vv*GST4. In this experiment we observed similar trends on dehydratase and GST-transferase activity (Extended Data Fig. 7g–i) as observed for *Pt*GSTF8, suggesting that the proposed catalytic mechanism and active site architecture are conserved between arGSTs from different plant origins.

**Extended Data Fig. 8:**
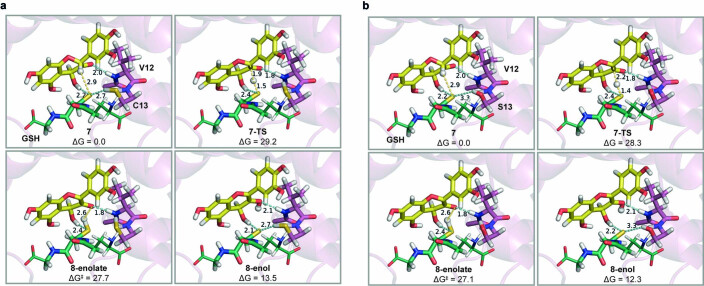
QM/MM calculations of the *Pt*GSTF8 wild-type and C13S reaction pathways. **a,b**, Representation of the stationary points calculated with ONIOM(ωB97X-D/6-311 + G(2d,p):ff14SB,gaff2,TIP3P)//ONIOM(B3LYP/6-31 G(d):ff14SB,gaff2,TIP3P) for the *Pt*GSTF8-catalysed deprotonation-protonation reaction: flavan-3-on-4-ol (**7**), transition state for deprotonation (**7-TS**), deprotonation product (**8-enolate**) and cyanidin (**8-B4**) bound to the wild-type enzyme (**a**) and the C13S variant (**b**). The calculated activation energies (ΔG^‡^ in kcal mol^-1^) for the abstraction the C2 proton of **7** to produce the cyanidin enolate (**8-enolate)** were found to be almost identical for the wild-type and the C13S variant, in agreement with their similar dehydratase activity measured experimentally. Following the rate determining formation of **8-enolate**, this intermediate shows only marginal stabilization over the corresponding transition state and evolves to **8-B4** in a barrierless step. The overall reaction is endergonic for both variants. However, unbinding of the product was calculated to be ~7 kcal mol^-1^ more favorable than the reactant, ensuring catalytic turnover. Substrate is shown in yellow, GS^−^ in green and residues C/S13, V12 and the backbone of A11 in magenta. The protein backbone is shown as a semi-transparent magenta ribbon. Atoms represented in thick sticks were included in the high layer (QM level), while the rest were treated at the MM level. Water molecules are omitted for clarity.

To further probe the catalytic mechanism of arGSTs, we conducted quantum mechanics/molecular mechanics (QM/MM) calculations based on the ternary complex of GSH, (−)-catechin (**12**) and *Pt*GSTF8. Extensive experimental and computational studies have shown that the binding of GSH to GSTs decreases the p*K*_a_ of the GSH thiol group from around 9 to about 6 in the active site through hydrogen bonding and salt bridges and through the positive electrostatic potential of the G-site^41,42^. Consequently, the GSH deprotonation step was considered non-rate-determining and thermodynamically favoured, leading us to use the GSH-thiolate as the starting point to compute the reaction mechanism for the conversion of flav-3-on-4-ol (**7**) into cyanidin (**8-B4**) in the wild-type enzyme and the C13S variant (Extended Data Fig. 8). The analysis of the optimized structures of the Michaelis–Menten complex indicate that the polar residues at position 13 (Cys or Ser) stabilize the deprotonated GS^−^, which in turn acts as the catalytic Brønsted base and abstracts the proton at position C2 of substrate **7** (Extended Data Fig. 8 and Supplementary Figs. 1 and Fig. 2). Notably, the hydroxyl group at the C4 position of the tetrahydropyran ring of substrate **7** also stabilizes the GSH-thiolate through an additional hydrogen bond (Extended Data Fig. 8). As hypothesized from the available structural data, the NH backbone of V12 serves as an oxyanion hole for the enolate formed upon deprotonation.

**Extended Data Fig. 9:**
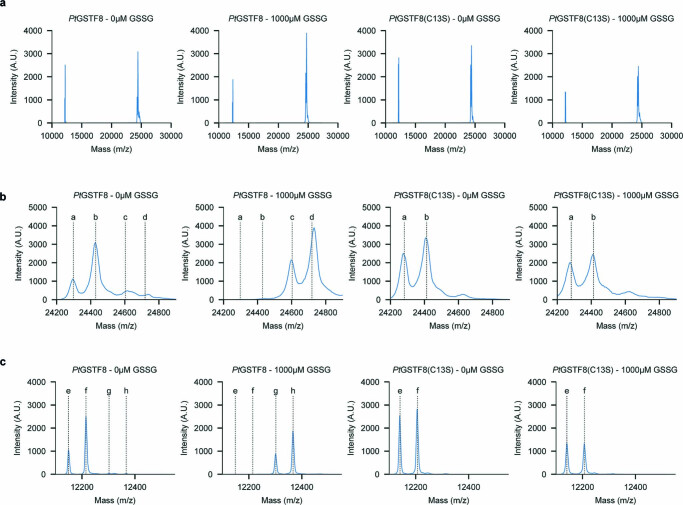
MALDI analysis of oxidation of *Pt*GSTF8 variants. **a**, Full MALDI mass spectrum of *Pt*GSTF8 wild-type and C13S variant after oxidation with 0 or 1 mM GSSG. **b**, Zoom into the area of the single charged ions in the MALDI mass spectra. Expected m/z for [E-Met+H]^+^ (a), [E + H]^+^ (b), [E-Met+GSH + H]^+^ (c), and [E + GSH + H]^+^ (d) are indicated. **c**, Zoom into the area of the double charged ions in the MALDI mass spectra. Expected m/z for [E-Met+2H]^2+^ (e), [E + 2H]^2+^ (f), [E-Met+GSH + 2H]^2+^ (g), and [E + GSH + 2H]^2+^ (h) are indicated. Source data

Informed by the structural data of *Pt*GSTF8, the accompanying mutagenesis experiments and the QM/MM studies, we investigated the impact of a disulfide bond between C13 and GSH on dehydratase activity. To this end, we attempted to form the mixed disulfide bond with purified apo-*Pt*GSTF8 and its C13S variant using oxidized glutathione (GSSG). The expected increase of *m*/*z* was only observed in the matrix-assisted laser desorption ionization (MALDI) spectra of the wild-type enzyme, in line with an *S*-glutathionylation at C13 (Extended Data Fig. 9). When assayed with ultrafiltered flavan-3,3,4-triol (**4**), oxidation of C13 in the wild-type enzyme resulted in a drastically lower formation of cyanidin (**8**), while we observed no effect for the C13S variant (Fig. 2h), giving further evidence of the importance of free GSH for catalysis.

### Anthocyanin production in yeast

**Fig. 4:**
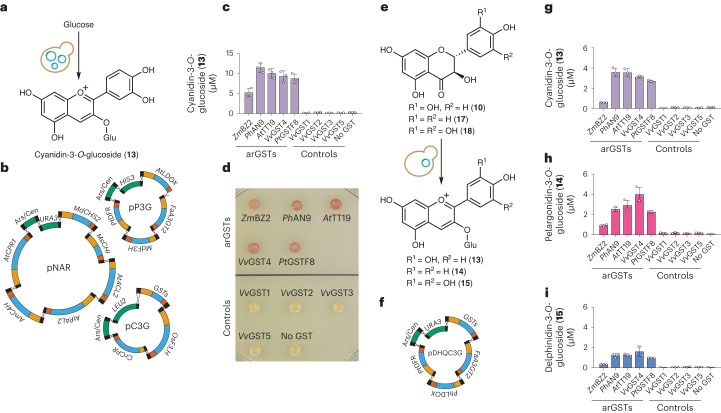
Anthocyanin production in engineered *S. cerevisiae*. **a**, Biosynthesis of cyanidin-3-*O*-glucoside (**13**) from glucose in yeast. **b**, Stepwise assembly of cyanidin-3-*O*-glucoside (**13**) pathway (Supplementary Table 1) on three plasmids using transcription-associated recombination. Blue, open reading frames; orange, promoters; red, terminators; black, linkers; green, plasmid backbone elements. **c**, Cyanidin-3-*O*-glucoside (**13**) product titres in liquid cultures of engineered *S. cerevisiae* strains grown on glucose and expressing the anthocyanin biosynthetic pathway with representative anthocyanin-related and control GSTs. **d**, Colour formation on SC-ULH agar plates of engineered *S. cerevisiae* strains expressing the anthocyanin biosynthetic pathway with representative anthocyanin-related and control GSTs. **e**, Biosynthesis of cyanidin-3-*O*-glucoside (**13**), pelargonidin-3-*O*-glucoside (**14**) and delphinidin-3-*O*-glucoside (**15**) from the respective dihydroflavonols (dihydroquercetin (**10**), dihydrokaempferol (**17**) and dihydromyricetin (**18**)). **f**, Assembly of the partial anthocyanin (**1**) pathway on a single plasmid using transcription-associated recombination. Blue, open reading frames; orange, promoters; red, terminators; black, linkers; green, plasmid backbone elements. **g**–**i**, Cyanidin-3-*O*-glucoside (**13**) (**g**), pelargonidin-3-*O*-glucoside (**14**) (**h**) and delphinidin-3-*O*-glucoside (**15**) (**i**) product titres of engineered *S. cerevisiae* strains expressing the partial anthocyanin biosynthetic pathway with representative anthocyanin-related and control GSTs. The cultures were grown in media containing 300 µM of the respective dihydroflavonol. Data in **c**, **g**, **h** and **i** are mean values ± s.d. of three independent replicates. *A3GT* encodes a anthocyanidin-3-*O*-glycosyl transferase; *CHI* encodes a chalcone isomerase; *CHS* encodes a chalcone synthase; *C4H* encodes a cinnamate-4-hydroxylase; *CPR* encodes a cytochrome P450 reductase; *DFR* encodes a dihydroflavonol-4-reductase; *F3H* encodes a flavanone-3-hydroxylase; *F3*′*H* encodes a flavonoid-3′-hydroxylase; *GST* encodes a glutathione transferase; *LDOX* encodes a leucoanthocyanidin dioxygenase; *PAL* encodes a phenylalanine ammonia lyase; *4CL* encodes a 4-coumarate-CoA ligase. Source data

To highlight the practical relevance of our pathway elucidation, we set out to show that the catalytic activity of arGSTs is also required for anthocyanin biosynthesis in vivo in a eukaryotic cell and thus is key for production of anthocyanins (**1**) in microbial cell factories. To this end, we engineered *S. cerevisiae* to produce cyanidin-3-*O*-glucoside (**13**), the first stable compound in the anthocyanin pathway, using a three-plasmid strategy (Fig. 4a,b). For the known anthocyanin pathway, we selected genes encoding enzymes previously found to be efficient in *S. cerevisiae*^6^ and combined them with a set of nine representative plant-derived GSTs. In this in vivo system, all tested arGSTs boosted the titre of the end-product cyanidin-3-*O*-glucoside (**13**) by a factor of up to 36.5, while the control GSTs unrelated to anthocyanin biosynthesis were comparable to the control without GST (Fig. 4c,d). To further explore the substrate scope of arGSTs, additional yeast systems were engineered using a one-plasmid strategy containing genes encoding dihydroflavonol-4-reductase (DFR), LDOX, a range of arGST and non-arGSTs (as negative controls) and anthocyanidin-3-*O*-glucosyl transferase (A3GT) (Fig. 4e,f). Feeding of selected dihydroflavonols (that is, dihydroquercetin (**10**), dihydrokaempferol (**17**) and dihydromyricetin (**18**)) led to the formation of the corresponding coloured and stable anthocyanidin-3-*O*-glucosides, namely cyanidin-3-*O*-glucoside (**13**), pelargonidin-3-*O*-glucoside (**14**) and delphinidin-3-*O*-glucoside (**15**), underscoring the arGSTs’ biosynthetic relevance in the formation of additional natural pigments. As observed in the case of the whole biosynthetic pathway, yeast strains engineered to incorporate GSTs unrelated to anthocyanin biosynthesis did not produce any of the anthocyanidin-3-*O*-glucosides (Fig. 4g–i).

## Discussion

This work demonstrates that arGSTs have a catalytic role in the biosynthetic pathway to anthocyanins (**1**). Based on the here-presented biochemical, structural and computational evidence, we propose that arGSTs utilize a GSH-dependent Brønsted base mechanism for conversion of the semi-stable LDOX product flavan-3,3,4-triol (**4**) or flavan-3-on-4-ol (**7**) into flav-2-en-3,4-diol (**8**-B4), the 4-hydration species of cyanidin (**8**) (Extended Data Fig. 6a). Similar mechanisms in which the GSH-thiolate acts as a Brønsted base catalyst have been suggested for the well-studied isomerization of ketosteroids by GST A3-3^43^ (Extended Data Fig. 6b), and for the tautomerization of (*R*)-2-hydroxymenthofuran by GST A1-1^44^ (Extended Data Fig. 6c).

The main phenotype of loss-of-function mutations in arGSTs is the decreased content of anthocyanins (**1**)^19–28^ and proanthocyanidins^27^, which can be well explained by an incomplete biosynthesis due to the absence of an essential enzyme. Furthermore, hitherto puzzling observations, such as the halo formation in complementation experiments carried out with arGST-deficient maize and Arabidopsis plants^28^, and the requirement for arGSTs in proanthocyanidin formation in planta^27^, can be rationalized with the catalytic role of arGSTs in producing anthocyanidin precursors. In Arabidopsis, arGST loss-of-function mutations were additionally linked with an increased content of flavonols^19,45^, most probably due to a second oxidation of accumulating flavan-3,3,4-triol (**4**), which is catalysed by *At*LDOX in vitro^11–13^. Notably, anthocyanin biosynthesis is believed to occur on the surface of the endoplasmatic reticulum in membrane-associated enzyme complexes, so-called metabolons^46^. The here-proposed catalytic involvement of arGSTs in the biosynthesis would profit from such association with metabolons and is indirectly observed through the enzymes’ localization to membranes in planta^30^. Further evidence for this hypothesis comes from recent yeast two-hybrid studies performed with enzymes from sweet peony, where the arGST *Ps*GSTF3 was found to interact with *Ps*DFR, the dihydroflavonol reductase catalysing the formation of 3,4-*cis*-leucocyanidin (**6**)^47^. Considering our results, the currently accepted role of arGSTs in transport of anthocyanins (**1**) in planta will have to be reconsidered as less plausible.

The elucidation of arGSTs’ role as biosynthetic enzymes in the anthocyanin pathway has a direct implication for production of these important pigments in microbial cell factories, as highlighted by a 36.5-fold improvement of the cyanidin-3-*O*-glucoside (**13**) titre through the coexpression of arGSTs in our prototype *S. cerevisiae* production strains. Accordingly, we show that the inclusion of arGSTs in our production strains substantially boosts formation of pelargonidin-3-*O*-glucoside (**14**) and delphinidin-3-*O*-glucoside (**15**). In conclusion, the present work provides crucial biosynthetic insights for a more sustainable production of designer anthocyanins (**1**) and is anticipated to drive the construction of powerful, industrial anthocyanin-producing strains, further fuelling the ongoing biologization of the food and nutraceutical sectors.

## Methods

### Materials

Chemicals were ordered from Sigma, ABCR, Carl Roth, Lucerna Chem, Plantmetachem or Formedium, and were used without further purification. Oligonucleotides were ordered from Microsynth. Q5 High-Fidelity polymerase, T4 DNA ligase and restriction enzymes were ordered from New England BioLabs. Clonal genes and gene fragments were ordered from Twist Bioscience. An Excel table containing additional information (company name, catalogue number) of all employed commercial reagents is available as Supplementary Data.

### Synthesis of 3,4-*cis*-leucocyanidin (6)

Dihydroquercetin (**10**) at 5 mg ml^−1^ was reduced with NaBH_4_ at 5 mg ml^−1^ in ethanol to form 3,4-*trans*-leucocyanidin (**14**) under vigorous stirring for 90 min at 23 °C. Next, 10 vol of 1% acetic acid was added, and the solution was stirred vigorously for 3 h at 40 °C to isomerize 3,4-*trans*-leucocyanidin (**14**) to 3,4-*cis*-leucocyanidin (**6**). After adding 3-morpholino-2-hydroxypropanesulfonic acid (MOPSO) to a final concentration of 10 mM and setting the pH to 6.2 using 5 N NaOH, the product was extracted four times with 1 vol of ethyl acetate. The extract was rotary evaporated at 30 °C to a volume of 2 ml, acidified to pH 4 with formic acid, and 200 μl aliquots were purified using preparative HPLC (Agilent Technologies, 1260 Infinity) over a Luna 5 µm phenyl-hexyl 100 Å (250 × 10 mm) column and a 15 min linear gradient from 0% acetonitrile in H_2_O to 16% acetonitrile in H_2_O at 4 ml min^−1^ flow. After pooling the fractions containing pure 3,4-*cis*-leucocyanidin (**6**), MOPSO was added to a final concentration of 10 mM, the pH was set to 6.2 using 5 N NaOH and the pooled fractions were rotary-evaporated to a concentration of 7 mM of 3,4-*cis*-leucocyanidin (**6**), quantified by HPLC-UV (280 nm) with an external calibration curve with 3,4-*trans*-leucocyanidin (**14**). Finally, 3,4-*cis*-leucocyanidin (**6**) was aliquoted, flash-frozen in liquid nitrogen and stored at −80 °C until further use.

### Plasmids, subcloning and creation of NNK libraries

Genes, codon optimized for expression in *S. cerevisiae*, were obtained from Twist Bioscience as DNA strands or subcloned into pET-28b(+) or pTwist Amp MC (Supplementary Tables 2 and 3). Coding sequences of all genes used can be found in Supplementary Table 6.

Basic parts of yeast expression cassettes, which were flanked by linkers for assembly of multi-expression plasmid by homologous recombination (adapted from a method described by Zhao et al.^48^), were obtained from Twist Bioscience in a pTwist Amp backbone (Supplementary Table 2). Additional expression cassettes were constructed by restriction-enzyme-based subcloning according to the manufacturer’s instructions, performed in *E. coli* NEB Turbo (New England BioLabs) as outlined in Supplementary Table 2.

Point mutations and NNK site saturation libraries of *PtGSTF8* were constructed by overlap extension PCR as previously reported^49^ using primers T7 fw, T7 term and:

*PtGSTF8*(V12X): V12C13_rv and V12X_fw, *PtGSTF8*(C13X): V12C13_rv and C13X_fw, *PtGSTF8*(C13S): V12C13_rv and C13S_fw, *PtGSTF8*(N108X): N108_rv, N108X_fw, *PtGSTF8*(F112X): F112_rv, F112X_fw (Supplementary Table 4) using pANT35 as template.

*PhAN9*(A12M): *PhAN9*_A12M_fw, *PhAN9*_A12M_rv, *PhAN9*(C13S): *PhAN9*_C13S_fw, *PhAN9*_C13S_rv, *PhAN9*(N108H): *PhAN9*_N108H_fw, *PhAN9*_N108H_rv (Supplementary Table 4) using pANT15 as template.

*VvGST4*(A12M): *VvGST4*_A12M_fw, *VvGST4*_A12M_rv, *VvGST4*(C13S): *VvGST4_*C13S_fw, *VvGST4*_C13S_rv, *VvGST4*(N108H): *VvGST4*_N108H_fw, *VvGST4*_N108H_rv (Supplementary Table 4) using pANT20 as template.

### Production and purification of *At*LDOX with C-terminal Strep-tag

For expression of *At*LDOX with a C-terminal Strep-tag, *E. coli* BL21(DE3) containing plasmid pANT43 was inoculated from an overnight preculture at a 1:100 ratio in four 2 l baffled Erlenmeyer flasks filled with 400 ml TB medium containing 50 μg ml^−1^ kanamycin sulfate and incubated at 37 °C, 140 rpm using a 5 cm shaking diameter for 2.75 h. Enzyme expression was induced by the addition of 100 μM isopropyl β-d-1-thiogalactopyranoside, the cultures were incubated for another 3.5 h at 30 °C, 140 rpm using a 5 cm shaking diameter, and the final OD_600_ (optical density at 600 nm) was determined using a CO8000 cell density meter (WPA biowave). Cells were harvested by centrifugation at 4,400*g* at 4 °C for 15 min and the medium was discarded. Cell pellets were resuspended in LDOX lysis buffer (20 mM potassium phosphate, 200 mM NaCl, pH 7.4, 0.01 mg ml^−1^ DNAseI) to an OD_600_ of 140 and lysed by two rounds of sonication for 2 min at 50% amplitude with 2 s intervals using a Sonopuls (Bandelin) sonicator. Cell debris was removed by centrifugation at 4,400*g* at 4 °C for 30 min. The clarified cell lysate was either directly submitted to further protein purification steps or flash-frozen in liquid nitrogen after addition of 10% glycerol and stored at −80 °C for use in biocatalytic reactions.

For purification, a 5 ml StrepTrap HP (GE Healthcare) was equilibrated with at least five column volumes of lysis buffer. After filtering the clarified cell lysate through a 0.45 μm filter, it was loaded onto the column at a flow rate of 3 ml min^−1^. The column was washed with 15 column volumes of PPS buffer (20 mM potassium phosphate, 200 mM NaCl, pH 7.4) at a flow rate of 5 ml min^−1^. Next, the protein was eluted using LDOX elution buffer (20 mM potassium phosphate, 200 mM NaCl, 2.5 mM *d*-desthiobiotin, pH 7.4) and the fractions were combined according to the recorded absorption 280 nm (*A*_280nm_). Finally, the buffer was exchanged to PPS buffer using three serially connected 5 mL HiTrap Desalting Columns (GE Healthcare). The concentration of purified *At*LDOX was measured with a NanoDrop spectrophotometer (Thermo Fisher Scientific) at *A*_280nm_ using an extinction coefficient estimated with ProtParam^50^. After supplementation with 10% glycerol, aliquots of purified protein were flash-frozen in liquid nitrogen and stored at −80 °C until further use.

### Small-scale production of GSTs for reactions with clarified cell lysates

For small-scale expression of GST variants, *E. coli* containing pET-28b(+) based plasmids (pANT13-16, pANT18, pANT20-21, pANT35, pANT95, pJK1-3, pJK7-9 or site saturation variants of pANT35) were inoculated from an overnight preculture at a ratio of 1:100 in 96-deep-well plates equipped with a CR1296 sandwich cover (EnzyScreen) in 0.5 ml TB medium containing 50 μg ml^−1^ kanamycin sulfate per well and incubated at 37 °C, 300 rpm using a 5 cm shaking diameter for 2.5 h. Enzyme expression was induced by adding 100 μM isopropyl β-d-1-thiogalactopyranoside, and the cultures were incubated for another 20.5 h at 20 °C, 300 rpm, 5 cm shaking diameter. Cells were harvested by centrifugation at 4,400*g* at 4 °C for 15 min, and the medium was discarded. Cell pellets were resuspended in 0.3 ml small-scale lysis buffer (20 mM potassium phosphate, 200 mM NaCl, pH 7.4, 1 mg ml^−1^ lysozyme, 0.75 mg ml^−1^ polymyxin B, 0.01 mg ml^−1^ DNase I) per well and incubated at 30 °C, 300 rpm, 5 cm shaking diameter for 30 min. Cell debris was removed by centrifugation at 4,400*g* at 4 °C for 30 min, and the clarified cell lysate was directly used for biocatalytic reactions. GST expression levels were analysed via SDS–PAGE. Gel pictures were captured using UV Lab (4.1.0) software.

### Large-scale production and purification of GSTs

For large-scale expression of *Pt*GSTF8 and *Pt*GSTF8(C13S), *E. coli* containing plasmid pANT35 or pANT62 was inoculated from an overnight culture at a ratio of 1:100 in a 2 l baffled Erlenmeyer flask filled with 400 ml Zym-5052 medium^51^ containing 50 μg ml^−1^ kanamycin sulfate per flask, incubated at 20 °C, 140 rpm, 5 cm shaking diameter for 24 h, and the final OD_600_ was determined using a CO8000 cell density meter (WPA biowave). Cells were harvested by centrifugation at 4,400*g* at 4 °C for 15 min and the medium was discarded. Cell pellets were resuspended in reducing lysis buffer (20 mM potassium phosphate, 200 mM NaCl, pH 7.4, 10 mM DTT, 0.01 mg ml^−1^ DNase I) to an OD_600_ of 140 and lysed by two rounds of sonication for 2 min at 50% amplitude with 2 s intervals using a Sonopuls (Bandelin) sonicator. Cell debris was removed by centrifugation at 4400*g* at 4 °C for 30 min and the clarified cell lysate was used for protein purification.

For purification, a 5 ml GSTrap FF (GE Healthcare) was equilibrated with at least five column volumes of reducing lysis buffer. After filtering the clarified cell lysate through a 0.45 μm filter, the crude protein solution was loaded onto the column at a flow rate of 3 ml min^−1^. The disulfide bond between *Pt*GSTF8 residue C13 and GSH was reduced on-column with 20 column volumes of rPPS buffer (20 mM potassium phosphate, 200 mM NaCl, 10 mM DTT, pH 7.4), at a flow rate of 5 ml min^−1^ and the column was then washed with 40 column volumes of PPS buffer at a flow rate of 5 ml min^−1^ to remove free GSH. Next, the apo-enzyme was eluted using GST elution buffer (50 mM Tris, 10 mM methylglutathione, pH 8) before relevant fractions were combined according to their *A*_280nm_. Finally, the buffer was exchanged to PPS buffer, the concentration measured and the apo-enzyme flash-frozen as described for *At*LDOX.

### Oxidation of disulfide bonds of *Pt*GSTF8

Disulfide bonds between C13 of purified apo-*Pt*GSTF8 variants and GSH were formed by incubating 20 μM *Pt*GSTF8 with 0 mM or 1 mM GSSG in the presence of 200 μM GSH in 10 mM MOPSO, pH 6.45 for 2 h at 30 °C, 800 rpm using a 3 mm shaking diameter. Then, 10 μl aliquots of these reactions were directly added to 90 μl ultrafiltered flavan-3,3,4-triol (**4**) to initiate the second step of the two-step in vitro assays with 3,4-*cis*-leucocyanidin (**6**) as described below.

For analysis of disulfide bond formation by MALDI, the samples were desalted and concentrated using C18 Ziptip pipette tips (Millipore). Next, 1 μl of the samples were mixed with 1 μl of sinapinic acid (Sigma-Aldrich) matrix solution and spotted onto a ground steel MALDI target and allowed to dry. Mass spectra were measured in linear positive mode, using an AutoflexSpeed MALDI mass spectrometer (Bruker Daltonics). The instrument was equipped with Nd:YAG laser, emitting at 355 nm. Data collection was carried out using Compass for flexSeries (1.4).

### In vitro GST assay with CDNB (5)

Glutathione transferase assays with CDNB (**5**) were performed in 50 mM Tris, pH 7.5 with 5% clarified cell lysate, 1 mM CDNB and 1 mM GSH. All components were mixed, and reactions were performed in 100 μl volume in 96-well microtitre plates (PS, F-Bottom, clear, Greiner). Plates were incubated for 15 min in a Spark 20M (Tecan) plate reader at 30 °C and *A*_340nm_ was measured every 26 s with a 5 s shaking interval between measurements at 180 rpm using a 3 mm shaking diameter. Data collection was carried out using the software Sparkcontrol (2.3). Initial reaction velocities were calculated from the slope of the *A*_340nm_ data from 1 min to 3 min using *ε* = 9,600 cm^−1^M^−1^ for the concentration calculation of the conjugation product.

### In vitro assays with 3,4-*cis*-leucocyanidin (6)

Standard reaction conditions for in vitro one-step and two-step biotransformations of 3,4-*cis*-leucocyanidin (**6**) were 100 mM MOPSO, pH 6.2, 20 mM sodium ascorbate, 1 mM α-ketoglutaric acid, 0.4 mM ammonium iron(II) sulfate, 200 μM 3,4-*cis*-leucocyanidin (**6**) and 20 μM GSH. When using clarified cell lysates, each lysate was added to 5% of the reaction volume. When using purified protein, *At*LDOX was used at a concentration of 1 μM and *Pt*GSTF8 variants were used at a concentration of 2 μM. For the experiment shown in Fig. 1d, the GSH cofactor was replaced with the indicated GSH analogues at the concentration noted on the *x*-axis label. For the experiment shown in Extended Data Fig. 3a,b, extractions were carried out at the time points indicated on the *x*-axis label. For the experiment shown in Extended Data Fig. 3h, the concentration of *Pt*GSTF8 was adapted as indicated on the *x*-axis label. For the experiment shown in Extended Data Fig. 3i, the concentration of GSH was adapted as shown on the *x*-axis label.

For one-step reactions, all components were mixed in 96-well microtitre plates in 100 μl volume (PS, F-Bottom, clear, Greiner) and reactions were performed for 30 min in a Spark 20 M (Tecan) plate reader at 30 °C. *A*_580nm_ was measured every 26 s with a 5 s shaking interval between measurements at 180 rpm using a 3 mm shaking diameter.

For two-step reactions, an initial reaction was performed in 90% of the final reaction volume with all components present except for GSH and GST. The reaction was carried out for 15 min in 2 ml microcentrifuge tubes at 30 °C, 1,200 rpm using a 3 mm shaking diameter. Following the first reaction step, the *At*LDOX protein was removed by filtering the reaction through an Amicon Ultra-4 (10 kDa cut-off) (Merck Millipore) and distributing 90 μl aliquots in 96-well microtitre plates (PS, F-Bottom, clear, Greiner). The second reaction was initiated through the addition of the remaining 10% (10 μl) reaction volume containing GSH and GST and the same plate reader method as used for one-step reactions was applied to follow the reaction progress. Initial reaction velocities were calculated from the slope of the *A*_580nm_ data of the first minute using a calibration curve with a commercial cyanidin (**8**) reference compound.

For the Michaelis–Menten kinetics, the first step was adapted to 700 µM 3,4-*cis*-leucocyanidin (**6**) and 2 µM *At*LDOX. The second step after filtering was performed with 0.23 µM *Pt*GSTF8 and varying dilutions of the first step reaction in buffer to cover substrate concentrations from 389 µM to 6.5 µM. The same plate reader method as used for one-step reactions was applied to follow the reaction progress by measuring *A*_580nm_ every 12 s. Exact flavan-3,3,4-triol (**4**) concentration was evaluated by a full conversion control reaction containing 5 µM *Pt*GSTF8 in the second step and quantification of the product cyanidin (**8**) by HPLC–MS. Initial reaction velocities were calculated from the slope of the *A*_580nm_ data of the first minute using a calibration curve with a commercial cyanidin (**8**) reference compound.

For analysis of flavonoids by HPLC–MS, reaction volumes were split after the plate reader incubations, and a 40 μl aliquot was extracted with 80 μl of 75% methanol in H_2_O, 1 mM EDTA, 0.3% hydrochloric acid for quantification of cyanidin (**8**) concentration while a second 40 μL aliquot was extracted with 80 μl of 75% acetonitrile in H_2_O, 1 mM EDTA for quantification of all other flavonoids. After thoroughly mixing the extract by vortexing, the precipitated protein was removed by centrifugation (16,000*g*, 5 min, 4 °C) before the supernatants were analysed by HPLC–MS.

Ultraviolet–visible spectra were captured using a Lambda 465 (PerkinElmer) spectrometer. For ultraviolet–visible measurements, one-step in vitro reactions with the standard reaction conditions as defined above were performed in a total volume of 1.2 ml in 2 ml microcentrifuge tubes incubated at 30 °C for 15 min at 1,200 rpm using a 3 mm shaking diameter.

### ^18^O incorporation into cyanidin (8)

Flavan-3,3,4-triol (**4**) was first formed in a reaction with 100 mM MOPSO, pH 6.2, 20 mM sodium ascorbate, 1 mM α-ketoglutaric acid, 0.4 mM ammonium iron(II) sulfate, 500 μM 3,4-*cis*-leucocyanidin (**6**) and 2.5 μM *At*LDOX, which was incubated at 30 °C for 15 min at 1,200 rpm using a 3 mm shaking diameter. Next, the *At*LDOX protein was removed using an Amicon Ultra-4 device (10 kDa cut-off) (Merck Millipore) and the eluent was diluted with 1.375 vol of the same reaction buffer containing 82% H_2_^18^O and incubated at 30 °C, 1,200 rpm using a 3 mm shaking diameter to achieve ^18^O equilibration. At varying time points (0, 2, 5, 15, 30 min), purified *Pt*GSTF8 (8 μM) and GSH (20 μM) were added to an aliquot of the equilibration reaction and the resulting mixtures were incubated at 30 °C for 30 min at 1200 rpm using a 3 mm shaking diameter before extraction with 2 vol of 75% methanol in H_2_O, 1 mM EDTA, 0.3% hydrochloric acid. The ratio of ^18^O incorporation was measured by HPLC–MS and calculated as the ratio of peak areas of selected ion monitoring of the [M]^+^ ions. The expected ^18^O incorporation ratio for stochastic incorporation of 47.4% was calculated from the H_2_^18^O content before addition of *Pt*GSTF8.

### HPLC–MS analysis of flavonoids

HPLC–MS analyses of samples from in vitro reactions or *S. cerevisiae* cultures were analysed with either an Agilent Technologies 1260 Infinity LC System coupled to a 1260 DAD (G4212B) and a LC/MSD XT (G6135B) for quantification of flavonoids or an Agilent Technologies 1290 Infinity coupled to a 1290 DAD (G1316C) and a 6540 UHD Accurate-Mass Q-TOF LC/MS (G6540B) for recording high-resolution mass spectra. For HPLC–MS analyses, data collection was carried out with OpenLAB CDS ChemStation Edition (C.01.07 SR4), while for data analysis the OpenLAB CDS (2.4 Update_06) was used. For high-resolution HPLC–MS runs, data collection was done via MassHunter Workstation Software (B.08.00) and data analysis relied on MassHunter Workstation Software Qualitative Analysis (10.0). Flavonoids were separated with an InfinityLab Poroshell 120, EC-C18 (2.1 × 50 mm, 2.7 μm, Agilent) column at 30 °C. Solvents used were A: 5% acetonitrile in water, 0.2% formic acid; and B: acetonitrile, 0.2% formic acid with a gradient profile of: 0–3 min 0–40% B; 3–3.5 min 40–95% B; 3.5–4.5 95% B, 4.5–4.6 min 95–0% B; 4.6–6 min 0% B at 0.8 ml min^−1^ flow. Flavonoids were quantified using peak areas of *A*_280nm_, *A*_500nm_ or selective ion monitoring in positive mode with external calibration curves prepared in the same solvent as the samples. Epidihydroquercetin (**9**) was quantified using a calibration curve of dihydroquercetin (**10**) assuming a similar ionization, and its retention time was verified using dihydroquercetin (**10**) epimerized as previously reported^13^.

### Yeast strain construction

A high-efficiency lithium acetate transformation protocol^52^ was used for integration of *MET15* and assembly of multi-expression-plasmids in *S. cerevisiae* strains. The native *MET15* ORF was amplified from genomic DNA of *S. cerevisiae* S288C using primers *MET15*_fw and *MET15*_rv and was integrated into yeast strain BY4741 to restore prototrophy for methionine. Next, the multi-expression plasmids were assembled in yeast using transcription-associated recombination as previously described^53^. The construction of all yeast strains is shown in Supplementary Table 5.

### In vivo anthocyanin (1) production in *S. cerevisiae*

For production of flavonoids, engineered *S. cerevisiae* strains C3G1-C3G10 and DHQC3G1–DHQC3G10 (Supplementary Table 5) were restreaked on SC-ULH plates (1.47 g l^−1^ Synthetic Complete (Kaiser) Drop Out: Leu, His, Ura, 6.7 g l^−1^ yeast nitrogen base (without amino acids), 20 g l^−1^ d-(+)-glucose, 16 g l^−1^ agar) or SC-U plates (SC-ULH, 380 mg l^−1^ leucine, 76 mg l^−1^ histidine), respectively. Colonies were used to inoculate 0.25 ml SC-ULH medium (1.47 g l^−1^ Synthetic Complete (Kaiser) Drop Out: Leu, His, Ura, 6.7 g l^−1^ yeast nitrogen base (without amino acids), 20 g l^−1^ d-(+)-glucose), pH set to 5.8 with hydrochloric acid) or SC-U medium (SC-ULH, 380 mg l^−1^ leucine, 76 mg l^−1^ histidine), respectively, in 96-deep-well plates equipped with a CR1296 sandwich cover (EnzyScreen). The medium to produce flavonoids from dihydroflavonols with the DHQC3G1–DHQC3G10 strains contained 300 µM of the precursor (dihydrokaempferol (**17**), dihydroquercetin (**10**) or dihydromyricetin (**18**)). After incubation for 5 days (C3G strains) or 3 days (DHQC3G strains) at 30 °C, 300 rpm, 5 cm shaking diameter, 0.5 ml methanol containing 0.3% hydrochloric acid was added for extraction of flavonoids. After incubation for 15 min at 30 °C, 1,000 rpm using a 3 mm shaking diameter, cell debris was removed by centrifugation (4,400*g* at 4 °C for 30 min) and the supernatants were analysed by HPLC–MS at two different dilutions (undiluted and four times diluted with 67% methanol, 0.2% hydrochloric acid in H_2_O).

### Multiple sequence alignments and sequence logos

A multiple protein sequence alignment of known arGSTs of the phi class (*Ph*AN9^28^ (O24261), *At*TT19^27^ (Q9FE46), *Vv*GST4^30^ (Q56AY1), *Pf*GST1^54^ (B1B5E6), *Cs*GST3^55^ (I2FHU9), *Dc*GSTF2^56^ (I4DUE3), *Pp*Riant^21^ (M5WTI5), *Lc*GST4^57^ (A0A0U4AVK2), *Fv*RAP^20^ (UPI0002C2E8B9), *Md*GST1^25^ (G3LX80), *Ac*GST1^26^ (A0A2R6R622), *Ib*GSTF4^58^ (A0A8E4DFU3), *Cs*GSTa^59^ (A0A4P8P9K5), *Pc*GSTF12^60^ (A0A6G5X592), *Gt*GST1^24^ (NCBI accession number: BCD52748.1), *Lh*GST^23^ (A0A5B9G8S8), *Ep*Bract1^61^ (NCBI accession number: QTX16320.1), *Gh*GSTF12^22^ (A0A5D2YJA6), *Rs*GST1^62^ (A0A6J0K8F1), *Pt*GSTF8^39^ (NCBI accession number, 5F07_A)—Uniprot accession numbers unless stated otherwise) was constructed using Clustal Omega 1.2.2^63^ with standard settings. Sequence logos were generated from the multiple sequence alignment using the R package ggseqlogo^64^.

### Crystallization of *Pt*GSTF8 in presence of (±)-catechin and GSH

Purified *Pt*GSTF8 in 30 mM Tris, pH 8, 200 mM NaCl, 1 mM EDTA was concentrated to 12.4 mg ml^−1^ using an Amicon Ultra-4 device (10 kDa cut-off) (Merck Millipore) and supplemented with 1 mM GSH and 25 mM (±)-catechin immediately before crystallization. Crystallization was performed using the sitting drop method at 4 °C in an Intelli-Plate 96-3 LVR (Hampton Research). A drop of 150 nl enzyme/cofactor/substrate solution was mixed with 150 nl precipitant (100 mM MES, pH 6.4, 200 mM MgCl_2_, 17.9% PEG200) and equilibrated against 50 µl reservoir solution (100 mM MES, pH 6.4, 200 mM MgCl_2_, 17.9% PEG200). Crystals were observed after 5 days of crystallization and picked after 21 days of total incubation. Finally, 2 µl 30% glycerol (in reservoir solution) was added to the drop before picking the crystal using 0.1 mm litholoops and flash-freezing it in liquid nitrogen.

Diffraction data were collected at the Swiss Light Source (Paul Scherrer Institut, Villigen AG) on beamline X06DA-PXIII at a temperature of 100 K and 0.999995 Å wavelength using the serial synchroton crystallography software suite. The data were integrated by XDS^65^ run through autoproc^66^. A dataset with 1.09 Å resolution was obtained. PDB 5F07 was used in molecular replacement to determine the starting phases for refinement with anisotropic B-factors and hydrogen atoms using MOLREP^67^. Refinement and manual model building were performed with REFMAC^68^ and coot^69^, respectively, using the ccp4i2^70^ interface. The final model contains all protein residues except Met1, as well as GSH and (−)-catechin. The MolProbity^71^ validation of this model gave 97.16% Ramachandran favoured residues and 0% outliers with a Molprobity score of 1.16.

### Analysis of *Pt*GSTF8 crystal structures

*F*_o_ – *F*_c_ composite omit maps were calculated with the CCP4 7.1.018: comit^72^ (v.0.1.0) function using the fast method. Molecular docking was performed with AutoDock Vina 1.2.0^73^ using default settings, except for increasing the exhaustiveness to 32. Figure 2b with colour-coded RMSD between crystal structures was generated using the ColorByRMSD Pymol script (https://pymolwiki.org/index.php/ColorByRMSD, accessed on 16 June 2022). The interactions of ligands to the enzyme were analysed using PLIP^74^ with standard settings. The RMSD of matched atoms between ligands was calculated using LigRMSD^75^ with standard settings.

### QM/MM calculations

Enzyme–substrate complexes were generated through molecular docking using the GOLD software^76^ (ChemScore fitness function) on the crystal structure of PtGSTF8 preserving crystallographic Na^+^ ions and flav-3-on-4-ol coordinates optimized at the B3LYP/6-31 G(d) quantum mechanical level (see below for details). The docking cavity was centred on the sulfur atom of the GSH’s cysteine and allowed to extend in a spherical surrounding region with a 15 Å radius. The number of GA (genetic algorithm) runs was set to 30.

The top-ranking binding pose was then prepared for classical MD relaxation with the AMBER 20^77^ suite using the ff14SB^78^ force field for the protein and gaff2^79^ for GSH and flav-3-on-4-ol. Enzyme–substrate complexes were immersed in a water box with an 8 Å buffer of OPC3^80^ water. A two-stage geometry optimization approach was implemented. The first stage minimizes only the positions of solvent molecules and ions, and the second stage is an unrestrained minimization of all the atoms in the simulation cell. The systems were then heated for 100 ps by incrementing the temperature from 0 to 300 K under a constant pressure of 1 atm and periodic boundary conditions with a timestep of 1 fs. Harmonic restraints of 50.0 kcal mol^−1^ Å^−2^ were applied to the solutes, including the crystallographic ions, to allow solvent relaxation while preserving the maximum fidelity to the docking pose, and the Andersen temperature coupling scheme^81,82^ was used to control and equalize the temperature. The last frame from the MD trajectory was then extracted and excess solvent removed leaving a 6 Å thick water shell around the enzyme–substrate complexes.

Full geometry optimizations, relaxed scans and transition structure searches were carried out with Gaussian 16^83^ with the ONIOM^84^ QM/MM method using the B3LYP hybrid functional^85^ and 6-31 G(d) basis set with ultrafine integration grids for the QM part and the ff14SB^78^, gaff2^79^ and TIP3P^86^ force fields for the MM part (protein, substrate and water, respectively) with electrostatic embedding. Only water molecules were kept frozen, allowing the enzyme to adapt along the reaction path. The definition of the QM and MM layers is depicted in Extended Data Fig. 8. The possibility of different conformations was considered for all structures. All stationary points were characterized by a frequency analysis performed at the same level used in the geometry optimizations from which thermal corrections were obtained at 298.15 K. Single-point energies were calculated on the optimized geometries using the ωB97X-D hybrid functional^87^ and the 6-311 + G(2d,p) basis set with ultrafine integration grids for the QM part and the ff14SB, gaff2 and TIP3P force fields for the MM part (protein, substrate and water, respectively) with electrostatic embedding. The lowest-energy conformer for each calculated stationary (available in PDB format as Supplementary Figs. 1 and 2) was considered in the discussion; computed geometries and energies can be accessed through the Zenodo repository (10.5281/zenodo.8069429). ONIOM energies, entropies, enthalpies, Gibbs free energies and lowest frequencies of the calculated structures are summarized in Supplementary Table 8.

### Reporting summary

Further information on research design is available in the Nature Portfolio Reporting Summary linked to this article.

### Supplementary information

Supplementary Information Supplementary Tables 1–8 and Figs. 1 and 2.

Reporting Summary

Supplementary Data List of commercial reagents including supplier and catalogue number.

Supplementary Data Source data for Supplementary Fig. 1.

Supplementary Data Source data for Supplementary Fig. 2.

### Source data

Source Data Fig. 1 Source data for Fig. 1.

Source Data Fig. 2 Source data for Fig. 2.

Source Data Fig. 3 Source data for Fig. 3.

Source Data Fig. 4 Source data for Fig. 4.

Source Data Extended Data Fig. 2 Source data for Extended Data Fig. 2.

Source Data Extended Data Fig. 3 Source data for Extended Data Fig. 3.

Source Data Extended Data Fig. 5 Source data for Extended Data Fig. 5.

Source Data Extended Data Fig. 7 Source data for Extended Data Fig. 7.

Source Data Extended Data Fig. 9 Source data for Extended Data Fig. 9.

## Figures and Tables

**Extended Data Table 1:**
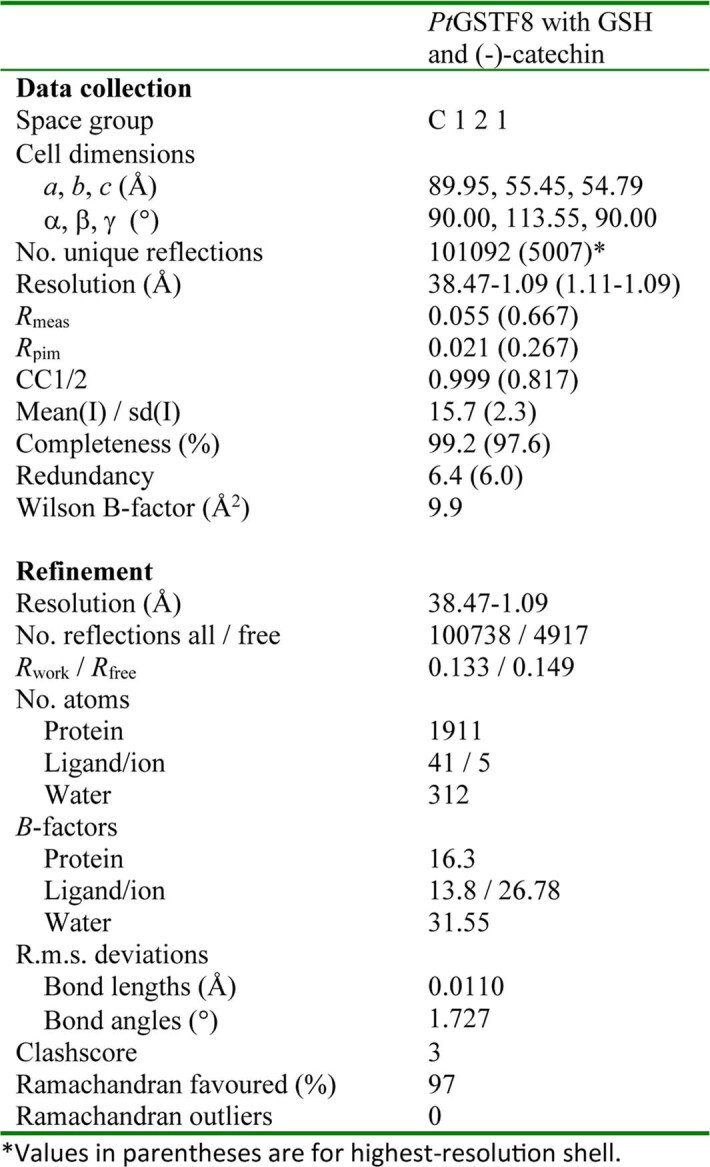
X-ray crystallographic data collection and refinement statistics

## Data Availability

Nucleotide sequences of codon-optimized genes can be found in Supplementary Table 6. The diffraction images were deposited to Integrated Resource for Reproducibility in Macromolecular Crystallography^88^ (http://proteindiffraction.org/) and can be accessed via PDB 8AGQ. Crystallographic coordinates of the ternary complex of *Pt*GSTF8 have been deposited in the PDB as 8AGQ. The binary *Pt*GSTF8 crystal structure used in molecular replacement experiments can be accessed via PDB 5F07. Computed geometries and energies can be accessed through the Zenodo repository (10.5281/zenodo.8069429). Source data are provided with this paper.
