# Supplementary material for: The catalytic role of glutathione transferases in heterologous anthocyanin biosynthesis
Source: Nat Catal. 2023 Aug 31;6(10):927–38. doi: 10.1038/s41929-023-01018-y (PMC10593608; doi:10.1038/s41929-023-01018-y)
Supplement: Supplementary file 1 — Supplementary Tables 1–8 and Figs. 1 and 2. [file 41929_2023_1018_MOESM1_ESM.pdf]

nature catalysis

Article https://doi.org/10.1038/s41929-023-01018-y

The catalytic role of glutathione transferases in heterologous anthocyanin biosynthesis

In the format provided by the authors and unedited

Table of contents:

S2: Supplementary Table 1: List of all enzymes used in this study

S3-S6: Supplementary Table 2. List of plasmids used in this study.

S7: Supplementary Table 3 List of DNA fragments used in this study.

S8-S9: Supplementary Table 4. List of primers used in this study.

S10-11: Supplementary Table 5. List of yeast strains used in this study.

S12-S21: Supplementary Table 6. Coding sequences of enzymes in this study.

S22-S23: Supplementary Table 7. Individual datapoints for Fig. 3.

S24: Supplementary Figure 1. Minimum free energy pathways calculated

S24: Supplementary Figure 2. Minimum potential energy pathways calculated

S25: Supplementary Table 8. Energies, entropies, and lowest frequencies of the lowest energy calculated

Supplementary Table 1. List of enzymes used in this study

Abb.	Name	Organism	Anthocyanin[1] synthesis
*At*PAL2	Phenylalanine ammonia lyase	*A. thaliana*	l-phenylalanine → cinnamic acid + NH_3
*Am*C4H	Cinnamate-4-hydroxylase	*A. majus*	cinnamic acid + redCPR + O_2 → *p*-coumaric acid + oxCPR + H_2O
*At*CPR1	Cytochrome P450 reductase	*A. thaliana*	oxC4H + NADPH → redC4H + $NADP^+$ + H^+
*Cr*CPR1	Cytochrome P450 reductase	*C. roseus*	oxC4H + NADPH + H^+ → redC4H + $NADP^+$
*At*4CL2	4-coumarate-CoA ligase	*A. thaliana*	*p*-coumaric acid + ATP +CoA → *p*-coumaroyl-CoA + AMP + PP_i
*Md*CHS2	Chalcone synthase	*M. domestica*	*p*-coumaroyl-CoA + 3 malonyl-CoA → naringenin chalcone + 3 CO_2 + 4 CoA
*Ms*CHI	Chalcone isomerase	*M. sativa*	naringenin chalcone → naringenin
*Os*F3'H	Flavonoid-3´-hydroxylase	*O. sativa*	naringenin + redCPR + O_2→ eriodictyol + oxCPR + H_2O
*Md*F3H	Flavanone-3-hydroxylase	*M. domestica*	eriodictyol + 2-oxoglutarate + O_2 → dihydroquercetin + succinate + CO_2
*Pt*DFR	Dihydroflavonol-4-reductase	*P. trichocarpa*	dihydroquercetin + NADPH + H^+ → 3,4-cis-leucocyanidin + $NADP^+$
*At*LDOX	Leucoanthocyanidin dioxygenase	*A. thaliana*	3,4-cis-leucocyanidin + 2-oxoglutarate + O_2 → flavan-3,3,4-triol + succinate + CO_2
*Ph*LDOX	Leucoanthocyanidin dioxygenase	*P. trichocarpa*	3,4-cis-leucocyanidin + 2-oxoglutarate + O_2 → flavan-3,3,4-triol + succinate + CO_2
*Zm*BZ2	Glutathione transferases	*Z. mays*	flavan-3,3,4-triol → cyanidin + H_2O
*Ph*AN9	Glutathione transferases	*P. hybrida*	flavan-3,3,4-triol → cyanidin + H_2O
*At*TT19	Glutathione transferases	*A. thaliana*	flavan-3,3,4-triol → cyanidin + H_2O
*Vv*GST4	Glutathione transferases	*V. vinifera*	flavan-3,3,4-triol → cyanidin + H_2O
*Pt*GSTF8	Glutathione transferases	*P. trichocarpa*	flavan-3,3,4-triol → cyanidin + H_2O
*Vv*GST1	Glutathione transferases	*V. vinifera*	-
*Vv*GST2	Glutathione transferases	*V. vinifera*	-
*Vv*GST3	Glutathione transferases	*V. vinifera*	-
*Vv*GST5	Glutathione transferases	*V. vinifera*	-
*Fa*A3GT2	Anthocyanidin-3-O-glycosyltransferase	*F. ananassa*	cyanidin + UDP-D-glucose → cyanidin-3-O-glucoside + UDP

[1] Molecule names refer to the synthesis of cyanidin-3-O-glucoside.

Supplementary Table 2. List of plasmids used in this study.

Name	Insert	Backbone	Type[1]	Source
pANT1	*AtPAL2*	pTwist Amp MC	GS	Obtained from Twist Bioscience
pANT2	*AmC4H*	pTwist Amp MC	GS	Obtained from Twist Bioscience
pANT3	*ScCPR1*	pTwist Amp MC	GS	Obtained from Twist Bioscience
pANT4	*At4CL2*	pTwist Amp MC	GS	Obtained from Twist Bioscience
pANT5	*MdCHS2*	pTwist Amp MC	GS	Obtained from Twist Bioscience
pANT6	*MsCHI*	pTwist Amp MC	GS	Obtained from Twist Bioscience
pANT8	*MdF3H*	pTwist Amp MC	GS	Obtained from Twist Bioscience
pANT9	*PtDFR*	pTwist Amp MC	GS	Obtained from Twist Bioscience
pANT10	*AtCPR1*_co1	pTwist Amp MC	GS	Obtained from Twist Bioscience
pANT11	Strep-tag-*AtLDOX*	pET-28b(+)	BEX	Obtained from Twist Bioscience
pANT12	Strep-tag-*PhLDOX*	pET-28b(+)	BEX	Obtained from Twist Bioscience
pANT13	*FaA3GT2*	pTwist Amp MC	GS	Obtained from Twist Bioscience
pANT14	*ZmBZ2*	pET-28b(+)	BEX	Obtained from Twist Bioscience
pANT15	*PhAN9*	pET-28b(+)	BEX	Obtained from Twist Bioscience
pANT16	*AtTT19*	pET-28b(+)	BEX	Obtained from Twist Bioscience
pANT17	*VvGST1*	pET-28b(+)	BEX	Obtained from Twist Bioscience
pANT18	*VvGST2*	pET-28b(+)	BEX	Obtained from Twist Bioscience
pANT19	*VvGST3*	pET-28b(+)	BEX	Obtained from Twist Bioscience
pANT20	*VvGST4*	pET-28b(+)	BEX	Obtained from Twist Bioscience
pANT21	*VvGST5*	pET-28b(+)	BEX	Obtained from Twist Bioscience
pANT22	B/P_{GPD1}-*MdCHS2*-T_{TPS1}/C	pTwist Amp	YAS	Subclone insert of pANT5 into backbone of pHR1 using HindIII and SacII
pANT23	B/P_{GPD1}-*AtLDOX*-T_{TPS1}/C	pTwist Amp	YAS	Subclone PCR product obtained with primers ScAtANS_fw and ScAtANS_rv from template pANT11 into pHR1 using HindIII and SacII
pANT24	B/P_{GPD1}-*AtLDOX*-T_{TPS1}/C	pTwist Amp	YAS	Subclone PCR product obtained with primers ScPhANS_fw and ScPhANS_rv from template pANT12 into pHR1 using HindIII and SacII
pANT25	C/P_{PGK1}-*MsCHI*-T_{ADH2}/D	pTwist Amp	YAS	Subclone insert of pANT6 into backbone of pHR2 using HindIII and SacII
pANT26	C/P_{PGK1}-*FaA3GT2*-T_{ADH2}/D	pTwist Amp	YAS	Subclone insert of pANT13 into backbone of pHR2 using HindIII and SacII
pANT27	D/P_{TEF1}-*At4CL2*-T_{ENO2}/E	pTwist Amp	YAS	Subclone insert of pANT4 into backbone of pHR10 using HindIII and SacII
pANT28	D/P_{TEF1}-*MdF3H*-T_{ENO2}/E	pTwist Amp	YAS	Subclone insert of pANT8 into backbone of pHR10 using HindIII and SacII

pANT29	E/P_{PDC1}-*AtPAL2*-T_{FBA1}/F	pTwist Amp	YAS	Subclone insert of pANT1 into backbone of pHR11 using HindIII and SacII
pANT30	E/P_{PDC1}-*PtDFR*-T_{FBA1}/F	pTwist Amp	YAS	Subclone insert of pANT9 into backbone of pHR11 using HindIII and SacII
pANT31	F/P_{TEF2}-*AmC4H*-T_{PGI1}/G	pTwist Amp	YAS	Subclone insert of pANT2 into backbone of pHR12 using HindIII and SacII
pANT33	G/P_{PYK1}-*ScCPR1*-T_{PDC6}/H	pTwist Amp	YAS	Subclone insert of pANT3 into backbone of pHR13 using HindIII and SacII
pANT35	*PtGSTF8*	pET-28b(+)	BEX	Obtained from Twist Bioscience
pANT40	G/P_{PYK1}-*ScCPR1*-T_{ADH1}/H	pTwist Amp	YAS	Subclone PCR product obtained with primers tADH_fw and tADH_rv from *S. cerevisiae* S288C genomic DNA into pANT33 using SacII and SphI
pANT43	*AtLDOX*-Strep-tag	pET-28b(+)	BEX	
pANT46	G/P_{PYK1}-*ScCPR*1-T_{ADH1}/Z	pTwist Amp	YAS	Subclone insert of pANT40 into backbone of pHR7 using SacI and SphI
pANT54	G/P_{PYK1}-*AtCPR1*_co1-T_{ADH1}/Z	pTwist Amp	YAS	Subclone insert of pANT10 into backbone of pHR46 using HindIII and SacII
pANT55	E/P_{PDC1}-*PtDFR*-T_{ADH1}/Z	pTwist Amp	YAS	Subclone insert of pANT30 into backbone of pANT46 using SacI and SacII
pANT56	D/P_{TEF1}-*AtCPR1*_co1-T_{ADH1}/Z	pTwist Amp	YAS	Subclone insert of pANT54 into backbone of pHR10 using HindIII and BamHI
pANT57	B/P_{GPD1}-*ZmBZ2*-T_{TPS1}/C	pTwist Amp	YAS	Subclone PCR product obtained with primers ScZmBZ2_fw and ScZmBZ2_rv from template pANT14 into pHR1 using HindIII and SacII
pANT58	B/P_{GPD1}-*PhAN9*-T_{TPS1}/C	pTwist Amp	YAS	Subclone PCR product obtained with primers ScPhAN9_fw and ScPhAN9_rv from template pANT15 into pHR1 using HindIII and SacII
pANT59	B/P_{GPD1}-*AtTT19*-T_{TPS1}/C	pTwist Amp	YAS	Subclone PCR product obtained with primers ScAtTT19_fw and ScAtTT19_rv from template pANT16 into pHR1 using HindIII and SacII
pANT62	*PtGSTF8*(C13S)	pET-28b(+)	BEX	Subclone overlap extension PCR fragment (see methods section) into pANT35 using NdeI and XhoI
pANT70	B/P_{GPD1}-*VvGST1*-T_{TPS1}/C	pTwist Amp	YAS	Subclone PCR product obtained with primers ScVvGST1_fw and ScVvGST1_rv from template pANT17 into pHR1 using HindIII and SacII
pANT71	B/P_{GPD1}-*VvGST2*-T_{TPS1}/C	pTwist Amp	YAS	Subclone PCR product obtained with primers ScVvGST2_fw and ScVvGST2_rv from template pANT18 into pHR1 using HindIII and SacII
pANT72	B/P_{GPD1}-*VvGST3*-T_{TPS1}/C	pTwist Amp	YAS	Subclone PCR product obtained with primers ScVvGST3_fw and

				ScVvGST3_rv from template pANT19 into pHR1 using HindIII and SacII
pANT73	B/P_{GPD1}-*VvGST4*-T_{TPS1}/C	pTwist Amp	YAS	Subclone PCR product obtained with primers ScVvGST4_fw and ScVvGST4_rv from template pANT20 into pHR1 using HindIII and SacII
pANT74	B/P_{GPD1}-*VvGST5*-T_{TPS1}/C	pTwist Amp	YAS	Subclone PCR product obtained with primers ScVvGST5_fw and ScVvGST5_rv from template pANT21 into pHR1 using HindIII and SacII
pANT75	B/P_{GPD1}-*PtGSTF8*-T_{TPS1}/C	pTwist Amp	YAS	Subclone PCR product obtained with primers ScPtGSTF8_fw and ScPtGSTF8_rv from template pANT35 into pHR1 using HindIII and SacII
pANT78	C/P_{PGK1}-*OsF3'H*-T_{ADH2}/D	pTwist Amp	YAS	Subclone PCR product obtained with primers T7 fw and T7 term from template OsF3'H_gB into pHR2 using HindIII and SacII
pANT80	D/P_{TEF1}-*CrCPR*-T_{ADH1}/Z	pTwist Amp	YAS	Subclone DNA fragment CrCPR into pANT56 using HindIII and SacII
pANT81	G/P_{PYK1}-*AtCPR1*_co2-T_{ADH1}/Z	pTwist Amp	YAS	Subclone DNA fragment AtCPR1_co2 into pHR46 using HindIII and SacII
pANT86	D/P_{TEF1}-*PhLDOX*-T_{ENO2}/E	pTwist Amp	YAS	Subclone DNA fragment PhLDOX into pHR10 using HindIII and SacII
pANT95	*AtGSTF2*	pET-28b(+)	BEX	Obtained from Twist Bioscience
pHLUM	*LEU2*/*URA3*/*HIS3*/*MET17*	pRS313	YEX	Obtained from Addgene
pHR1	B/P_{GPD1}-T_{TPS1}/C	pTwist Amp	YAS	Obtained from Twist Bioscience
pHR2	C/P_{PGK1}-T_{ADH2}/D	pTwist Amp	YAS	Obtained from Twist Bioscience
pHR3	D/T_{ENO2}/E	pTwist Amp	YAS	Obtained from Twist Bioscience
pHR4	E/T_{FBA1}/F	pTwist Amp	YAS	Obtained from Twist Bioscience
pHR5	F/T_{PGI1}/G	pTwist Amp	YAS	Obtained from Twist Bioscience
pHR6	G/T_{PDC6}/H	pTwist Amp	YAS	Obtained from Twist Bioscience
pHR7	H/Z	pTwist Amp	YAS	Obtained from Twist Bioscience
pHR8	Z/A	pTwist Amp	YAS	Obtained from Twist Bioscience
pHR9	A/B	pTwist Amp	YAS	Obtained from Twist Bioscience
pHR10	D/P_{TEF1}-T_{ENO2}/E	pTwist Amp	YAS	Subclone PCR product obtained with primers ANT1 and ANT2 from *S. cerevisiae* S288C genomic DNA into pHR3 using XhoI and HindIII
pHR11	E/P_{PDC1}-T_{FBA1}/F	pTwist Amp	YAS	Subclone PCR product obtained with primers ANT3 and ANT4 from *S. cerevisiae* S288C genomic DNA into pHR4 using XhoI and HindIII
pHR12	F/P_{TEF2}-T_{PGI1}/G	pTwist Amp	YAS	Subclone PCR product obtained with primers ANT5 and ANT6 from *S. cerevisiae* S288C genomic DNA into pHR5 using XhoI and HindIII
pHR13	G/P_{PYK1}-T_{PDC6}/H	pTwist Amp	YAS	Subclone PCR product obtained with primers ANT7 and ANT8 from *S. cerevisiae* S288C genomic DNA into pHR6 using XhoI and HindIII

pHR14	Z/ARS/CEN/A	pTwist Amp	YAS	Subclone PCR product obtained with primers ANT9 and ANT10 from template pHLUM into pHR8 using XhoI and SphI
pHR15	A/*HIS3*/B	pTwist Amp	YAS	Subclone PCR product obtained with primers ANT11 and ANT12 from template pHLUM into pHR9 using XhoI and SphI
pHR16	A/*URA3*/B	pTwist Amp	YAS	Subclone PCR product obtained with primers ANT13 and ANT14 from template pHLUM into pHR9 using XhoI and SphI
pHR17	A/*LEU2*/B	pTwist Amp	YAS	Subclone PCR product obtained with primers ANT15 and ANT16 from template pHLUM into pHR9 using XhoI and SphI
pJK1	*PhAN9*(A12M)	pET-28b(+)	BEX	Subclone overlap extension PCR fragment (see methods section) into pANT35 using NdeI and XhoI
pJK2	*PhAN9*(C13S)	pET-28b(+)	BEX	Subclone overlap extension PCR fragment (see methods section) into pANT35 using NdeI and XhoI
pJK3	*PhAN9*(N108H)	pET-28b(+)	BEX	Subclone overlap extension PCR fragment (see methods section) into pANT35 using NdeI and XhoI
pJK7	*VvGST4*(A12M)	pET-28b(+)	BEX	Subclone overlap extension PCR fragment (see methods section) into pANT35 using NdeI and XhoI
pJK8	*VvGST4*(C13S)	pET-28b(+)	BEX	Subclone overlap extension PCR fragment (see methods section) into pANT35 using NdeI and XhoI
pJK9	*VvGST4*(N108H)	pET-28b(+)	BEX	Subclone overlap extension PCR fragment (see methods section) into pANT35 using NdeI and XhoI
pJK1	*PhAN9*(A12M)	pET-28b(+)	BEX	Subclone overlap extension PCR fragment (see methods section) into pANT35 using NdeI and XhoI
pJK2	*PhAN9*(C13S)	pET-28b(+)	BEX	Subclone overlap extension PCR fragment (see methods section) into pANT35 using NdeI and XhoI
pJK3	*PhAN9*(N108H)	pET-28b(+)	BEX	Subclone overlap extension PCR fragment (see methods section) into pANT35 using NdeI and XhoI

[1] GS: gene synthesis plasmid, BEX: bacterial expression plasmid, YEX: yeast expression plasmid, YAS: yeast assembly plasmid

Supplementary Table 3. List of DNA fragments used in this study.

Name	Content	Source
*AtCPR1*_co2_F1	*AtCPR1*_co2 fragment 1	Obtained from Twist Bioscience
*AtCPR1*_co2_F2	*AtCPR1*_co2 fragment 2	Obtained from Twist Bioscience
*AtCPR1*_co2	*AtCPR1*_co2	Assembly of fragments *AtCPR1*_co2_F1 and *AtCPR1*_co2_F2 using overlap extension PCR with primers T7 fw, T7 term, *AtCPR1*_co2_OL_rv, and *AtCPR1*_co2_OL_fw
*CrCPR*_F1	*CrCPR* fragment 1	Obtained from Twist Bioscience
*CrCPR*_F2	*CrCPR* fragment 2	Obtained from Twist Bioscience
CrCPR	*CrCPR1*	Assembly of fragments *CrCPR*_F1 and *CrCPR*_F2 using overlap extension PCR with primers T7 fw, T7 term, *CrCPR*_OL_rv, and *CrCPR*_OL_fw
OsF3'H	*OsF3'H*	Obtained from Twist Bioscience

Supplementary Table 4. List of primers used in this study.

Name	Sequence
ANT1	AGCTACTCGAGTAGCTTCAAAATGTTTCTACTCC
ANT2	AGCTAAAGCTTTTTGTAATTAAAACTTAGATTAGATTGC
ANT3	AGCTACTCGAGATGCGACTGGGTGAGCATATG
ANT4	AGCTAAAGCTTTTTGATAGATTTGACTGTGTTATTTTGCGTGAG
ANT5	AGCTACTCGAGGGGCCGTATACTTACATATAGTAG
ANT6	AGCTAAAGCTTGTTTAGTTAATTATAGTTCGTTGACCG
ANT7	AGCTACTCGAGAATGCTAGTATTTTGGAGATTAATCTC
ANT8	AGCTAAAGCTTTGTGATGATGTTTTATTTGTTTTGATTG
*AtCPR1*_co2_OL_fw	GGTATCACTTACGAAACAGGTG
*AtCPR1*_co2_OL_rv	CACCTGTTTCGTAAGTGATACC
C_Strep_*AtANS*_rv	AGCTACTCGAGTTACTTTTCGAACTGCGGGTGGCTCCATGCGCTATCGTTCTTTT CGGAAACCAATTC
C13S_fw	GTTTATGGTCCGGCAGTTGCAGTTAGCCCGCAGCGTGTTATGG
C13X_fw	GTTTATGGTCCGGCAGTTGCAGTTNNKCCACAGCGTGTTATGGCATGTC
*CrCPR*_OL_fw	GGAACAGGTGATCATGTAGG
*CrCPR*_OL_rv	CCTACATGATCACCTGTTCC
F112_rv	CACCAGGTCATTGAAATTATGTG
F112X_fw	CACATAATTTCAATGACCTGGTGNNKAATATTGTGTTTCAGGTTGTTATTCTG
MET15 outer fw	GGGTTCGAATCCCTTAGCTCTC
MET15 outer rv	ACCAACTGGGCCAAGAGACC
*MET15*_fw	AGGTCACATGATCGCAAAATGGC
*MET15*_rv	GGACATATTAAACTATGACGACATTGTTGC
N108_rv	GAAATTATGTGCTTCAATTTCCAGC
N108X_fw	GCTGGAAATTGAAGCACATAATTTCNNKGATCTGGTGTTCAACATTGTGTTTC
NdeI_*AtANS*_fw	AGCTACATATGGTTGCCGTTGAAAGAG
*PhAN9*_A12M_fw	GTTCATGGTTCTGCTATGGCTATGTGTCCACAAAGAGTTATGGTTTG
*PhAN9*_A12M_rv	AGCCATAGCAGAACCATGAAC
*PhAN9*_C13S_fw	GGTTCTGCTATGGCTGCTAGCCCACAAAGAGTTATGGTTTGC
*PhAN9*_C13S_rv	AGCAGCCATAGCAGAACC
*PhAN9*_N108H_fw	GTTGGAAGTCGAATCTAACAACTACCATGATCTGGTTTACAACATGGTGTTG
*PhAN9*_N108H_rv	GTAGTTGTTAGATTCGACTTCCAAC
*PtGSTF8*_rv	AGCTACCGCGGTTAAAAACCTGCCAGGTTCATC
*ScAtANS*_fw	AGCTAAAGCTTAAAATGGTTGCCGTTGAAAGAG
*ScAtANS*_rv	AGCTACCGCGGTTAATCGTTCTTTTCGGAAACC
*ScAtTT19*_fw	AGCTAAAGCTTAAAATGGTCGTTAAGTTGTACGG
*ScAtTT19*_rv	AGCTACCGCGGTCAGTGACCAGCCAAAAC
*ScPhAN9*_fw	AGCTAAAGCTTAAAATGGTTGTTAAGGTTCATGGTTC
*ScPhAN9*_rv	AGCTACCGCGGTCAGACCTTAGCTTCTTCTTTAG
*ScPhANS*_fw	AGCTAAAGCTTAAAATGGTTAACGCTGTTGTTAC
*ScPhANS*_rv	AGCTACCGCGGTTACTTAGATTCTTCAGCGGC
*ScPtGSTF8*_fw	AGCTAAAGCTTAAAATGGTGGTGAAAGTTTATGGTC
*ScVvGST1*_fw	AGCTAAAGCTTAAAATGGCCAACTCTGATCATATC
*ScVvGST1*_rv	AGCTACCGCGGTCAGATGCCCATCTTCTTTC
*ScVvGST2*_fw	AGCTAAAGCTTAAAATGGCCGTTTTGAAAGTTC

*ScVvGST2*_rv	AGCTACCGCGGTTAGGACTTTTGCATAGCAATAAC
*ScVvGST3*_fw	AGCTAAAGCTTAAAATGGTTGTTAAGGTTTACGGTC
*ScVvGST3*_rv	AGCTACCGCGGTCACTCCAATAATGGCCATC
*ScVvGST4*_fw	AGCTAAAGCTTAAAATGGTCATGAAGGTTTATGGTC
*ScVvGST4*_rv	AGCTACCGCGGTTAAGCAGCCAATTCCATGAC
*ScVvGST5*_fw	AGCTAAAGCTTAAAATGGCCGACGAAATTATCTTG
*ScVvGST5*_rv	AGCTACCGCGGCTACTCGATACCGAATCTCTTTC
*ScZmBZ2*_fw	AGCTAAAGCTTAAAATGACTGCTGGTACTATGAGAG
*ScZmBZ2*_rv	AGCTACCGCGGTCAAGAAACGTGGACTCTG
T7 fw	TAATACGACTCACTATAGGG
T7 term	GCTAGTTATTGCTCAGCGG
tADH_fw	AGCTACCGCGGGCTTTGGACTTCTTCGCC
tADH_rv	AGCTAGCATGCGGTAGAGGTGTGGTCAATAAGAG
V12C13_rv	AACTGCCGGACCATAAAC
V12X_fw	GTTTATGGTCCGGCAGTTGCANNKTGCCCGCAGCGTGTTATGG
*VvGST4*_A12M_fw	GAAGGTTTATGGTCCAGTTAGAGCTATGTGTCCACAAAGAGTTTTGGC
*VvGST4*_A12M_rv	AGCTCTAACTGGACCATAAACCTTC
*VvGST4*_C13S_fw	GTTTATGGTCCAGTTAGAGCTGCTAGCCCACAAAGAGTTTTGGCTTG
*VvGST4*_C13S_rv	AGCAGCTCTAACTGGACCATAAAC
*VvGST4*_N108H_fw	GTTGGAAGTTGAAGCTCACAACTTTCATGAATTGGTTTACACCTTGGTCATG
*VvGST4*_N108H_rv	AAAGTTGTGAGCTTCAACTTCCAAC

Supplementary Table 5. List of yeast strains used in this study.

Name	Genotype	Construction
S288C	*MATα SUC2 gal2 mal2 mel flo1 flo8-1 hap1 ho bio1 bio6*	Obtained from NCYC
BY4741	*MATa his3Δ1 leu2Δ0 met15Δ0 ura3Δ0*	Obtained from Euroscarf
BY4741_ULH	*MATa his3Δ1 leu2Δ0 ura3Δ0*	Integration of PCR product with primers MET15_fw and MET15_rv from genomic DNA of *S. cerevisiae* S288C
NAR	BY_4741_ULH [ARS/CEN/*URA3*/P_{GPD1}-*MdCHS2*-T_{TPS1}/P_{PGK1}-*MsCHI*-T_{ADH2}/P_{TEF1}-*At4CL2*-T_{ENO2}/P_{PDC1}-*AtPAL2*-T_{FBA1}/P_{TEF2}-*AmC4H*-T_{PGI1}/P_{PYK1}-*AtCPR1*_co2-T_{ADH1}]	Assembly of multi-expression plasmid from *AscI* digested pANT22, pANT25, pANT27, pNAT29, pANT31, pANT81, pHR14, pHR16
P3G	NAR [ARS/CEN/*HIS3*/P_{GPD1}-*AtLDOX*-T_{TPS1}/P_{PGK1}-*FaA3GT2*-T_{ADH2}/P_{TEF1}-*MdF3H*-T_{ENO2}/P_{PDC1}-*PtDFR*-T_{ADH1}]	Assembly of multi-expression plasmid from *AscI* digested pANT23, pANT26, pANT28, pANT55, pHR14, pHR15
C3G1	P3G [ARS/CEN/*LEU2*/P_{GPD1}-*ZmBZ2*-T_{TPS1}/P_{PGK1}-*OsF3'H*-T_{ADH2}/P_{TEF1}-*CrCPR*-T_{ADH1}]	Assembly of multi-expression plasmid from *AscI* digested pANT57, pANT78, pANT80, pHR14, pHR17
C3G2	P3G [ARS/CEN/*LEU2*/P_{GPD1}-*PhAN9*-T_{TPS1}/P_{PGK1}-*OsF3'H*-T_{ADH2}/P_{TEF1}-*CrCPR*-T_{ADH1}]	Assembly of multi-expression plasmid from *AscI* digested pANT58, pANT78, pANT80, pHR14, pHR17
C3G3	P3G [ARS/CEN/*LEU2*/P_{GPD1}-*AtTT19*-T_{TPS1}/P_{PGK1}-*OsF3'H*-T_{ADH2}/P_{TEF1}-*CrCPR*-T_{ADH1}]	Assembly of multi-expression plasmid from *AscI* digested pANT59, pANT78, pANT80, pHR14, pHR17
C3G4	P3G [ARS/CEN/*LEU2*/P_{GPD1}-*VvGST4*-T_{TPS1}/P_{PGK1}-*OsF3'H*-T_{ADH2}/P_{TEF1}-*CrCPR*-T_{ADH1}]	Assembly of multi-expression plasmid from *AscI* digested pANT73, pANT78, pANT80, pHR14, pHR17
C3G5	P3G [ARS/CEN/*LEU2*/P_{GPD1}-*PtGSTF8*-T_{TPS1}/P_{PGK1}-*OsF3'H*-T_{ADH2}/P_{TEF1}-*CrCPR*-T_{ADH1}]	Assembly of multi-expression plasmid from *AscI* digested pANT75, pANT78, pANT80, pHR14, pHR17
C3G6	P3G [ARS/CEN/*LEU2*/P_{GPD1}-*VvGST1*-T_{TPS1}/P_{PGK1}-*OsF3'H*-T_{ADH2}/P_{TEF1}-*Cr*CPR-T_{ADH1}]	Assembly of multi-expression plasmid from *AscI* digested pANT70, pANT78, pANT80, pHR14, pHR17
C3G7	P3G [ARS/CEN/LEU2/P_{GPD1}-VvGST2-T_{TPS1}/P_{PGK1}-*OsF3'H*-T_{ADH2}/P_{TEF1}-*Cr*CPR-T_{ADH1}]	Assembly of multi-expression plasmid from *AscI* digested pANT71, pANT78, pANT80, pHR14, pHR17
C3G8	P3G [ARS/CEN/LEU2/P_{GPD1}-VvGST3-T_{TPS1}/P_{PGK1}-*OsF3'H*-T_{ADH2}/P_{TEF1}-*CrCPR*-T_{ADH1}]	Assembly of multi-expression plasmid from *AscI* digested pANT72, pANT78, pANT80, pHR14, pHR17
C3G9	P3G [ARS/CEN/*LEU2*/P_{GPD1}-*VvGST5*-T_{TPS1}/P_{PGK1}-*OsF3'H*-T_{ADH2}/P_{TEF1}-*CrCPR*-T_{ADH1}]	Assembly of multi-expression plasmid from *AscI* digested pANT74, pANT78, pANT80, pHR14, pHR17
C3G10	P3G [ARS/CEN/*LEU2*/P_{GPD1}-T_{TPS1}/P_{PGK1}-*OsF3'H*-T_{ADH2}/P_{TEF1}-*CrCPR*-T_{ADH1}]	Assembly of multi-expression plasmid from *AscI* digested pHR1, pANT78, pANT80, pHR14, pHR17
DHQC3G1	BY_4741_ULH [ARS/CEN/*URA3*/P_{GPD1}-*ZmBZ2*-T_{TPS1}/P_{PGK1}-*FaA3GT2*-T_{ADH2}/P_{TEF1}-*PhLDOX*-T_{ENO2}/ P_{PDC1}-*PtDFR*-T_{ADH1}]	Assembly of multi-expression plasmid from *AscI* digested pANT57, pANT26, pANT86, pANT55, pHR14, pHR16
DHQC3G2	BY_4741_ULH [ARS/CEN/*URA3*/P_{GPD1}-*PhAN9*-T_{TPS1}/P_{PGK1}-*FaA3GT2*-T_{ADH2}/P_{TEF1}-*PhLDOX*-T_{ENO2}/ P_{PDC1}-*PtDFR*-T_{ADH1}]	Assembly of multi-expression plasmid from *AscI* digested pANT58, pANT26, pANT86, pANT55, pHR14, pHR16
DHQC3G3	BY_4741_ULH [ARS/CEN/*URA3*/P_{GPD1}-*AtTT19*-T_{TPS1}/P_{PGK1}-*FaA3GT2*-T_{ADH2}/P_{TEF1}-*PhLDOX*-T_{ENO2}/ P_{PDC1}-*PtDFR*-T_{ADH1}]	Assembly of multi-expression plasmid from *AscI* digested pANT59, pANT26, pANT86, pANT55, pHR14, pHR16

DHQC3G4	BY_4741_ULH [ARS/CEN/*URA3*/P_{GPD1}-*VvGST4*-T_{TPS1}/P_{PGK1}-*FaA3GT2*-T_{ADH2}/P_{TEF1}-*PhLDOX*-T_{ENO2}/ P_{PDC1}-*PtDFR*-T_{ADH1}]	Assembly of multi-expression plasmid from *AscI* digested pANT73, pANT26, pANT86, pANT55, pHR14, pHR16
DHQC3G5	BY_4741_ULH [ARS/CEN/*URA3*/P_{GPD1}-*PtGSTF8*-T_{TPS1}/P_{PGK1}-*FaA3GT2*-T_{ADH2}/P_{TEF1}-*PhLDOX*-T_{ENO2}/ P_{PDC1}-*PtDFR*-T_{ADH1}]	Assembly of multi-expression plasmid from *AscI* digested pANT75, pANT26, pANT86, pANT55, pHR14, pHR16
DHQC3G6	BY_4741_ULH [ARS/CEN/*URA3*/P_{GPD1}-*VvGST1*-T_{TPS1}/P_{PGK1}-*FaA3GT2*-T_{ADH2}/P_{TEF1}-*PhLDOX*-T_{ENO2}/ P_{PDC1}-*PtDFR*-T_{ADH1}]	Assembly of multi-expression plasmid from *AscI* digested pANT70, pANT26, pANT86, pANT55, pHR14, pHR16
DHQC3G7	BY_4741_ULH [ARS/CEN/*URA3*/P_{GPD1}-*VvGST2*-T_{TPS1}/P_{PGK1}-*FaA3GT2*-T_{ADH2}/P_{TEF1}-*PhLDOX*-T_{ENO2}/ P_{PDC1}-*PtDFR*-T_{ADH1}]	Assembly of multi-expression plasmid from *AscI* digested pANT71, pANT26, pANT86, pANT55, pHR14, pHR16
DHQC3G8	BY_4741_ULH [ARS/CEN/*URA3*/P_{GPD1}-*VvGST3*-T_{TPS1}/P_{PGK1}-*FaA3GT2*-T_{ADH2}/P_{TEF1}-*PhLDOX* -T_{ENO2}/ P_{PDC1}-*PtDFR*-T_{ADH1}]	Assembly of multi-expression plasmid from *AscI* digested pANT72, pANT26, pANT86, pANT55, pHR14, pHR16
DHQC3G9	BY_4741_ULH [ARS/CEN/*URA3*/P_{GPD1}-*VvGST5*-T_{TPS1}/P_{PGK1}-*FaA3GT2*-T_{ADH2}/P_{TEF1}-*PhLDOX*-T_{ENO2}/ P_{PDC1}-*PtDFR*-T_{ADH1}]	Assembly of multi-expression plasmid from *AscI* digested pANT74, pANT26, pANT86, pANT55, pHR14, pHR16
DHQC3G10	BY_4741_ULH [ARS/CEN/URA3/P_{GPD1}-T_{TPS1}/P_{PGK1}-*FaA3GT2*-T_{ADH2}/P_{TEF1}-*PhLDOX*-T_{ENO2}/ P_{PDC1}-*PtDFR*-T_{ADH1}]	Assembly of multi-expression plasmid from *AscI* digested pHR1, pANT26, pANT86, pANT55, pHR14, pHR16

Supplementary Table 6. Coding sequences of enzymes in this study

Gene	Coding sequence
AmC4H	ATGATGGACTTCGTCTTGTTGGAAAAGGCTTTGTTGGGTTTGTTCATTGCTACCATCGTTGCC<br>ATTACCATCTCTAAATTGAGAGGCAAGAAACTGAAATTGCCACCAGGTCCAATTCCAGTTCCA<br>GTTTTTGGTAATTGGTTGCAAGTTGGTGATGACTTGAATCAGAGAAACTTGGTTGAATACGCC<br>AAAAAGTTCGGTGACTTGTTCTTGTTGAGAATGGGTCAAAGAAATTTGGTCGTTGTTTCCTCA<br>CCAGATTTGGCTAAAGATGTCTTGCATACTCAAGGTGTTGAATTCGGTTCCAGAACTAGAAAT<br>GTTGTGTTCGATATTTTCACCGGTAAGGGTCAAGATATGGTTTTCACTGTTTACTCCGAACAT<br>TGGAGAAAGATGAGAAGAATTATGACGGTTCCATTCTTCACCAACAAGGTTGTTCAACAGTAT<br>AGATTCGGTTGGGAAGATGAAGCTGCTAGAGTTGTTGAAGATGTTAAGGCTAATCCTGAAGCT<br>GCAACTAATGGTATCGTTTTGAGAAACAGACTGCAGCTGCTGATGTACAACAACATGTACAGA<br>ATCATGTTCGACAGAAGGTTCGAATCTGTTGATGATCCTTTGTTCTTGAAGTTGAAGGCTTTG<br>AACGGTGAAAGATCAAGATTGGCTCAATCCTTTGAGTACAACTTCGGTGATTTCATCCCAATT<br>TTGAGGCCATTTTTGAGGGGCTACTTGAAATTGTGTCAAGAGATCAAGGACAAGAGGCTGAAG<br>TTGTTTAAGGATTACTTTGTCGACGAGAGGAAGAAGTTGGAATCCATTAAGTCTGTTGGCAAC<br>AACTCCTTGAAGTGCGCTATTGATCATATTATCGAAGCCCAAGAAAAGGGTGAAATCAATGAA<br>GATAACGTCTTGTACATCGTCGAGAACATTAACGTTGCTGCTATTGAAACTACCTTGTGGTCT<br>ATTGAATGGGGTATTGCTGAATTGGTTAACAACCCAGAAATCCAGAAAAAGTTGAGACACGAA<br>TTGGATACCGTTTTAGGTGCTGGTGTTCAAATTTGTGAACCAGATGTTCAAAAGCTGCCATAC<br>TTGCAAGCTGTTATCAAAGAAACCTTGAGATACAGAATGGCTATCCCTTTGTTGGTTCCACAC<br>ATGAACTTGCATGAAGCTAAATTGGCTGGTTACGATATTCCAGCCGAATCCAAGATTTTGGTT<br>AATGCTTGGTGGTTGGCTAACAATCCAGCTCATTGGAACAAACCAGATGAATTCAGACCAGAA<br>AGGTTCTTGGAAGAAGAATCTAAGGTTGAAGCTAACGGTAACGACTTCAAGTACATTCCATTT<br>GGTGTTGGTAGAAGATCATGCCCAGGTATTATCTTGGCTTTGCCAATTTTGGGTATCGTCATA<br>GGTAGATTGGTTCAGAATTTCGAGTTGTTACCACCACCTGGTCAATCTAAAATTGATACCGCT<br>GAAAAAGGTGGCCAATTCTCCTTGCAAATCTTGAAACATTCTACGATTGTCTGCAAGCCAAGG<br>TCCTCTTAA
At4CL2	ATGACTACCCAAGATGTTATCGTCAACGATCAAAACGACCAAAAGCAATGTTCCAACGATGTC<br>ATCTTCAGATCAAGATTGCCAGATATCTACATCCCAAACCATTTGCCATTGCACGATTACATC<br>TTCGAAAACATTTCTGAATTCGCTGCTAAGCCATGCTTGATTAACGGTCCAACTGGTGAAGTT<br>TACACTTACGCTGATGTTCATGTTACCTCCAGAAAATTGGCTGCTGGTTTACACAATTTGGGT<br>GTTAAGCAACACGATGTCGTTATGATTTTGTTGCCAAACTCTCCAGAAGTTGTCTTGACTTTT<br>TTGGCTGCTTCTTTCATTGGTGCTATTACTACTTCTGCTAACCCATTTTTTACCCCAGCCGAA<br>ATTTCTAAACAAGCTAAGGCTTCTGCTGCCAAGTTGATAGTTACTCAATCAAGATACGTCGAC<br>AAGATCAAGAACTTGCAAAACGATGGTGTTTTGATTGTCACCACTGATTCTGATGCTATTCCA<br>GAAAACTGCTTGAGGTTCTCTGAATTGACTCAATCTGAAGAACCTAGGGTTGATTCCATTCCA<br>GAGAAAATTTCACCAGAAGATGTTGTTGCTTTGCCCTTTTCTTCTGGTACTACTGGTTTGCCA<br>AAAGGTGTTATGTTGACTCACAAAGGTTTGGTTACATCCGTTGCTCAACAAGTTGATGGTGAA<br>AATCCAAACTTGTACTTCAACAGGGATGACGTTATTTTGTGCGTTTTGCCAATGTTTCATATC<br>TACGCCTTGAACTCCATCATGTTGTGTTCTTTGAGAGTTGGTGCCACCATTTTGATTATGCCA<br>AAGTTCGAAATCACCCTGTTGTTGGAACAAATCCAAAGATGTAAGGTTACCGTTGCTATGGTT<br>GTTCCACCAATAGTTTTGGCTATTGCTAAGTCTCCAGAAACCGAAAAGTACGATTTGTCCTCT<br>GTTAGAATGGTTAAGTCTGGTGCTGCTCCATTGGGTAAAGAATTGGAAGATGCTATTTCTGCT<br>AAGTTCCCAAACGCTAAATTAGGTCAAGGTTACGGTATGACTGAAGCTGGTCCAGTTTTAGCT<br>ATGTCTTTGGGTTTTGCTAAAGAGCCATTTCCAGTAAAATCTGGTGCTTGTGGTACTGTTGTT<br>AGAAACGCTGAGATGAAGATTTTGGACCCAGATACTGGTGATTCTTTGCCAAGAAACAAACCA<br>GGTGAAATCTGCATCAGAGGTAACCAGATTATGAAGGGTTACTTGAATGATCCATTGGCTACT<br>GCTTCTACCATTGATAAGGATGGTTGGTTGCATACAGGTGATGTTGGTTTCATAGATGATGAT<br>GACGAGTTGTTCATCGTCGACAGATTGAAAGAACTGATCAAGTACAAGGGTTTCCAAGTTGCT<br>CCAGCTGAATTGGAATCTTTGTTGATTGGTCATCCAGAAATCAACGATGTTGCTGTTGTTGCA<br>ATGAAGGAAGAGGACGCTGGCGAAGTTCCAGTTGCTTTCGTTGTTAGATCAAAGGACTCTAAT<br>ATCTCCGAGGACGAAATCAAGCAATTCGTTTCAAAGCAAGTCGTGTTCTACAAGAGAATCAAC<br>AAGGTTTTCTTCACCGACTCTATTCCAAAAGCTCCATCTGGTAAGATTTTGAGAAAGGATTTG<br>AGAGCTAGATTGGCCAACGGTTTGATGAACTAA
*AtCPR1*_co1	ATGACTTCTGCCTTGTATGCCTCTGATTTGTTCAAGCAATTGAAGTCCATTATGGGCACCGAT<br>TCTTTGTCTGATGATGTTGTTTTGGTTATCGCTACTACCTCTTTGGCTTTGGTTGCTGGTTTT<br>GTTGTTCTGTTGTGGAAAAAGACTACCGCTGATAGATCAGGTGAATTGAAACCATTGATGATC<br>CCCAAATCTTTGATGGCCAAAGATGAAGATGATGACTTGGACTTAGGTTCTGGTAAGACTAGA<br>GTTTCCATTTTCTTCGGTACTCAAACTGGTACTGCTGAAGGTTTTGCTAAGGCTTTATCTGAA<br>GAAATCAAGGCCAGATACGAAAAGGCTGCTGTTAAGGTTATTGATTTGGATGATTACGCTGCC<br>GATGATGACCAATACGAAGAAAAGTTGAAGAAAGAAACCTTGGCCTTCTTCTGTGTTGCTACT

TATGGTGATGGTGAACCTACTGATAATGCTGCTAGATTTTACAAGTGGTTCACCGAAGAGAAC
GAAAGAGATATCAAGTTGCAACAATTGGCCTACGGTGTTTTTGCTTTGGGTAATAGACAATAC
GAGCACTTCAACAAGATCGGTATCGTTTTGGATGAAGAGTTGTGTAAAAAGGGTGCCAAGAGA
TTGATTGAAGTTGGTTTGGGTGATGACGACCAATCTATCGAAGATGATTTTAACGCCTGGAAA
GAATCCTTGTGGTCTGAATTGGATAAGTTGTTGAAGGACGAAGATGACAAATCTGTTGCTACA
CCATACACTGCTGTTATTCCAGAGTATAGAGTTGTTACTCACGATCCAAGATTCACGACTCAA
AAGTCTATGGAATCTAACGTTGCTAACGGTAACACCACCATCGATATTCATCATCCATGTAGA
GTTGATGTCGCCGTCCAAAAAGAATTGCATACTCATGAATCCGATAGGTCCTGCATTCATTTG
GAATTCGATATTTCCAGAACCGGTATTACTTACGAAACCGGTGATCATGTTGGTGTTTACGCT
GAAAATCACGTTGAAATCGTTGAAGAAGCCGGTAAGTTGTTAGGTCATTCATTGGATTTGGTG
TTCTCCATTCATGCCGACAAAGAAGATGGTTCTCCTTTGGAATCTGCTGTTCCACCACCATTT
CCAGGTCCATGTACTTTAGGTACTGGTTTGGCTAGATATGCTGACTTGTTGAATCCACCAAGA
AAGTCTGCTTTAGTTGCTTTGGCTGCTTATGCTACTGAACCATCTGAAGCCGAAAAATTGAAA
CATTTGACTTCCCCAGATGGTAAGGACGAATATTCTCAATGGATAGTTGCTTCCCAAAGGTCC
TTGTTGGAAGTTATGGCTGCTTTTCCATCTGCTAAACCACCATTGGGTGTTTTTTTTGCTGCT
ATTGCTCCAAGATTGCAACCTAGGTATTACTCCATTTCTTCATCACCAAGATTGGCCCCATCA
AGAGTTCATGTTACATCTGCTTTGGTTTATGGTCCAACTCCAACTGGTAGAATTCATAAGGGT
GTTTGTTCTACCTGGATGAAGAACGCTGTTCCAGCTGAAAAATCTCATGAATGTTCTGGTGCC
CCAATTTTCATTAGAGCTTCTAATTTCAAGCTGCCAAGCAATCCATCTACTCCAATAGTTATG
GTTGGTCCAGGTACAGGTTTAGCTCCTTTTAGAGGTTTCCTACAAGAAAGGATGGCCTTGAAA
GAGGATGGCGAAGAATTGGGTTCTTCCTTGTTGTTTTTTGGTTGCAGAAACAGACAGATGGAT
TTCATCTATGAGGACGAGTTGAACAACTTCGTTGATCAAGGTGTTATCTCCGAATTGATTATG
GCCTTTTCAAGAGAAGGTGCCCAGAAAGAATATGTCCAACATAAGATGATGGAAAAAGCTGCC
CAAGTTTGGGACCTAATCAAAGAAGAAGGATACTTGTACGTTTGCGGTGATGCTAAAGGTATG
GCTAGAGATGTTCATAGAACATTGCATACCATCGTCCAAGAACAAGAAGGTGTTTCATCTTCT
GAAGCTGAAGCTATCGTTAAGAAGTTGCAAACTGAAGGTAGATACTTGAGAGATGTCTGGTGA

*AtCPR1*_co2

ATGACTTCTGCACTTTATGCCTCCGATCTTTTCAAACAATTGAAAAGTATCATGGGAACGGAT
TCTTTGTCCGATGATGTTGTATTAGTTATTGCTACAACTTCTCTGGCACTGGTTGCTGGTTTC
GTTGTCTTATTGTGGAAAAAGACCACGGCAGATCGTTCCGGCGAGCTAAAGCCACTAATGATC
CCTAAGTCTCTGATGGCGAAAGATGAGGATGATGACTTAGATCTAGGTTCTGGAAAAACGAGA
GTCTCTATCTTCTTCGGCACACAAACCGGAACAGCCGAAGGATTCGCTAAAGCACTTTCAGAA
GAGATCAAAGCAAGATACGAAAAGGCGGCTGTAAAAGTAATCGATTTGGATGATTACGCTGCC
GATGATGACCAATATGAGGAAAAGTTGAAAAAGGAAACATTGGCTTTCTTTTGTGTAGCCACG
TATGGTGATGGTGAACCAACCGATAACGCCGCAAGATTCTACAAGTGGTTTACTGAAGAGAAC
GAAAGAGATATCAAGTTGCAGCAACTTGCTTACGGCGTTTTTGCCTTAGGTAACAGACAATAC
GAGCACTTTAACAAGATAGGTATTGTCTTAGATGAAGAGTTATGCAAAAAGGGTGCGAAGAGA
TTGATTGAAGTCGGTTTAGGAGATGATGATCAATCTATCGAGGATGACTTTAATGCATGGAAG
GAATCTTTGTGGTCTGAATTAGATAAGTTACTTAAGGACGAAGATGATAAATCCGTTGCCACT
CCATACACAGCCGTCATTCCAGAATATAGAGTAGTTACTCATGATCCAAGATTCACAACACAG
AAATCAATGGAAAGTAATGTGGCTAATGGTAATACTACCATCGATATTCATCATCCATGTAGA
GTAGACGTTGCAGTTCAAAAGGAATTGCACACTCATGAATCAGACAGATCTTGCATACATCTT
GAATTTGATATATCACGTACTGGTATCACTTACGAAACAGGTGATCACGTGGGTGTCTACGCT
GAAAACCATGTTGAAATTGTAGAGGAAGCTGGAAAGTTGTTGGGCCATAGTTTAGATCTTGTT
TTCTCAATTCATGCCGATAAAGAGGATGGCTCACCACTAGAAAGTGCAGTGCCTCCACCATTT
CCAGGACCATGCACCCTAGGTACCGGTTTAGCTCGTTACGCGGATCTGTTAAATCCTCCACGT
AAATCAGCTCTAGTGGCCTTGGCTGCGTACGCCACAGAACCTTCTGAGGCAGAAAAACTGAAA
CATCTAACTTCACCAGATGGTAAGGATGAATACTCACAATGGATAGTAGCTAGTCAACGTTCT
TTACTAGAAGTTATGGCTGCTTTCCCATCCGCTAAACCTCCTTTGGGTGTTTTCTTCGCCGCA
ATAGCGCCTAGACTGCAACCAAGATACTATTCAATTTCATCCTCACCTAGACTGGCACCATCA
AGAGTTCATGTCACATCCGCTTTAGTGTACGGTCCAACTCCTACTGGTAGAATCCATAAGGGC
GTTTGTTCAACATGGATGAAAAACGCGGTTCCAGCAGAGAAGTCTCACGAATGTTCTGGTGCT
CCAATCTTTATCAGAGCCTCCAACTTCAAACTGCCTTCCAATCCTTCTACTCCTATTGTCATG
GTCGGTCCTGGTACAGGTCTTGCTCCATTCAGAGGTTTCTTACAAGAGAGAATGGCCTTAAAG
GAGGATGGTGAAGAGTTGGGATCTTCTTTGTTGTTTTTCGGCTGTAGAAACAGACAAATGGAT
TTCATCTACGAAGATGAACTGAATAACTTTGTAGATCAAGGAGTTATTTCAGAGTTGATAATG
GCTTTTTCTAGAGAAGGTGCTCAGAAGGAGTACGTCCAACACAAAATGATGGAAAAGGCCGCA
CAAGTTTGGGACTTAATCAAAGAGGAAGGCTATCTATATGTCTGTGGTGATGCAAAGGGTATG
GCAAGAGATGTTCACAGAACACTTCATACTATAGTCCAGGAACAGGAAGGCGTTAGTTCTTCT
GAAGCGGAAGCAATTGTGAAAAAGTTACAAACAGAGGGAAGATACTTGAGAGATGTGTGGTAA

AtGSTF2

ATGGCAGGCATTAAAGTTTTTGGTCATCCGGCAAGCATTGCAACCCGTCGTGTTCTGATTGCA
CTGCATGAAAAAAACCTGGATTTTGAACTGGTTCACGTGGAACTGAAAGATGGCGAACATAAA
AAAGAACCGTTTCTGAGCCGTAATCCGTTTGGTCAGGTTCCGGCATTTGAAGATGGTGATCTG
AAACTGTTTGAAAGCCGTGCAATTACCCAGTATATTGCCCATCGTTATGAAAATCAGGGCACC
AATCTGCTGCAGACCGATAGCAAAAACATTAGCCAGTATGCAATTATGGCCATCGGTATGCAG
GTTGAAGATCATCAGTTTGATCCGGTTGCAAGCAAACTGGCCTTTGAGCAGATTTTCAAAAGC
ATTTATGGTCTGACCACCGATGAAGCAGTTGTTGCAGAAGAAGAAGCAAAACTGGCAAAAGTT
CTGGATGTTTATGAAGCCCGTCTGAAAGAGTTTAAATACCTGGCAGGCGAAACCTTTACACTG
ACCGATCTGCATCATATTCCGGCAATTCAGTATCTGCTGGGCACCCCGACCAAAAAACTGTTT
ACCGAACGTCCGCGTGTTAATGAATGGGTTGCAGAAATTACAAAACGTCCTGCAAGCGAAAAA
GTGCAGTAA

AtLDOX

ATGGTTGCCGTTGAAAGAGTTGAATCTTTGGCTAAGTCCGGTATTATCTCCATTCCAAAAGAG
TACATCAGGCCCAAAGAAGAATTGGAATCCATCAACGATGTGTTCTTGGAAGAAAAGAAAGAA
GATGGTCCACAAGTTCCAACCATCGATTTGAAGAACATTGAATCCGATGACGAGAAGATCAGA
GAAAACTGCATTGAGGAATTGAAGAAGGCTTCTTTGGATTGGGGTGTTATGCATTTGATTAAC
CACGGTATTCCAGCCGATTTGATGGAAAGGGTTAAGAAAGCTGGTGAAGAGTTCTTCTCATTG
TCCGTCGAAGAAAAAGAAAAGTACGCTAACGATCAAGCCACCGGTAAAATTCAAGGTTACGGT
TCTAAATTGGCTAACAACGCTTCTGGTCAATTGGAATGGGAAGATTACTTTTTCCATTTGGCT
TACCCAGAAGAGAAGAGAGATTTGTCTATTTGGCCTAAGACTCCATCCGATTACATTGAAGCT
ACATCTGAATACGCTAAGTGCTTGAGATTATTGGCTACCAAGGTTTTCAAGGCTTTGTCTGTT
GGTTTGGGTTTAGAACCAGACAGGTTGGAAAAAGAAGTTGGAGGTTTGGAAGAGTTGTTGTTG
CAAATGAAGATCAACTACTACCCAAAGTGTCCACAACCAGAATTGGCTTTGGGTGTTGAAGCT
CATACTGATGTTTCTGCTTTGACCTTCATCTTGCATAATATGGTTCCAGGCTTGCAGTTGTTC
TATGAAGGTAAATGGGTTACCGCTAAGTGTGTTCCAGATTCTATCGTTATGCATATCGGTGAT
ACCCTGGAAATTTTGTCTAATGGCAAGTACAAGTCCATCTTGCACAGAGGTTTGGTCAACAAA
GAAAAGGTTAGAATTTCCTGGGCTGTTTTCTGTGAACCACCAAAAGATAAGATCGTCTTGAAA
CCATTGCCAGAGATGGTTTCTGTTGAATCTCCAGCTAAATTTCCACCAAGAACTTTCGCCCAA
CATATCGAACATAAGTTGTTCGGTAAAGAGCAAGAGGAATTGGTTTCCGAAAAGAACGATTAA

Strep-tag-*AtLDOX*

ATGGCAAGCTGGAGCCACCCGCAGTTCGAAAAGGGTGCAATGGTTGCCGTTGAAAGAGTTGAA
TCTTTGGCTAAGTCCGGTATTATCTCCATTCCAAAAGAGTACATCAGGCCCAAAGAAGAATTG
GAATCCATCAACGATGTGTTCTTGGAAGAAAAGAAAGAAGATGGTCCACAAGTTCCAACCATC
GATTTGAAGAACATTGAATCCGATGACGAGAAGATCAGAGAAAACTGCATTGAGGAATTGAAG
AAGGCTTCTTTGGATTGGGGTGTTATGCATTTGATTAACCACGGTATTCCAGCCGATTTGATG
GAAAGGGTTAAGAAAGCTGGTGAAGAGTTCTTCTCATTGTCCGTCGAAGAAAAAGAAAAGTAC
GCTAACGATCAAGCCACCGGTAAAATTCAAGGTTACGGTTCTAAATTGGCTAACAACGCTTCT
GGTCAATTGGAATGGGAAGATTACTTTTTCCATTTGGCTTACCCAGAAGAGAAGAGAGATTTG
TCTATTTGGCCTAAGACTCCATCCGATTACATTGAAGCTACATCTGAATACGCTAAGTGCTTG
AGATTATTGGCTACCAAGGTTTTCAAGGCTTTGTCTGTTGGTTTGGGTTTAGAACCAGACAGG
TTGGAAAAAGAAGTTGGAGGTTTGGAAGAGTTGTTGTTGCAAATGAAGATCAACTACTACCCA
AAGTGTCCACAACCAGAATTGGCTTTGGGTGTTGAAGCTCATACTGATGTTTCTGCTTTGACC
TTCATCTTGCATAATATGGTTCCAGGCTTGCAGTTGTTCTATGAAGGTAAATGGGTTACCGCT
AAGTGTGTTCCAGATTCTATCGTTATGCATATCGGTGATACCCTGGAAATTTTGTCTAATGGC
AAGTACAAGTCCATCTTGCACAGAGGTTTGGTCAACAAAGAAAAGGTTAGAATTTCCTGGGCT
GTTTTCTGTGAACCACCAAAAGATAAGATCGTCTTGAAACCATTGCCAGAGATGGTTTCTGTT
GAATCTCCAGCTAAATTTCCACCAAGAACTTTCGCCCAACATATCGAACATAAGTTGTTCGGT
AAAGAGCAAGAGGAATTGGTTTCCGAAAAGAACGATTAA

AtLDOX-Strep-tag

ATGGTTGCCGTTGAAAGAGTTGAATCTTTGGCTAAGTCCGGTATTATCTCCATTCCAAAAGAG
TACATCAGGCCCAAAGAAGAATTGGAATCCATCAACGATGTGTTCTTGGAAGAAAAGAAAGAA
GATGGTCCACAAGTTCCAACCATCGATTTGAAGAACATTGAATCCGATGACGAGAAGATCAGA
GAAAACTGCATTGAGGAATTGAAGAAGGCTTCTTTGGATTGGGGTGTTATGCATTTGATTAAC
CACGGTATTCCAGCCGATTTGATGGAAAGGGTTAAGAAAGCTGGTGAAGAGTTCTTCTCATTG
TCCGTCGAAGAAAAAGAAAAGTACGCTAACGATCAAGCCACCGGTAAAATTCAAGGTTACGGT
TCTAAATTGGCTAACAACGCTTCTGGTCAATTGGAATGGGAAGATTACTTTTTCCATTTGGCT
TACCCAGAAGAGAAGAGAGATTTGTCTATTTGGCCTAAGACTCCATCCGATTACATTGAAGCT
ACATCTGAATACGCTAAGTGCTTGAGATTATTGGCTACCAAGGTTTTCAAGGCTTTGTCTGTT
GGTTTGGGTTTAGAACCAGACAGGTTGGAAAAAGAAGTTGGAGGTTTGGAAGAGTTGTTGTTG
CAAATGAAGATCAACTACTACCCAAAGTGTCCACAACCAGAATTGGCTTTGGGTGTTGAAGCT
CATACTGATGTTTCTGCTTTGACCTTCATCTTGCATAATATGGTTCCAGGCTTGCAGTTGTTC
TATGAAGGTAAATGGGTTACCGCTAAGTGTGTTCCAGATTCTATCGTTATGCATATCGGTGAT
ACCCTGGAAATTTTGTCTAATGGCAAGTACAAGTCCATCTTGCACAGAGGTTTGGTCAACAAA

GAAAAGGTTAGAATTTCCTGGGCTGTTTTCTGTGAACCACCAAAAGATAAGATCGTCTTGAAA
CCATTGCCAGAGATGGTTTCTGTTGAATCTCCAGCTAAATTTCCACCAAGAACTTTCGCCCAA
CATATCGAACATAAGTTGTTCGGTAAAGAGCAAGAGGAATTGGTTTCCGAAAAGAACGATAGC
GCATGGAGCCACCCGCAGTTCGAAAAGTAA

AtPAL2

ATGGATCAAATCGAAGCTATGTTGTGTGGTGGTGGTGAAAAAACAAAAGTTGCTGTTACTACT
AAGACCTTGGCTGATCCATTGAATTGGGGTTTAGCTGCTGATCAAATGAAGGGTTCTCATTTG
GATGAAGTCAAGAAGATGGTTGAAGAATACCGTAGACCAGTTGTTAACTTAGGTGGTGAGACT
TTGACTATTGGTCAAGTTGCTGCTATTTCTACTGTTGGTGGTTCTGTTAAGGTTGAATTGGCT
GAAACTTCAAGAGCTGGTGTTAAGGCTTCTTCTGATTGGGTTATGGAATCTATGAACAAGGGT
ACTGATTCTTACGGTGTTACTACAGGTTTTGGTGCTACTTCTCATAGAAGAACTAAGAATGGT
ACTGCCTTGCAAACCGAATTGATCAGATTTTTGAACGCCGGTATTTTCGGTAACACCAAAGAA
ACTTGTCATACCTTGCCACAATCTGCTACTAGAGCTGCTATGTTGGTTAGAGTTAACACTTTG
TTGCAAGGTTACTCCGGTATCAGATTCGAAATTTTGGAAGCTATCACCTCCTTGTTGAACCAT
AACATTTCTCCATCTTTGCCATTGAGAGGTACTATTACTGCTTCTGGTGATTTGGTTCCCTTG
TCTTATATTGCTGGTTTGTTGACTGGTAGGCCAAATTCTAAAGCTACTGGTCCAGATGGTGAA
TCTTTGACTGCTAAAGAGGCTTTTGAAAAGGCTGGTATCTCTACTGGTTTTTTCGACTTGCAA
CCTAAAGAAGGTTTGGCTTTGGTTAATGGTACAGCTGTTGGTTCTGGTATGGCTTCTATGGTT
TTGTTTGAAGCTAACGTTCAAGCTGTTTTGGCCGAAGTTTTGTCTGCTATTTTTGCTGAAGTT
ATGTCTGGTAAGCCAGAATTCACTGATCATTTGACCCATAGATTGAAACATCACCCAGGTCAA
ATTGAAGCTGCTGCAATTATGGAACATATCTTGGATGGTTCCAGCTACATGAAGTTGGCTCAA
AAAGTTCACGAAATGGACCCATTGCAAAAGCCAAAACAAGATAGATACGCTTTGAGAACTTCT
CCACAATGGTTGGGTCCACAGATTGAAGTTATTAGACAAGCCACCAAGTCCATCGAAAGAGAA
ATCAATTCTGTTAACGACAACCCCTTGATCGATGTTAGTAGAAACAAAGCTATTCACGGTGGT
AACTTTCAAGGTACTCCAATTGGTGTTTCTATGGATAACACTAGATTGGCTATTGCCGCCATT
GGTAAATTGATGTTTGCTCAGTTTTCCGAGTTGGTCAACGATTTTTACAACAACGGTTTGCCT
TCTAACTTGACCGCTTCTTCTAATCCATCATTGGATTACGGTTTTAAGGGTGCCGAAATTGCT
ATGGCTTCATACTGTTCTGAATTGCAGTATTTGGCTAACCCAGTTACCTCTCATGTTCAATCT
GCTGAACAACATAACCAGGATGTCAATTCCTTGGGTTTGATCTCTTCCAGAAAAACCTCTGAA
GCCGTTGACATTTTGAAGTTGATGTCCACTACTTTCTTGGTCGGTATTTGTCAAGCAGTTGAT
TTGAGACACTTGGAAGAAAACTTGAGACAAACCGTTAAGAACACCGTTTCTCAAGTTGCCAAA
AAGGTTTTGACTACCGGTATTAACGGTGAATTGCATCCATCCAGATTCTGCGAAAAAGATTTG
TTGAAGGTCGTCGATAGAGAACAGGTTTTCACTTATGTTGATGATCCATGCTCTGCTACTTAC
CCATTGATGCAAAGATTGAGACAAGTCATCGTTGATCATGCTTTGTCTAATGGTGAAACCGAA
AAGAACGCTGTTACCTCCATTTTCCAAAAGATTGGTGCTTTCGAAGAAGAATTGAAGGCCGTT
TTGCCAAAAGAAGTTGAAGCAGCTAGAGCAGCTTACGGTAACGGTACTGCTCCAATTCCAAAT
AGAATCAAAGAATGCAGAAGCTACCCCTTGTACAGATTCGTTAGAGAAGAACTTGGTACGAAG
TTGTTGACCGGTGAAAAAGTTGTTTCTCCAGGTGAAGAATTCGACAAGGTTTTTACTGCTATG
TGTGAAGGCAAGTTGATCGATCCTTTGATGGATTGCTTGAAAGAATGGAATGGTGCCCCAATT
CCTATTTGCTAA

AtTT19

ATGGTCGTTAAGTTGTACGGTCAAGTTACTGCTGCTTGTCCACAAAGGGTTTTGTTGTGTTTT
TTGGAAAAGGGTATCGAGTTCGAGATCATCCACATTGATTTGGATACCTTCGAACAGAAGAAG
CCAGAACATTTGTTGAGACAACCATTTGGTCAAGTTCCAGCTATTGAAGATGGTGACTTTAAG
TTGTTCGAATCCAGAGCTATTGCTAGATATTACGCTACTAAGTTCGCTGACCAAGGTACTAAT
TTGTTGGGTAAATCCTTGGAACATAGAGCCATAGTTGATCAATGGGCTGATGTTGAAACTTAC
TACTTCAACGTTTTGGCTCAGCCATTGGTTATCAACTTGATTATCAAACCCAGGTTGGGTGAA
AAGTGTGATGTTGTTTTGGTCGAAGATTTGAAGGTTAAGTTGGGTGTTGTCTTGGACATCTAC
AACAACAGATTGTCCTCTAACAGATTCTTGGCTGGTGAAGAATTCACTATGGCTGATTTGACT
CACATGCCAGCTATGGGTTATTTGATGTCTATCACCGATATTAACCAGATGGTTAAGGCTAGA
GGTTCTTTTAATAGATGGTGGGAAGAAATCTCCGATAGACCATCTTGGAAAAAGTTGATGGTT
TTGGCTGGTCACTGA

CrCPR

ATGGATTCCAGTTCCGAGAAGTTGAGTCCTTTCGAATTGATGTCAGCTATTTTGAAGGGAGCT
AAGTTAGATGGATCAAACTCTTCAGATTCTGGCGTAGCTGTCTCTCCTGCAGTCATGGCTATG
TTGTTGGAAAATAAAGAATTAGTTATGATATTGACAACCTCAGTTGCCGTGTTGATCGGTTGT
GTTGTGGTATTGATCTGGAGAAGAAGTAGTGGTAGTGGAAAGAAGGTTGTCGAGCCTCCAAAA
TTGATAGTTCCTAAGTCAGTGGTAGAACCAGAAGAGATTGATGAAGGCAAGAAGAAATTTACT
ATCTTCTTTGGAACTCAAACAGGTACAGCAGAAGGTTTCGCTAAAGCATTGGCTGAAGAGGCC
AAAGCAAGATATGAGAAGGCTGTTATAAAAGTGATTGATATTGACGATTATGCAGCAGACGAT
GAGGAATACGAAGAAAAGTTTAGGAAGGAAACATTGGCCTTCTTTATCTTAGCAACATATGGT
GATGGTGAACCAACCGATAATGCTGCAAGATTTTACAAATGGTTCGTTGAGGGCAACGACAGA
GGAGATTGGTTGAAGAATTTACAGTATGGAGTTTTCGGCTTAGGTAATAGGCAATACGAGCAT
TTCAATAAAATTGCAAAGGTTGTTGATGAGAAAGTCGCCGAACAAGGCGGCAAAAGGATTGTT
CCTTTAGTCTTAGGAGACGACGATCAATGCATTGAAGATGACTTTGCTGCTTGGAGGGAAAAT

GTTTGGCCTGAATTAGACAATTTGTTAAGGGATGAAGATGACACTACAGTCTCCACTACCTAC
ACTGCTGCCATCCCTGAATATAGAGTTGTTTTCCCTGATAAATCTGATTCTTTAATAAGTGAA
GCAAACGGCCATGCAAATGGCTATGCTAACGGCAATACTGTTTATGATGCTCAACACCCTTGC
AGATCTAATGTTGCTGTTAGAAAAGAATTACACACTCCTGCCAGTGATAGATCATGTACTCAC
TTGGATTTCGATATCGCCGGTACTGGTTTATCATATGGAACAGGTGATCATGTAGGTGTATAT
TGCGATAATTTGTCCGAAACTGTTGAAGAGGCAGAAAGATTATTAAACTTACCACCTGAGACA
TACTTCAGTTTGCATGCTGATAAAGAAGATGGCACCCCATTGGCAGGATCAAGTTTACCACCA
CCATTTCCACCATGTACATTGAGGACTGCTTTAACAAGATACGCTGATTTGTTGAATACTCCA
AAGAAGAGTGCTTTATTGGCATTGGCCGCCTACGCCTCCGACCCTAATGAAGCCGACAGGTTG
AAATACTTGGCCTCACCTGCCGGTAAGGATGAGTACGCTCAATCATTAGTAGCCAATCAAAGG
TCTTTATTGGAAGTTATGGCAGAATTTCCATCAGCCAAACCTCCATTGGGTGTTTTCTTTGCT
GCCATTGCTCCAAGATTGCAGCCAAGATTTTACTCAATATCTTCATCTCCCAGGATGGCACCA
TCTAGAATACACGTCACTTGTGCATTGGTATATGAAAAGACACCAGGTGGCAGAATTCATAAA
GGAGTGTGTTCCACCTGGATGAAGAACGCAATCCCATTAGAAGAGTCCAGGGATTGCTCTTGG
GCACCAATCTTTGTGAGACAATCTAACTTTAAATTGCCCGCTGACCCCAAGGTGCCCGTGATA
ATGATAGGTCCCGGTACTGGATTAGCTCCCTTTAGAGGTTTCTTACAAGAAAGGTTAGCCTTA
AAAGAAGAAGGAGCTGAATTGGGTACAGCTGTGTTCTTCTTTGGTTGTAGAAACAGAAAGATG
GATTACATTTATGAAGACGAATTAAACCATTTCTTAGAAATTGGTGCTTTGTCTGAATTGTTA
GTAGCTTTCTCCAGAGAAGGTCCCACCAAACAATATGTACAGCATAAGATGGCTGAGAAGGCT
TCTGATATTTGGAGAATGATTTCTGACGGTGCATATGTATATGTGTGTGGTGATGCTAAGGGT
ATGGCAAGAGACGTCCATAGAACATTACACACCATTGCACAAGAGCAAGGTTCTATGGACTCC
ACCCAGGCAGAGGGTTTCGTCAAGAACTTACAGATGACCGGTAGATATTTAAGAGACGTCTGG
TGA

FaA3GT2

ATGGCTTCTAATCAAGCTGGTGGTCATGTTGCTGTTTTGGCTTTTCCATTTTCTACTCATGCT
GCCCCTTTGTTGAATATTGTCTGTAGATTGGCTGCTGCTGCTCCATCTACTTTGTTTTCTTTT
TTCAACACCAAACAATCCAACTCCTCCATTTTGGCTTCAGATACTTCTGTTTTGAGATACACC
AACGTTTGCGTTTGTGAAGTTGCTGATGGTGTTCCAGAAGGTTACGTTTTTGTTGGTAAACCA
CAAGAGGATATCGAGTTGTTTATGAAGGCTGCTCCAGATAACTTCAGAAAATGTTTGGAAGCT
AGCGTTGCTGAATCTGGTAGAGAAGTTTCTTGTTTGGTTACCGATGCTTTTTTTTGGTTTGGT
GCTCACATGGCTGATGATATGGGTGGTGTTCCTTGGGTTCCTTTTTGGACTGCTGGTCCAGCT
TCTTTGTCTGCTCATGTTCATACTGATTTGATCAGAAACACTACCTCTGGTGATTGCCATGAC
GAAAAAGAAACCATTACTGTTATTGCCGGTATGTCCAAAGTTAGACCACAAGATTTGCCAGAA
GGTATCATTTTCGGTAACTTGGAGTCTTTGTTCTCCAGAATGTTGCATCAAATGGGTTTGATG
TTGCCATTAGCTACTGCCGTTTTTATCAACTCCTTTGAAGAATTGGACCCAGTTATCACCAAC
GACTTGAAGTCTAAGTTCAAGAGGTTTTTGAACGTTGGTCCATTGGATTTGTTGGAACCTACT
GCTTCTGCTGCTACTACTACACCACAAACTGCTGAAGCTGTTGCTGGTGATGGTTGTTTGTCT
TGGTTGGATAAGCAAAAAGCTGCCTCTGTTGTTTACGTTTCTTTCGGTTCTGTTACTAGACCA
TCTCCAGAGGAATTGATGGCTTTGGCTGAAGCCTTAGAAGCCTCAAGAGTTCCATTTTTGTGG
TCCTTGAGAGACAATTTGAAGAACCCACAATTGGACGAGTTTTTGTCCAAGGGTAAATTGAAC
GGTATGGTTGTACCTTGGGCTCCACAACCACAAGTTTTGGCTCATGGTTCTGTTGGTGCTTTT
GTTACTCATTGTGGTTGGAACTCTGTTTTGGAATCTGTTGCCGGTGGTGTACCATTGATATGT
AGACCATTTTTCGGTGACCAAAAGTTGAACGCTAGAATGGTTGAAGATGTCTGGAAGATTGGT
TTGAGATTGGAAGGTGGTGTTTTCACCAAGAATGGTATGTTGAAGTCCTTGGACATGTTGTTG
TCTCAAGATAAGGGTACGAAGATGAAGAACAAGATCCATACCTTGAAGCAATTGGCTCAACAA
GCTGTTGAACCTAAAGGTTCTTCTACCAGAAACTTCGAATCCTTGTTGGAAATGGCTACTACC
AACTGA

MdCHS2

ATGGTTACCGTTGAAGAAGTTAGAAAGGCTCAAAGAGCTGAAGGTCCAGCTACTGTTTTGGCT
ATTGGTACTGCTACTCCACCAAATTGTGTTGATCAAGCTACTTACCCAGACTACTACTTCAGA
ATTACCAACTCTGAACACAAGACCGAGCTGAAAGAAAAGTTCCAAAGAATGTGCGACAAGTCC
ATGATCAAGACCAGATATATGTACTTGACCGAGGAAATCCTAAAAGAAAACCCAACTGTTTGT
GAGTACATGGCTCCATCTTTGGATGCTAGACAAGATATGGTTGTTGTTGAAGTTCCAAGGTTG
GGTAAAGAAGCTGCTACAAAAGCTATCAAAGAATGGGGTCAACCTAAGTCCAAGATTACCCAT
TTGGTTTTCTGTACTACCTCCGGTGTTGATATGCCAGGTGCTGATTATCAATTGACTAAGTTG
TTGGGTCTAAGACCATCCGTTAAGAGATTGATGATGTACCAACAAGGTTGTTTTGCTGGTGGT
ACAGTTTTGAGATTGGCTAAAGATTTGGCCGAAAACAACAAAGGTGCTAGAGTTTTGGTTGTC
TGCTCTGAAATTACTGCTGTTACTTTTAGAGGTCCATCTGATACTCACTTGGATTCTTTGGTT
GGTCAAGCCTTGTTTGGTGATGGTGCTGCTGCTGTTATTATTGGTTCTGATCCAGTTCCAGAA
GTCGAAAAGCCATTATTCGAATTGGTTTCTGCTGCCCAAACTATCTTGCCAGATTCAGATGGT
GCTATTGATGGTCATTTGAGAGAAGTTGGTTTGACCTTCCATTTGTTGAAAGATGTCCCAGGC
TTGATCTCCAAGAACATTGAAAAATCTTTGAACGAGGCCTTCAAGCCAATTGGTATTTCTGAT
TGGAATTCCTTGTTCTGGATTGCTCATCCAGGTGGTCCAGCAATTTTGGATCAAGTTGAATCT
AAGTTGGCCTTGAAGCCAGAAAAATTGGAAGCTACTAGACAGGTTTTGTCCGATTACGGTAAT

ATGTCATCTGCTTGCGTTTTGTTCATCTTGGATGAAGTCAGAAGAAAGTCTGCTGAGAAGGGT
TTGAAAACTACTGGTGAAGGTTTGGAATGGGGTGTTTTGTTTGGTTTTGGTCCAGGTTTGACT
GTTGAAACTGTTGTCTTGCATTCTGTTGGTGCTTGA

MdF3H
ATGGCTCCAGCTACTACTTTGACTTCTATTGCTCACGAAAAGACCTTGCAACAAAAGTTCGTT
AGAGATGAAGATGAGAGGCCAAAGGTTGCTTACAATGACTTTTCTAACGAGATCCCCATTATT
TCCTTGGCCGGTATTGATGAAGTTGAAGGTAGAAGAGGTGAAATCTGCAAAAAAATCGTTGCT
GCTTGTGAAGATTGGGGTATCTTTCAAATAGTTGATCATGGTGTTGACGCCGAATTGATTTCT
GAAATGACTGGTTTGGCTAGAGAATTCTTTGCTTTGCCATCTGAAGAAAAGTTGAGGTTCGAT
ATGTCTGGTGGTAAGAAAGGTGGTTTCATAGTTTCTTCACACTTACAAGGTGAAGCCGTTCAA
GATTGGAGAGAAATCGTTACTTACTTCTCTTACCCAATCAGACACAGAGATTATTCAAGATGG
CCAGATAAGCCAGAGGCTTGGAGGGAAGTTACTAAGAAATATTCCGATGAGTTGATGGGTTTA
GCCTGTAAGTTGTTGGGTGTTTTGTCTGAAGCTATGGGATTAGATACTGAAGCCTTGACTAAG
GCTTGTGTTGATATGGATCAAAAGGTTGTCGTTAACTTCTACCCAAAATGTCCACAACCAGAT
TTGACTTTGGGTTTGAAGAGACATACTGATCCAGGTACTATTACCTTGTTGTTGCAGGATCAA
GTTGGTGGTTTACAAGCTACTAGAGATGATGGTAAGACTTGGATTACTGTTCAACCTGTTGAA
GGTGCTTTCGTTGTTAATTTGGGTGATCACGGTCATTTGTTGTCTAACGGTAGATTCAAGAAC
GCTGATCATCAAGCTGTTGTCAACTCTAATTCCTCCAGATTGTCTATTGCCACCTTTCAAAAT
CCAGCTCAAGAAGCTATAGTCTACCCATTGTCTGTTAGAGAAGGTGAAAAGCCAATTTTGGAA
GCTCCAATTACTTACACCGAGATGTACAAAAAGAAGATGTCCAAGGATTTGGAATTGGCCAGG
TTGAAAAAGTTGGCCAAAGAACAACAATCTCAGGACTTGGAAAAAGCCAAAGTTGATACAAAG
CCAGTTGATGATATTTTCGCCTAA

OsF3'H
ATGGATGTTGTCCCATTGCCATTGTTGTTAGGTAGTTTAGCTGTTTCCGCAGCCGTCTGGTAT
TTAGTGTACTTCTTAAGGGGCGGATCTGGAGGTGATGCTGCTAGAAAGAGAAGGCCCTTACCT
CCTGGACCTAGAGGCTGGCCAGTGTTAGGAAATTTACCTCAATTAGGCGATAAACCTCATCAT
ACAATGTGCGCATTAGCCAGACAGTATGGTCCATTATTTAGATTGAGGTTCGGATGTGCAGAA
GTTGTTGTAGCCGCATCAGCTCCAGTCGCAGCTCAATTCTTGAGAGGTCATGATGCCAACTTT
TCTAACAGACCACCAAACTCAGGCGCAGAACACGTAGCCTATAATTATCAAGATTTGGTCTTT
GCTCCCTACGGTGCAAGATGGAGAGCCTTGAGAAAATTATGTGCTTTGCATTTATTCTCAGCT
AAGGCTTTGGACGACTTGAGGGCAGTCAGAGAGGGAGAAGTCGCCTTAATGGTTAGAAATTTA
GCAAGACAACAGGCCGCATCCGTGGCTTTAGGTCAGGAAGCAAATGTGTGTGCAACTAACACC
TTAGCTAGAGCCACAATTGGCCATAGGGTTTTCGCAGTGGATGGTGGAGAAGGTGCTAGGGAG
TTCAAGGAAATGGTTGTGGAATTGATGCAATTAGCCGGTGTGTTTAACGTAGGTGATTTTGTC
CCAGCTTTGAGGTGGTTGGATCCACAAGGCGTTGTCGCTAAAATGAAGAGATTGCATAGAAGA
TATGATAACATGATGAATGGTTTCATTAATGAAAGGAAGGCTGGCGCCCAGCCAGATGGTGTA
GCAGCTGGCGAACACGGTAACGATTTGTTATCAGTTTTGTTGGCAAGAATGCAAGAAGAACAG
AAGTTAGACGGTGATGGTGAAAAGATTACCGAGACCGATATCAAAGCCTTGTTGTTGAATTTG
TTCACTGCTGGCACAGATACTACTAGTTCTACAGTTGAATGGGCATTAGCCGAATTAATCAGA
CACCCAGATGTATTAAAAGAAGCTCAACATGAGTTGGATACAGTGGTGGGAAGAGGTAGATTG
GTCAGTGAATCAGATTTACCCAGATTACCATATTTAACAGCTGTGATTAAAGAAACATTTAGA
TTACACCCATCTACTCCTTTGTCTTTGCCAAGAGAGGCCGCAGAAGAGTGTGAAGTAGATGGT
TATAGGATACCAAAAGGTGCCACTTTATTAGTAAATGTTTGGGCTATAGCAAGAGACCCCACT
CAGTGGCCAGACCCTTTACAATACCAACCATCTAGATTCTTGCCAGGTAGAATGCATGCTGAC
GTAGACGTTAAAGGTGCAGACTTTGGTTTAATACCATTTGGAGCTGGTAGAAGGATTTGCGCA
GGCTTATCCTGGGGTTTAAGAATGGTTACTTTAATGACTGCTACATTGGTTCACGGTTTTGAC
TGGACCTTGGCCAATGGAGCTACCCCTGACAAATTGAATATGGAGGAGGCTTACGGTTTGACC
TTGCAAAGGGCTGTACCTTTGATGGTCCAACCTGTTCCTAGGTTGTTGCCTAGTGCTTACGGA
GTTTAG

MsCHI
ATGGCTGCTTCTATTACTGCCATTACCGTTGAAAATTTGGAATACCCAGCTGTTGTTACTTCT
CCAGTTACTGGTAAGTCTTACTTTTTAGGTGGTGCTGGTGAAAGAGGTTTGACTATTGAAGGT
AACTTCATTAAGTTCACCGCCATCGGTGTTTACTTGGAAGATATTGCTGTTGCTTCTTTGGCT
GCTAAATGGAAGGGTAAATCCTCCGAAGAATTATTGGAAACCTTGGACTTCTACAGGGATATT
ATCTCTGGTCCATTCGAAAAGTTGATCAGGGGTTCTAAGATCAGAGAATTGTCTGGTCCAGAA
TACTCCAGAAAGGTTATGGAAAATTGCGTTGCCCATTTGAAGTCTGTTGGTACTTATGGTGAT
GCTGAAGCTGAAGCTATGCAAAAATTTGCTGAAGCCTTTAAGCCAGTTAATTTTCCACCAGGT
GCTTCCGTTTTTTACAGACAATCTCCAGATGGTATCTTGGGCTTGTCTTTTTCACCAGATACT
TCCATTCCAGAAAAAGAAGCTGCCTTGATTGAAAACAAGGCTGTTTCTTCTGCTGTCTTGGAA
ACTATGATTGGTGAACATGCTGTTTCCCCAGATTTGAAAAGATGTTTAGCTGCTAGATTGCCC
GCTTTGTTGAATGAAGGTGCTTTTAAGATTGGCAACTAA

PhAN9
ATGGTTGTTAAGGTTCATGGTTCTGCTATGGCTGCTTGTCCACAAAGAGTTATGGTTTGCTTG
ATTGAATTGGGTGTCGACTTCGAATTGATCCACGTTGATTTGGATTCCTTGGAACAAAAGAAG
CCAGAATTCTTGGTCTTGCAACCATTTGGTCAAGTTCCAGTTATTGAAGATGGTGACTTTAGG

TTGTTCGAATCCAGAGCTATTATTAGGTACTACGCTGCTAAGTACGAAGTCAAAGGTTCTAAA
TTGACTGGTACTACCTTGGAAGAAAAAGCCTTGGTTGATCAATGGTTGGAAGTCGAATCTAAC
AACTACAACGATCTGGTTTACAACATGGTGTTGCAGTTGTTGGTTTTTCCAAAGATGGGTCAA
ACTTCCGATTTGACTTTGGTTACTAAGTGCGCTAACAAGTTGGAAAACGTGTTCGATATCTAC
GAACAGAGGTTGTCTAAGTCTAAATACTTGGCTGGTGAGTTCTTCTCTTTGGCTGATTTGTCT
CATTTGCCATCCTTGAGATTCTTGATGAATGAAGGTGGTTTCTCCCATTTGGTTACCAAAAGA
AAATGCTTGCACGAATGGTACTTGGACATTTCCTCAAGAGATTCATGGAAAAAGGTCCTGGAT
CTGATGATGAAGAAGATTTCCGAAATTGAGGCCGTTTCCATTCCAGCTAAAGAAGAAGCTAAG
GTCTGA

PhLDOX

ATGGTTAACGCTGTTGTTACTACCCCATCAAGAGTTGAATCTTTGGCTAAGTCTGGTATTCAG
GCCATTCCAAAAGAATACGTTAGACCACAAGAAGAATTGAACGGTATCGGTAACATTTTCGAG
GAAGAAAAAAAAAGACGAAGGTCCACAAGTTCCAACCATCGATTTGAAAGAAATTGACTCCGAG
GACAAAGAAATCAGAGAAAAGTGTCACCAATTGAAAAAGGCTGCTATGGAATGGGGTGTTATG
CATTTGGTTAATCACGGTATCTCCGACGAATTGATCAACAGAGTTAAGGTTGCTGGTGAAACC
TTTTTCGATCAACCAGTCGAAGAGAAAGAAAAGTACGCTAATGATCAAGCCAACGGTAATGTT
CAAGGTTACGGTTCTAAATTGGCTAACTCTGCTTGTGGTCAATTGGAATGGGAAGATTACTTT
TTCCATTGCGCTTTCCCAGAAGATAAGAGAGATTTGTCTATCTGGCCAAAGAACCCAACTGAT
TATACTCCAGCTACTTCTGAATACGCCAAGCAAATTAGAGCTTTGGCTACTAAGATTCTGACC
GTCTTGTCTATTGGTCTAGGTTTGGAAGAAGGTAGATTGGAAAAAGAAGTTGGTGGCATGGAA
GATTTGTTGTTGCAAATGAAGATCAACTACTACCCAAAGTGTCCACAACCAGAATTGGCTTTG
GGTGTTGAAGCTCATACTGATGTTTCTGCTTTGACCTTCATCTTGCATAATATGGTTCCAGGC
TTGCAGTTGTTTTACGAAGGTCAATGGGTTACTGCTAAGTGTGTTCCAAACTCCATTATCATG
CATATCGGTGATACCATCGAGATTTTGTCTAATGGCAAGTACAAGTCCATATTGCACAGAGGT
GTTGTCAACAAAGAAAAGGTTAGATTCTCCTGGGCTATTTTCTGTGAACCACCAAAAGAAAAG
ATCATCTTGAAGCCATTGCCAGAAACTGTTACTGAAGCTGAACCACCTAGATTTCCACCAAGA
ACTTTTGCTCAACACATGGCTCATAAGTTGTTCAGAAAGGATGATAAGGATGCTGCCGTTGAA
CATAAGGTTTTCAACGAAGATGAATTGGATACTGCTGCTGAACACAAAGTCCTGAAAAAGGAT
AATCAAGATGCTGTCGCTGAAAACAAGGATATCAAAGAAGATGAGCAATGTGGTCCAGCAGAG
CATAAGGACATCAAAGAGGACGGTCAAGGCGCTGCTGCAGAGAACAAAGTTTTTAAAGAAAAC
AACCAGGATGTCGCCGCTGAAGAATCTAAGTAA

Strep-tag-*PhLDOX*

ATGGCAAGCTGGAGCCACCCGCAGTTCGAAAAGGGTGCAATGGTTAACGCTGTTGTTACTACC
CCATCAAGAGTTGAATCTTTGGCTAAGTCTGGTATTCAGGCCATTCCAAAAGAATACGTTAGA
CCACAAGAAGAATTGAACGGTATCGGTAACATTTTCGAGGAAGAAAAAAAAAGACGAAGGTCCA
CAAGTTCCAACCATCGATTTGAAAGAAATTGACTCCGAGGACAAAGAAATCAGAGAAAAGTGT
CACCAATTGAAAAAGGCTGCTATGGAATGGGGTGTTATGCATTTGGTTAATCACGGTATCTCC
GACGAATTGATCAACAGAGTTAAGGTTGCTGGTGAAACCTTTTTCGATCAACCAGTCGAAGAG
AAAGAAAAGTACGCTAATGATCAAGCCAACGGTAATGTTCAAGGTTACGGTTCTAAATTGGCT
AACTCTGCTTGTGGTCAATTGGAATGGGAAGATTACTTTTTCCATTGCGCTTTCCCAGAAGAT
AAGAGAGATTTGTCTATCTGGCCAAAGAACCCAACTGATTATACTCCAGCTACTTCTGAATAC
GCCAAGCAAATTAGAGCTTTGGCTACTAAGATTCTGACCGTCTTGTCTATTGGTCTAGGTTTG
GAAGAAGGTAGATTGGAAAAAGAAGTTGGTGGCATGGAAGATTTGTTGTTGCAAATGAAGATC
AACTACTACCCAAAGTGTCCACAACCAGAATTGGCTTTGGGTGTTGAAGCTCATACTGATGTT
TCTGCTTTGACCTTCATCTTGCATAATATGGTTCCAGGCTTGCAGTTGTTTTACGAAGGTCAA
TGGGTTACTGCTAAGTGTGTTCCAAACTCCATTATCATGCATATCGGTGATACCATCGAGATT
TTGTCTAATGGCAAGTACAAGTCCATATTGCACAGAGGTGTTGTCAACAAAGAAAAGGTTAGA
TTCTCCTGGGCTATTTTCTGTGAACCACCAAAAGAAAAGATCATCTTGAAGCCATTGCCAGAA
ACTGTTACTGAAGCTGAACCACCTAGATTTCCACCAAGAACTTTTGCTCAACACATGGCTCAT
AAGTTGTTCAGAAAGGATGATAAGGATGCTGCCGTTGAACATAAGGTTTTCAACGAAGATGAA
TTGGATACTGCTGCTGAACACAAAGTCCTGAAAAAGGATAATCAAGATGCTGTCGCTGAAAAC
AAGGATATCAAAGAAGATGAGCAATGTGGTCCAGCAGAGCATAAGGACATCAAAGAGGACGGT
CAAGGCGCTGCTGCAGAGAACAAAGTTTTTAAAGAAAACAACCAGGATGTCGCCGCTGAAGAA
TCTAAGTAA

PtDFR

ATGGGTACTGAAGCTGAAACTGTTTGTGTTACTGGTGCTTCTGGTTTTATTGGTTCCTGGTTG
ATCATGAGGTTGTTGGAAAAAGGTTATGCTGTTAGAGCTACCGTTAGAGATCCAGATAATATG
AAGAAGGTTACCCACTTGTTGGAATTGCCAAAGGCTTCTACTCATTTGACTTTGTGGAAAGCC
GATTTGTCTGTTGAAGGTTCTTACGATGAAGCTATTCAAGGTTGTACTGGTGTTTTCCATGTT
GCTACTCCAATGGACTTCGAATCTAAAGATCCAGAAAACGAAGTTATCAAGCCAACCATTAAC
GGTGTTTTGGATATTATGAGAGCTTGCGCTAACTCTAAGACCGTTAGAAAGATCGTTTTCACT
TCTTCTGCTGGTACTGTTGATGTCGAAGAAAAAAGAAAGCCAGTCTACGATGAATCTTGCTGG
TCTGATTTGGATTTCGTCCAATCTATTAAGATGACCGGTTGGATGTACTTCGTTTCTAAAACT
TTGGCTGAACAAGCTGCTTGGAAGTTCGCTAAAGAAAACAACTTGGACTTCATCTCCATCATT

CCAACTTTGGTTGTTGGTCCATTCATCATGCAATCTATGCCACCATCTTTGTTGACTGCCTTG
TCTTTGATTACTGGTAACGAAGCTCATTACGGTATCTTGAAACAAGGTCATTACGTTCACTTG
GATGACTTGTGTATGTCCCATATCTTCTTGTACGAAAACCCAAAAGCTGAAGGTCGTTACATC
TGTAATTCTGATGATGCCAACATTCACGATCTGGCTAAGTTGTTGAGAGAAAAGTATCCAGAG
TACAACGTTCCAGCTAAGTTCAAGGATATTGACGAAAACTTGGCTTGCGTTGCTTTCTCATCT
AAGAAGTTGACAGATTTGGGTTTCGAGTTCAAGTACTCCTTGGAAGATATGTTTGCTGGTGCA
GTTGAAACCTGTAGAGAAAAGGGTTTGATTCCATTGTCTCACAGAAAGCAAGTCGTCGAAGAA
TGTAAAGAAAATGAAGTTGTTCCCGCCTCCTGA

PtGSTF8
ATGGTGGTGAAAGTTTATGGTCCGGCAGTTGCAGTTTGTCCGCAGCGTGTTATGGCATGTCTG
CTGGAAAAAGGTGTTGAATTTGATCTGGTTCATGTGGATCTGGATAGCGGTGAACAGAAACTG
CCTGAATTTCTGCTGAAACAGCCGTTTGGTCAGGTTCCGGTTGTTGAAGATGGTGATTTTAAA
CTGTTTGAAAGCCGTGCCATCATTCGTTATTATGCAGCCAAATATGAAGATCGTGGTCCGAAT
CTGCTGGGTAATACCCTGGAAGAAAAAGCACTGGTTGATCAGTGGCTGGAAATTGAAGCACAT
AATTTCAATGACCTGGTGTTCAACATTGTGTTTCAGGTTGTTATTCTGCCTCGTATTGGTCAG
CAGGGTGATAGCGAACTGGTTCGTACCTATGAAGAAAAACTGGAAAAGGTGCTGGATGTGTAT
GAAAAACGTCTGAGCAAAAGCAAATATCTGGCAGGCGATAGCTTTACCCTGGCCGATCTGAGC
CATCTGCCTGCAACACGTTATCTGGTTAATGAAGCAGGTCTGGGTCATCTGGTGAAAGATCGT
AAAAAACTGAATGCATGGTGGGAAGATATTAGCAGCCGTCCGGCATGGAAGAAACTGATGAAC
CTGGCAGGTTTTTAA

ScCPR1
ATGCCATTCGGTATTGATAACACCGATTTCACTGTTTTGGCTGGTTTGGTTTTAGCCGTTTTG
TTGTACGTTAAGAGGAACTCCATCAAAGAACTGTTGATGTCTGATGATGGTGATATTACCGCT
GTTTCTTCTGGTAACAGAGATATTGCTCAAGTTGTCACCGAAAACAACAAGAACTATTTGGTC
TTGTACGCTTCTCAAACTGGTACTGCTGAAGATTACGCTAAGAAGTTCTCCAAAGAATTGGTT
GCCAAGTTCAACTTGAACGTTATGTGTGCTGATGTCGAAAACTACGACTTCGAATCTTTGAAC
GATGTTCCAGTCATCGTGTCCATTTTCATTTCTACTTATGGTGAAGGTGATTTCCCAGATGGT
GCTGTTAATTTCGAAGATTTCATCTGTAATGCTGAAGCTGGTGCTTTGTCTAACTTGAGATAC
AATATGTTCGGTTTGGGCAACTCTACCTACGAATTTTTCAATGGTGCTGCTAAGAAGGCTGAA
AAACATTTGTCTGCTGCTGGTGCTATTAGGTTGGGTAAATTGGGTGAAGCTGATGACGGTGCT
GGTACTACCGATGAAGATTATATGGCTTGGAAGGACTCCATCTTGGAAGTTTTGAAAGATGAA
TTGCACTTGGACGAACAAGAAGCTAAGTTCACTTCTCAATTCCAGTACACCGTTTTGAACGAA
ATCACCGATTCTATGTCTTTGGGTGAACCATCTGCTCATTATTTGCCATCTCATCAGTTGAAC
AGAAACGCCGATGGTATTCAATTGGGTCCATTTGATTTGTCCCAACCTTATATTGCTCCAATC
GTTAAGTCCAGGGAACTGTTTTCTTCAAACGATAGAAACTGCATCCACTCCGAATTCGATTTG
TCTGGTTCTAACATTAAGTACTCCACCGGTGATCATTTGGCAGTTTGGCCATCTAATCCATTG
GAAAAGGTTGAGCAATTCCTGTCCATCTTTAATTTGGACCCAGAAACCATCTTCGACTTGAAA
CCATTAGATCCAACTGTCAAGGTTCCATTTCCAACTCCAACTACTATTGGTGCTGCAATCAAA
CACTACTTGGAAATTACTGGTCCAGTTTCCAGACAGTTGTTCTCCTCTTTGATTCAATTTGCT
CCAAACGCTGACGTCAAAGAAAAGTTGACTTTGTTGTCCAAGGACAAGGATCAATTCGCTGTT
GAAATTACCTCCAAGTACTTCAACATTGCTGATGCCTTGAAATACTTGTCTGATGGTGCTAAA
TGGGATACCGTTCCAATGCAATTTTTGGTTGAATCCGTTCCACAAATGACTCCAAGGTACTAC
TCTATCTCTTCCTCTTCATTGTCTGAAAAGCAAACCGTTCATGTTACCTCCATCGTTGAAAAT
TTCCCAAATCCAGAATTGCCAGATGCTCCACCAGTTGTTGGTGTTACTACTAATTTGTTGAGG
AACATCCAATTGGCCCAAAACAACGTTAATATCGCCGAAACTAATTTGCCAGTCCACTACGAT
TTGAATGGTCCAAGAAAGTTGTTCGCCAATTACAAGTTGCCAGTTCACGTTAGAAGAAGCAAT
TTCAGATTGCCATCAAACCCATCTACTCCCGTTATTATGATTGGTCCAGGTACTGGTGTTGCT
CCTTTTAGAGGTTTTATCAGAGAAAGAGTTGCCTTCTTGGAGTCCCAAAAAAAAGGTGGTAAC
AACGTTTCCTTGGGTAAGCACATTTTGTTCTACGGTTCCAGAAACACTGACGATTTCTTGTAT
CAAGATGAATGGCCAGAATACGCCAAAAAGTTGGATGGTTCTTTCGAAATGGTTGTTGCCCAT
TCAAGATTGCCTAACACTAAGAAGGTTTACGTTCAGGATAAGTTGAAGGACTACGAAGATCAA
GTGTTCGAGATGATTAACAACGGTGCTTTCATCTACGTTTGCGGTGATGCTAAAGGTATGGCT
AAAGGTGTTTCTACTGCTTTGGTTGGTATTTTGTCCAGAGGTAAGTCTATTACCACTGATGAA
GCTACCGAGTTGATCAAGATGTTGAAAACTTCTGGTAGATACCAAGAGGATGTCTGGTAA

VvGST1
ATGGCCAACTCTGATCATATCGTTTTGTTGGATTATTCCGCTTCTCCATTCGCTATGAGAGTT
AGATTGGCTTTGGCTGCTAAAGGTATCGAGTACTTGATCAAAGAAGAAGATTTGACCCAGTCC
AAGTCCTCTTTGTTGTTGAAAGTTAACCCAGTGCATCAAAAGATCCCAGTTTTGATTCATAAC
GGTAAGCCAGTTTGCGAGTCCTTGATTATCGTTGAGTACATTGATGAAGTCTGGAAGGATAGA
TGCAGATTATTGCCAGCTGATCCATACCAAAGAGCTAAAGCTAAATTTTGGGCCGATTTCATC
GATAAGATGGTTTACCCATCTTGCTACAATGTTTGGGCTGCTAATGGTGAAGTTCAAGAAGCT
GGTAAGAAAGAATTCATCGACAGGATCAAAATGTTGGAGGGTGAATTGAAGTCTAACCCATAT
TTTGGTGGTGAAACCTTGGGTTTCTTGGATATTGGTTTTCTGCCATCTTACTACTGGTTCCAT
ACTTTCGAAACTTTCGGCAAGTTCAACATTGAAGCTGAATGTCCAAAATTGGTTGCTTGGGGT

	AAAAGATGCATGGAAGAAGAATTCGTTTACACCTCTTTGCCACATCCACATAAGGTTTACGAT
	TTGGTTGTTGACTACAGAAAGAAGATGGGCATCTGA
VvGST2	ATGGCCGTTTTGAAAGTTCATGGTTCTCCAATTTCTACCGCCGTTATGAGAGTTGTTGCTGCC
	TTGTACGAAAAAGGTTTGGAATTTGAATTCGTCACCATCGATATGAAGGCTGGTCAACATAAG
	TCTGAAGCCTTTTTGGCTTTGAATCCATTTGGTCAAGTTCCAGCTTTTGAAGATGGTGACTTG
	AAGTTGTTTGAATCTAGGGCTATTACCCAGTACATTGCTCATGAATATGCTTCCAATGGTACT
	CAATTGATCTGCCCAGATTCTAAGAAAATGGCCATAATGTCCGTTTGGATTGAAGTTGAAGCT
	CACCAATATGATCCACATGCTGGTAAATTGGGTTACGAGTTGTTTTACAAGCCAATGTTCGGT
	CAAACTACTGATCCAGCTGCTGTTGAAGATTTGGAAGCAAAATTGGGTAAAGTCTTGGATGTT
	TACGAAGCCAGATTGACCCAATCCAAATACTTAGGTGGTGATTGCTTTGGTTTGGCTGACTTG
	CATCATTTGCCAACATTGCATTATTTGTTGGGTTCCTCTGCTAAGAAGTTGTTCGATTCAAGA
	CCACATGTTTCTGCTTGGGTTGCTGATATTACTGCTAGACCAGCTTGGGCTAAAGTTATTGCT
	ATGCAAAAGTCCTAA
VvGST3	ATGGTTGTTAAGGTTTACGGTCCAGATTTTGCTTCTGCTAAGAGAGTTTTGGTTTGCTTGATC
	GAAAAAGAAGTCGAGTTCGAAACCTTGCCAATCGATATTATCAAGGGTCAAAACAAGGACCCA
	GAGTTCTTGAAATTGCAACCATTTGGTGTTGTTCCAGTTATCCAAGATGGTGATTACACCTTG
	TTTGAATCCAGAGCTATTATGAGATATTACGCCGAGAAGTACAAGTCCCAAGGTACTGATTTG
	TTGGGTAAGACCATTGAAGAAAGGGGTGTTGTTGAACAATGGTTGGAAGTTGAAGCTCAATCT
	TACCATCCAGCCATTGATAACTTGGTCATCGAAATTTTGTTCGGTAGAAAGAGGGGTATTCCA
	CCAGATGCTAAAGTTATTGAAGAGTCCGAAAAGAAGTTGGCCAAGGTTTTGGATATCTACGAA
	GAAAGGTTGTCCAAGTCTAAATACTTGGCTGGTGATTTCTTCTCCTTGGCTGATTTGTCTCAT
	TTGCCATTCACTAAGTACTTGGCCGATATGGGTAAGATGTACTTGATTGAAGAACGTAAGCAC
	GTTAAGGCTTGGTGGGATGATATTTCTAATAGACCATCCTGGAAGAAGGTGTTCTCTTCAAGA
	TGGCCATTATTGGAGTGA
VvGST4	ATGGTCATGAAGGTTTATGGTCCAGTTAGAGCTGCTTGTCCACAAAGAGTTTTGGCTTGTTTG
	GTTGAAAAGGGTGTTGAATTCGAAGTTGTTCACGTTGATTTGGACTCCGGTGAACAAAAAAGA
	CCAGATTTCTTGTTGAGACAACCATTCGGTCAAGTTCCAGTTGTTGAAGATGGTGATTTTAGG
	TTGTTCGAATCCAGAGCCATCGTTAGATATATTGCTGCTAAATACGCTGAACAAGGTCCAGAT
	TTGTTGGGTAAATCTTTGGAAGAAAAGGCCGTTGTTGATCAATGGTTGGAAGTTGAAGCTCAC
	AACTTTAACGAATTGGTTTACACCTTGGTCATGCAGTTGGTTATTTTGCCAAGAATGGGTGAA
	AGAGGTGATTTGGCTTTGGCTCATACTTGCGAACAAAAGTTGGAAAAGGTTTTCGACGTCTAC
	GAACAGAGATTGTCTAAGTCAAGATATTTGGCCGGTGATTCTTTCACTTTGGCTGATTTGTCT
	CATTTGCCAGCCATTAGATACTTGGTAAAAGAAGCTGGTATGGCCCATTTGGTTACTGAAAGA
	AAATCTGTTTCTGCCTGGTGGGAAGATATTTCTAATAGAGCTGCATGGAAAAAGGTCATGGAA
	TTGGCTGCTTAA
VvGST5	ATGGCCGACGAAATTATCTTGTTGGATTTTTGGCCATCCATGTTCGGTATGAGAGTTAGATTG
	GCTTTGGCTGAAAAAGGTTTGAAGTACGAGTATAGAGAAGAGGACTTGTGGAACAAATCTCCC
	TTGTTGTTGGAAATGAACCCAGTTCATAAGAAGATTCCAGCCTTGATTCATAACGGTAAGCCA
	ATTTGCGAATCCTTGATTATCGTTCAGTACATCGATGAAGTTTGGTGCGATAAGTCTCCATTA
	TTGCCATCTGATCCATACCAAAGAGCACAAGCTAGATTTTGGGCTGATTACATCGATAAGAAG
	TTGTACGAACTGGGCAGAAAAATTTGGTCTACTAAGGGTGAAGAACAAGAAACCGCTAAGAAA
	GAATTCATCGAGTGCTTGAAGTTGTTGGAGGGTGAATTGGGTGAAAAACCATATTTTGGTGGT
	GAGAAGATCGGTTTCGTTGATGTTGCTTTGGTTACTTTCTCTTGTTGGTTCTACGCTTACGAA
	ACTTTCGGTAACTTCTCCATTGAAGCTGAATGCCCAAAATTGATTGCTTGGACTAAGAGGTGC
	ATGGAAAAAGAATCTGTCTCCTCATCTTTGGAAGATCCACATAAGGTTCATGGTTTCATCATG
	GGTATGAGAAAGAGATTCGGTATCGAGTAG
ZmBZ2	ATGACTGCTGGTACTATGAGAGTTTTAGGTGGTGAAGTTTCTCCATTCACTGCTAGAGCTAGA
	TTGGCTTTGGATTTGAGAGGTGTTGCTTACGAATTATTGGATGAACCATTGGGTCCAAAGAAG
	TCTGATAGATTATTGGCTGCTAATCCAGTCTACGGTAAGATTCCAGTTTTGTTGTTGCCAGAT
	GGTAGAGCTATTTGTGAATCTGCTGTTATCGTTCAGTACATCGAAGATGTTGCTAGAGAATCT
	GGTGGTGCTGAAGCTGGTTCTTTGTTATTACCAGATGATCCATACGAAAGAGCCATGCATAGA
	TTTTGGACCGCTTTCATTGATGATAAGTTTTGGCCAGCTTTGGATGCTGTTTCTTTGGCTCCA
	ACTCCAGGTGCTAGAGCACAAGCTGCTGAAGATACAAGAGCTGCTTTGTCTTTGTTGGAAGAA
	GCCTTTAAGGATAGGTCTAACGGTAGAGCTTTTTTCAGTGGTGGTGATGCTGCTCCAGGTTTG
	TTGGATTTGGCTTTAGGTTGTTTTTTGCCAGCATTGAGAGCTTGCGAAAGATTGCATGGTTTG
	TCATTGATTGATGCTTCAGCTACTGCTACTCCTTTGTTAGATGGTTGGTCACAAAGATTTGCT
	GCTCATCCAGCTGCTAAAAGAGTTTTGCCAGATACTGAAAAGGTTGTCCAGTTCACTAGATTC
	TTGCAAGCTCAATTCAGAGTCCACGTTTCTTGA

Supplementary Table 7. Individual datapoints for Fig. 3.

Variant	V_0^{GST} (%WT)			V_0^{DEH} (%WT)		
	Repl. 1	Repl. 2	Repl. 3	Repl. 1	Repl. 2	Repl. 3
V12G	25.2	24.5	22.7	23.5	25.3	24.6
V12W	101.5	93.7	92.8	5.3	6.0	4.3
V12Q	32.4	34.1	31.4	7.3	7.9	5.7
V12N	12.2	11.4	12.2	20.1	23.6	24.9
V12V	106.3	100.2	93.5	114.3	104.1	81.6
V12I	109.5	97.2	90.1	38.2	36.0	33.2
V12A	54.8	46.9	42.0	117.0	102.8	83.1
V12S	19.4	18.6	18.2	81.4	75.9	76.9
V12R	10.0	9.4	9.5	4.5	2.8	3.8
V12D	11.5	10.7	10.1	7.7	4.1	1.7
V12Y	13.6	10.8	11.0	5.8	4.3	4.5
V12L	63.9	61.5	61.0	15.8	13.7	18.7
V12M	66.8	59.0	58.8	6.8	4.1	6.0
V12F	40.5	35.2	34.3	6.2	4.7	6.4
V12E	9.1	11.5	8.4	6.0	4.1	4.0
V12C	28.9	23.5	24.4	92.7	81.0	74.2
V12T	17.4	16.3	17.9	124.1	104.6	106.9
V12P	13.1	12.0	11.2	114.5	105.2	94.4
C13R	9.9	8.6	9.3	4.4	2.1	2.5
C13V	10.1	8.8	9.7	4.4	1.9	2.7
C13W	9.7	8.1	8.5	3.2	2.3	2.9
C13A	160.3	156.6	158.2	50.8	46.4	39.9
C13K	10.1	8.6	9.5	4.4	4.2	3.4
C13L	5.8	9.0	7.2	4.0	3.0	4.2
C13P	10.4	10.6	10.3	4.4	3.8	5.3
C13G	149.0	139.3	139.5	76.4	64.5	61.6
C13C	103.6	97.0	99.5	113.9	103.3	82.8
C13Q	27.4	23.5	23.2	4.6	3.4	4.0
C13S	42.9	41.3	39.6	109.9	101.2	80.5
C13T	28.7	28.3	30.5	20.7	19.3	17.2
C13M	10.2	8.1	8.9	4.9	2.1	2.1
C13N	83.1	79.8	80.4	73.0	74.7	83.0
C13F	9.1	9.3	9.3	4.0	8.5	4.2
N108N	109.9	93.8	96.3	115.8	99.9	84.3
N108Q	41.5	36.1	36.6	31.5	26.9	38.3
N108G	50.6	47.7	50.8	53.2	48.6	48.1
N108A	46.2	42.6	41.0	11.9	11.3	14.1
N108K	12.1	11.2	10.5	4.3	2.7	4.1
N108S	32.6	28.5	27.0	11.2	11.1	14.0
N108H	63.9	59.4	57.0	8.4	5.5	8.1
N108R	13.0	12.6	12.5	4.7	4.1	4.3
N108W	39.0	38.7	39.1	4.3	3.1	3.3
N108E	31.7	24.7	27.6	21.1	17.4	21.6
N108V	10.3	8.4	8.9	3.3	2.7	2.9
N108C	32.3	26.2	26.8	14.3	13.8	17.7
N108Y	46.1	44.6	41.2	6.7	2.5	3.1
N108T	17.0	15.7	15.1	6.8	5.7	7.5
F112R	29.0	27.0	27.3	79.7	72.5	70.2
F112A	65.6	63.9	66.1	112.6	104.2	103.9
F112S	73.5	62.7	64.8	111.4	98.9	101.5

F112V	102.0	91.9	94.9	73.5	67.9	75.0
F112I	143.6	132.4	135.1	39.4	36.1	46.2
F112L	79.5	71.1	75.3	57.5	56.3	65.9
F112F	104.5	97.2	98.3	114.3	101.3	84.4
F112H	170.3	191.2	189.4	83.4	78.1	72.9
F112N	83.0	72.0	80.1	38.2	33.9	40.2
F112E	83.9	68.6	81.6	92.1	85.3	89.0
F112G	115.9	99.6	98.7	83.4	78.3	75.0
F112T	55.4	50.6	50.0	25.1	20.4	27.2
F112L	36.7	56.5	49.2	24.9	37.1	45.7
F112K	25.1	23.8	23.4	100.3	90.9	83.7
F112M	33.5	30.7	30.9	53.9	50.2	59.4
F112D	30.9	27.9	31.1	13.8	12.4	14.4
F112Y	47.6	41.7	44.1	147.1	133.8	113.1
F112W	50.1	43.1	46.2	118.9	103.7	98.6
F112Q	162.3	151.7	164.6	144.5	135.7	127.7
F112P	12.1	12.2	11.5	3.9	3.2	4.8
F112C	63.1	54.9	60.5	91.8	84.4	87.1

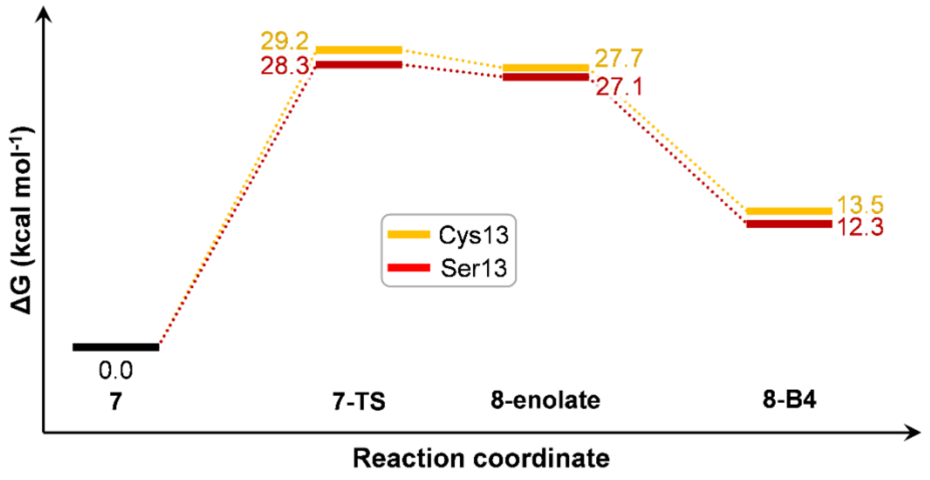

Supplementary Figure 1. Minimum free energy (ΔG) pathways calculated with ONIOM(ωB97X-D/6-311+G(2d,p):ff14SB,gaff2,TIP3P)//ONIOM(B3LYP/6-31G(d):ff14SB,gaff2,TIP3P for the *Pt*GSTF8-catalyzed reaction of flavan-3-on-4-ol ketone (**7**) into cyanidin (**8-B4)**: wild-type enzyme (in yellow), C13S variant (in red). While QM/MM methods would ideally be supported with DFT/MM sampling methods when calculating energy profiles of variants with similar activities, we observed that the calculated free energies (ΔG) and potential energies (ΔE), to which no vibrational corrections was applied, were very similar for both investigated variants (Supplementary Figure 2) and thus plausibly capture the essence of the experimentally verified chemistry (Figure 3b).

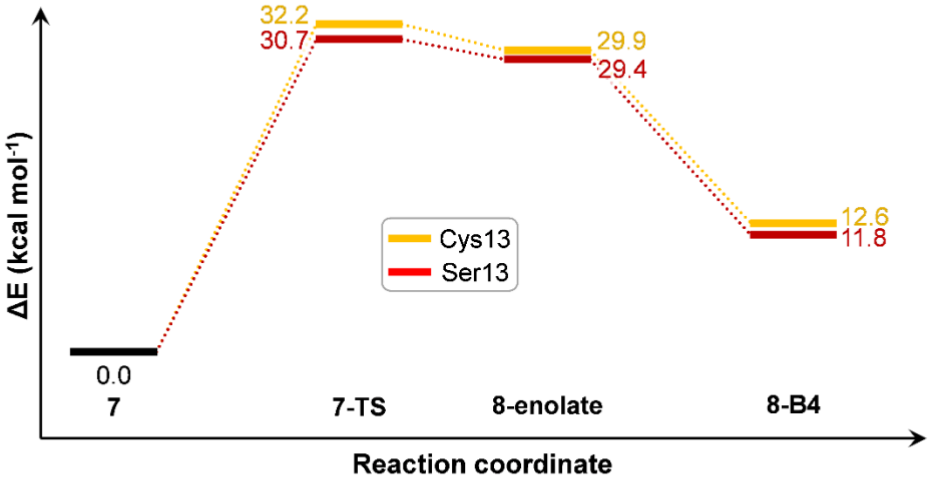

Supplementary Figure 2. Minimum potential energy (ΔE) pathways calculated with ONIOM(ωB97X-D/6-311+G(2d,p):ff14SB,gaff2,TIP3P)//ONIOM(B3LYP/6-31G(d):ff14SB,gaff2,TIP3P for the *Pt*GSTF8-catalyzed reaction of flavan-3-on-4-ol ketone (**7**) into cyanidin (**8-B4)**: wild-type enzyme (in yellow), C13S variant (in red).

Supplementary Table 8. Energies, entropies, and lowest frequencies of the lowest energy calculated structures.[a]

Structure	E_{ONIOM} (Hartree)[b]	E_{ONIOM} + ZPE (Hartree)[c]	H (Hartree)[d]	S (cal mol^{-1} K^{-1})[e]	G (Hartree)[f]	Lowest freq. (cm^{-1})	# of imag. freq.
**7** (WT)	–3052.416614	–3022.467263	–3020.698236	7309.2	–3024.171084	19.2	0
**7-TS** (WT)	–3052.365289	–3022.421828	–3020.653324	7305.9	–3024.124601	–257.3	1
**8-enolate** (WT)	–3052.368973	–3022.423014	–3020.653874	7309.7	–3024.126947	19.7	0
**8-B4** (WT)	–3052.396607	–3022.446662	–3020.677840	7306.9	–3024.149606	19.4	0
**7** (C13S)	–2729.441056	–2699.486986	–2697.718160	7309.0	–2701.190914	19.7	0
**7-TS** (C13S)	–2729.392161	–2699.443401	–2697.675367	7304.3	–2701.145853	–174.9	1
**8-enolate** (C13S)	–2729.394279	–2699.442552	–2697.673913	7311.2	–2701.147718	6.8	0
**8-B4** (C13S)	–2729.422321	–2699.467380	–2697.698676	7308.7	–2701.171261	18.5	0
holo (WT)	–1946.897367	–1917.210779	–1915.458578	7250.6	–1918.903564	19.2	0
holo (C13S)	–1623.923333	–1594.231414	–1592.479479	7251.0	–1595.924642	17.4	0
**7**	–1105.356703	–1105.103731	–1105.084311	140.0	–1105.150844	22.0	0
**8-B4**	–1105.347400	–1105.094053	–1105.074481	139.1	–1105.140576	20.6	0

[a] 1 Hartree = 627.51 kcal mol^{-1}. Thermal corrections at 298.15 K.
[b] Calculated with ONIOM(ωB97X-D/6-311+G(2d,p):ff14SB,gaff2,TIP3P).
[c] Calculated as E_{elec}[ONIOM(ωB97X-D/6-311+G(2d,p):ff14SB,gaff2,TIP3P)] + ZPE[ONIOM(B3LYP/6-31G(d):ff14SB,gaff2,TIP3P)], where ZPE_{corr} is the zero-point energy.
[d] Calculated as E_{elec}[ONIOM(ωB97X-D/6-311+G(2d,p):ff14SB,gaff2,TIP3P)] + H_{corr}[ONIOM(B3LYP/6-31G(d):ff14SB,gaff2,TIP3P)], where H_{corr} is the thermal correction to enthalpy.
[e] Calculated with ONIOM(B3LYP/6-31G(d):ff14SB,gaff2,TIP3P).
[f] Calculated as E_{elec}[ONIOM(ωB97X-D/6-311+G(2d,p):ff14SB,gaff2,TIP3P)] + G_{corr}[ONIOM(B3LYP/6-31G(d):ff14SB,gaff2,TIP3P)], where G_{corr} is the thermal correction to Gibbs free energy.
