# Supplementary material for: The catalytic role of glutathione transferases in heterologous anthocyanin biosynthesis
Source: Nat Catal. 2023 Aug 31;6(10):927–38. doi: 10.1038/s41929-023-01018-y (PMC10593608; doi:10.1038/s41929-023-01018-y)
Supplement: Supplementary file 2 — Reporting Summary [file 41929_2023_1018_MOESM2_ESM.pdf]

nature portfolio

Corresponding author(s): Rebecca M. Buller

Last updated by author(s): Jul 10, 2023

Reporting Summary

Nature Portfolio wishes to improve the reproducibility of the work that we publish. This form provides structure for consistency and transparency in reporting. For further information on Nature Portfolio policies, see our Editorial Policies and the Editorial Policy Checklist.

Statistics

For all statistical analyses, confirm that the following items are present in the figure legend, table legend, main text, or Methods section.

n/a	Confirmed	
☐	☒	The exact sample size (*n*) for each experimental group/condition, given as a discrete number and unit of measurement
☐	☒	A statement on whether measurements were taken from distinct samples or whether the same sample was measured repeatedly
☒	☐	The statistical test(s) used AND whether they are one- or two-sided<br>*Only common tests should be described solely by name; describe more complex techniques in the Methods section.*
☒	☐	A description of all covariates tested
☒	☐	A description of any assumptions or corrections, such as tests of normality and adjustment for multiple comparisons
☐	☒	A full description of the statistical parameters including central tendency (e.g. means) or other basic estimates (e.g. regression coefficient) AND variation (e.g. standard deviation) or associated estimates of uncertainty (e.g. confidence intervals)
☒	☐	For null hypothesis testing, the test statistic (e.g. *F*, *t*, *r*) with confidence intervals, effect sizes, degrees of freedom and *P* value noted<br>*Give P values as exact values whenever suitable.*
☒	☐	For Bayesian analysis, information on the choice of priors and Markov chain Monte Carlo settings
☒	☐	For hierarchical and complex designs, identification of the appropriate level for tests and full reporting of outcomes
☒	☐	Estimates of effect sizes (e.g. Cohen's *d*, Pearson's *r*), indicating how they were calculated

Our web collection on statistics for biologists contains articles on many of the points above.

Software and code

Policy information about availability of computer code

Data collection	HPLC-MS: OpenLAB CDS ChemStation Edition (C.01.07 SR4)<br>High-resolution HPLC-MS: MassHunter Workstation Software (B.08.00)<br>Spectrometry: Sparkcontrol (2.3), UV Lab (4.1.0)<br>MALDI: Compass for flexSeries (1.4)<br>X-ray: Serial synchrotron crystallography (SSX) software suite (CY+ version git-7dabea6a7cf35)<br>QM/MM studies: GOLD 2020.3.0 (CCDC), Gaussian 16 (Gaussian, Inc) and Amber 20 (UCSF).
Data analysis	HPLC-MS: OpenLab CDS (2.4 Update_06)<br>High-resolution HPLC-MS: MassHunter Workstation Software Qualitative Analysis (10.0)<br>Data processing and plotting: Microsoft Excel 365 (2108), Python (3.8.8), Pymol (2.5.2), Chemdraw (19.0.0.22), Adobe Illustrator 2022 (26.3.1), Affinity Photo (1.10.5)<br>Bioinformatics: R (4.1.2), Clustal Omega (1.2.2) , Autodock Vina (1.2.0), PLIP (2.2.0),LigRMSD (1.0), ProtParam (Expasy, no version number available)<br>Structure elucidation: REFMAC (5.8.0352) and Coot (0.9.8.7) within ccp4i2 interface (v8.0.002), autoPROC (1.0.5), XDS (Version January 10, 2022), MolREP (11.9.02), MolProbity (4.5.1), CCP4 7.1.018: comit (0.1.0)<br>QM/MM studies: Python 3.8, Pymol 2.4 and Microsoft Excel 365.

For manuscripts utilizing custom algorithms or software that are central to the research but not yet described in published literature, software must be made available to editors and reviewers. We strongly encourage code deposition in a community repository (e.g. GitHub). See the Nature Portfolio guidelines for submitting code & software for further information.

Data

Policy information about availability of data

All manuscripts must include a data availability statement. This statement should provide the following information, where applicable:

- Accession codes, unique identifiers, or web links for publicly available datasets
- A description of any restrictions on data availability
- For clinical datasets or third party data, please ensure that the statement adheres to our policy

Nucleotide sequences of codon-optimized genes can be found in the Supplementary Table 6. The diffraction images were deposited to Integrated Resource for Reproducibility in Macromolecular Crystallography88 (http://proteindiffraction.org/) and can be accessed using Protein Data Bank entry number 8AGQ. Crystallographic coordinates of the ternary complex of PtGSTF8 have been deposited in the Protein Data Bank as 8AGQ. The binary PtGSTF8 crystal structure used in molecular replacement experiments can be accessed via PDB ID: 5F07. Computed geometries and energies can be accessed through the Zenodo repository (https://doi.org/10.5281/zenodo.8069429). All source data are provided with this manuscript.

Human research participants

Policy information about studies involving human research participants and Sex and Gender in Research.

Reporting on sex and gender	n.a.
Population characteristics	n.a.
Recruitment	n.a.
Ethics oversight	n.a.

Note that full information on the approval of the study protocol must also be provided in the manuscript.

Field-specific reporting

Please select the one below that is the best fit for your research. If you are not sure, read the appropriate sections before making your selection.

☒ Life sciences ☐ Behavioural & social sciences ☐ Ecological, evolutionary & environmental sciences

For a reference copy of the document with all sections, see nature.com/documents/nr-reporting-summary-flat.pdf

Life sciences study design

All studies must disclose on these points even when the disclosure is negative.

Sample size	No sample size calculation was used. In pre-experiments, the experimental data was found to be reproducible and no outliers were observed, therefore 3 independent replicates were performed.
Data exclusions	No data were excluded from the analyses.
Replication	At least three independent replicates were performed and reported for each experiment. Most experiments were performed at least twice with almost identical results, showing a good reproducibility.
Randomization	This study does not involve human/animal subjects and group allocation, therefore randomization was not necessary. Nevertheless, appropriate control experiment were performed.
Blinding	This study does not involve human/animal subjects and group allocation, therefore blinding was not necessary. Nevertheless, appropriate control experiment were performed.

Reporting for specific materials, systems and methods

We require information from authors about some types of materials, experimental systems and methods used in many studies. Here, indicate whether each material, system or method listed is relevant to your study. If you are not sure if a list item applies to your research, read the appropriate section before selecting a response.

Materials & experimental systems

n/a	Involved in the study
☒	☐ Antibodies
☒	☐ Eukaryotic cell lines
☒	☐ Palaeontology and archaeology
☒	☐ Animals and other organisms
☒	☐ Clinical data
☒	☐ Dual use research of concern

Methods

n/a	Involved in the study
☒	☐ ChIP-seq
☒	☐ Flow cytometry
☒	☐ MRI-based neuroimaging
